# Specific Noncovalent Association of Truncated *exo*-Functionalized Triangular Homochiral Isotrianglimines through Head-to-Head, Tail-to-Tail, and Honeycomb Supramolecular Motifs

**DOI:** 10.1021/acs.joc.1c02238

**Published:** 2022-01-14

**Authors:** Agnieszka Janiak, Jadwiga Gajewy, Joanna Szymkowiak, Błażej Gierczyk, Marcin Kwit

**Affiliations:** Faculty of Chemistry, Adam Mickiewicz University, Uniwersytetu Poznańskiego 8, 61- 614 Poznań, Poland

## Abstract

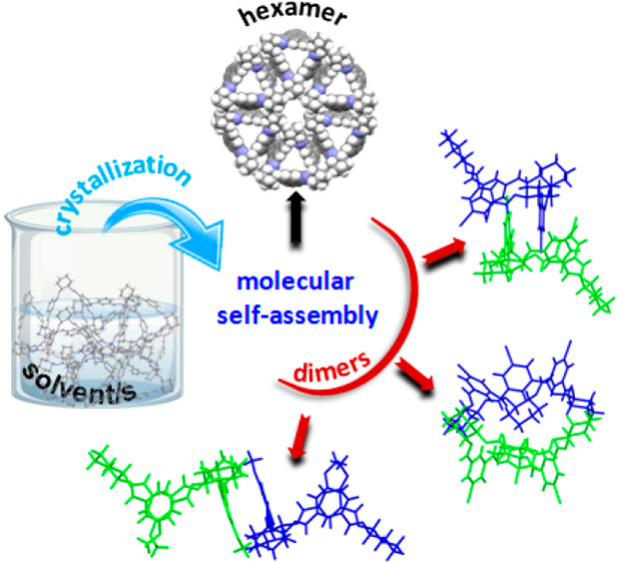

Chiral isotrianglimines were synthesized by the [3 + 3] cyclocondensation of (*R*,*R*)-1,2-diaminocyclohexane with C5-substituted isophthalaldehyde derivatives. The substituent’s steric and electronic demands and the guest molecules’ nature have affected the conformation of individual macrocycles and their propensity to form supramolecular architectures. In the crystal, the formation of a honeycomb-like packing arrangement of the simplest isotrianglimine was promoted by the presence of toluene or *para*-xylene molecules. A less symmetrical solvent molecule might force this arrangement to change. Polar substituents present in the macrocycle skeleton have enforced the self-association of isotrianglimines in the form of tail-to-tail dimers. These dimers could be further arranged in higher-order structures of the head-to-head type, which were held together by the solvent molecules. Non-associating isotrianglimine formed a container that accommodated acetonitrile molecules in its cavity. The calculated dimerization energies have indicated a strong preference for the formation of tail-to-tail dimers over those of the capsule type.

## 1 Introduction

Since the first works by Ružička,^1,2^ macrocycles and, later on, covalent organic cage compounds have become essential compounds in chemistry, material chemistry, crystal engineering, and biochemistry.^3−6^ The continuous development of supramolecular chemistry has resulted in macrocyclic compounds being employed as molecular building blocks in molecular tectonics.^7−10^ This approach allows for the construction of capsules with large internal cavities and is capable of capturing neutral and charged species in addition polymeric structures, where the monomers are bound by noncovalent interactions.^11−15^

In contrast to the low-yielding kinetics-driven synthesis of imide-based highly symmetrical molecular triangles,^16^ the cycloimination reactions between structurally predisposed substrates would lead to products of the same shape and symmetry with, however, as one can even say, much higher quantitative yields.^17−21^ It was as early as 2000 when Gawronski et al. demonstrated the possibility for the quantitative synthesis of a chiral [3 + 3] triangular macrocycle dubbed trianglimine through thermodynamically driven cycloimination of *trans*-(*R*,*R*)-diaminocyclohexane (DACH, **1**) and terephthalaldehyde .^22^ Shortly after, the same and other groups reported syntheses of chiral symmetric macrocyclic polyimines of different shapes (e.g., rhombimines and loopimines) consisting of various functionalities in the aromatic part of the molecule.^17−31^

**Scheme 1:**
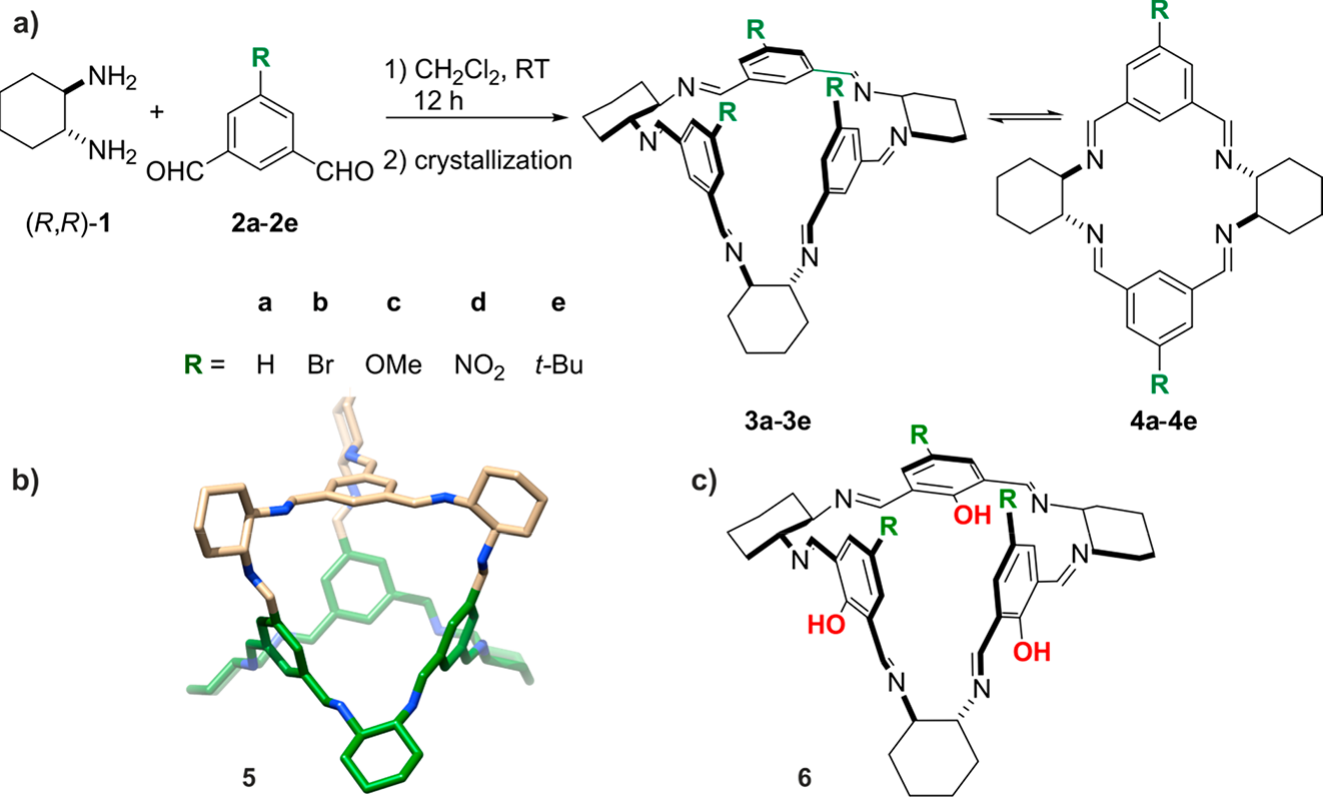
(a) Condensation of isophthalaldehyde derivatives **2a**–**2e** with (*R*,*R*)-DACH (**1**) provided [3 + 3] isotrianglimines **3a**–**3e**, respectively, and their contracted but higher in symmetry [2 + 2] counterparts **4a**–**4e**. (b) Discovered by Skowronek, the *T*_d_-symmetrical molecular polyimine cage of **5** has a multitude of applications. The isotrianglimine motifs in the cage structure are differentiated by colors. (c) Molecular structure of the basic *C*_3_-symmetrical calixsalen **6**.

The 1,3-benzenedicarbaldehyde (isophthalaldehyde, **2**) derivatives are commonly considered “difficult” substrates for cycloiminations.^17−21,32−34^ Whereas terephthalaldehyde itself and its symmetrical derivatives in combination with **1** have provided triangular [3 + 3] products of the highest available *D*_3_ symmetry,^17^ the isophthalaldehydes might lead to the formation of condensation products characterized by the lower *C*_3_ symmetry (isotrianglimine, Scheme 1a).

However, having studied the reactions of various isophthalaldehydes with **1**, Kuhnert concluded that the [3 + 3] cyclocondensation products are formed under kinetic control. In contrast, the smaller *D*_2_-symmetric [2 + 2] macrocycles are thermodynamic products usually formed after prolonged heating in refluxing dichloromethane.^35^ On the contrary, recent results have not supported this hypothesis. For the series of isotrianglimines **3a**–**3e**, which were studied using ion-mobility mass spectrometry (IM-MS), the observed main signals corresponded to the [3 + 3] products and their specific associates, and only very weak signals corresponded to the contracted [2 + 2] systems **4a**–**4e** (Scheme 1a).^36^

It is worth noting that isotrianglimine motifs have been visible in more complex molecules, such as the porous organic cage **5** (Scheme 1b) that was initially synthesized by Skowronek for the first time and then subsequently used in various applications.^37−40^

In recent years, there has been increased interest in using chiral triangular macrocycles in crystal engineering, material chemistry, electrochemistry, catalysis, and the design of porous materials.^41−46^ Only recently, Khashab has proven the self-assembly process of biphenyl containing an enantiopure trianglimine from the gel phase to the crystalline phase.^47^ The study revealed the pivotal role of the guest molecules in the formation of trianglimine-metastable capsules or right-handed helices. In subsequent studies, the highly efficient separation of styrene from ethylbenzene was achieved due to the much better fit of styrene to the trianglimine internal cavity.^48^ The reduced congener of basic trianglimine, namely trianglamine, has shown a propensity to capture of CO_2_ apart from solvent molecules.^49,50^

Those mentioned above and other properties of triangular chiral macrocycles are due to the formation of the “proper crystalline phase”.^47^ Although striving to fill the space as much as possible using the molecules that make up the crystal can still be treated as an axiom, the crystallization process can be perceived from a slightly different perspective as well. For instance, Khashab’s interpretation of the crystallization process relies on a “series of molecular recognition events” taking place simultaneously and cooperatively. In that context, crystallization might be compared to polymerization, leading to the formation of single entities (the crystals) made from individual molecules bound together by noncovalent interactions.^47−50^ The formation of such supramolecular assemblies is often controlled by the host (i.e., macrocycle)–guest (i.e., solvent) interactions, and the presence or absence of the guest molecules has a profound effect on the structure of the crystal.

In principle, learning about the relationship between the shape of the molecule, the functionalities present in its skeleton, and the crystal structure should allow for the prediction and rational design of materials with predetermined properties. However, in the real world these predictions are rather the result of coincidence than the effect of systematic analyses of the possible intermolecular interactions. While possibilities for the formation of various supramolecular architectures in both the solid state and solution by calixsalens structurally similar to isotrianglimines (**6**, Scheme 1c) have been unequivocally confirmed by us and others,^51−55^ little is known to date about the propensity of the neutral isotrianglimines to form supramolecular architectures in the solid state. Only recently has Cooper et al. reported crystal structures of optically pure and racemic **3a**.^56^ In the crystal, the optically pure **3a** molecules form pillars containing solvent molecules in their cavities. A heterochiral pairing strategy introduced porosity when (*rac*)-**3a** was crystallized and packed in the lattice using head-to-head supramolecular motifs. As a result, a porous material was obtained that was characterized by the highest reported SA_BET_ of 355 m^2^ g^–1^ for trianglimine-like macrocycle and exhibited a high selectivity toward the separation of *para*-xylene from the mixture of isomers.^56^

As the further spectacular applications of macrocycle-based materials started from rather basic research, in this study we have paid attention to the possibility of creating various supramolecular motifs using the model isotrianglimines **3a**–**3e**. The results of the initial research with the use of NMR methods (mainly DOSY NMR) showed similarities between calixsalens and isotrianglimines in the formation of supramolecular assemblies.^57^ Yet, none of the applied methods have provided a clear picture regarding the “supramolecular behavior” of neutral isotrianglimines in the solution. Therefore, the X-ray diffraction methods seem to be the methods of choice for “catching” specific noncovalent aggregates of the isotrianglimines. The varied nature of substituents attached to the “tail” of the macrocycles might or might not affect their ability to form supramolecular architectures in the solid state. An additional factor that needs to be taken into account is the propensity of the macrocycles to incorporate (or not) the solvent molecules in the crystal lattice. The mixing of macrocycles that have substituents with totally different electronic properties could lead to hybrid materials containing (or not) both macrocyclic ingredients. Supporting theoretical calculations would provide some structural and energy data related to the stability and structure of a given associate. As the result of this study, a question regarding similarities and differences between isotrianglimines and calixsalens would be addressed. Finally, to keep the discussion as concise as possible, the results less relevant to the main topic are skipped but are discussed briefly in the Supporting Information.

## 2 Results and Discussion

### 2.1 Synthesis of Isotrianglimines **3a**–**3e**

Since the compounds under study are known, we will only sketch the synthesis scheme here. The starting aldehydes were obtained by reducing the respective isophthalic acid methyl esters using lithium aluminum hydride and Swern oxidation of the diols. Since the reduction of dimethyl 5-bromoisopthalate under such conditions gave a partially dehalogenated product, we used a tetrahydrofuran–borohydride complex for the reduction of 5-bromoisopthalic acid. Nitration of isopthaldehyde with the use of red fuming nitric acid and ammonium sulfate was done according to the procedure previously described by Jennings.^58^

The cyclocondensation reactions were performed under an argon atmosphere using equivalent amounts of diamine and dialdehyde in dichloromethane. The concentration of the substrates was kept at 0.02 M, and the reactions ran for 12 h at room temperature. Water was not removed, which prevented equilibrium conditions. The optimal reaction time was determined experimentally for the model reactions between (*R*,*R*)-**1** and aldehydes **2c** and **2d**, respectively. The test reactions were run in NMR tubes in anhydrous CDCl_3_ at room temperature for 120 h. Both aldehydes are characterized by similar reactivities despite the different character of the substituent attached to the aromatic ring. Within 7 h, most of the respective aldehyde had disappeared, and the complete conversion of substrates was achieved within 12 h.

The recorded ^1^H NMR spectra of the crude **3a**–**3e** products showed a complete disappearance of aldehyde signals and the formation of cyclic products. A further flash purification procedure performed by precipitating the macrocycle from the dichloromethane/acetonitrile solution has allowed analytically pure samples consisting of [3 + 3] **3a**–**3e** to be obtained. ESI-TOF mass spectra further confirmed the trimeric structure of the macrocycles, and the molecular ions corresponded to single protonated [M + H]^+^ species. The ^1^H NMR spectra recorded at certain time intervals for chloroform solutions of the isotrianglimines **3b**–**3e** gave an insight into the stability of the macrocycles in solution (see the SI for details). With exception of **3e**, the remaining isotrianglimines turned out to be resistant to the ring contractions for at least 6 h (see Figures S3–S6 in the SI).

**Figure 1:**
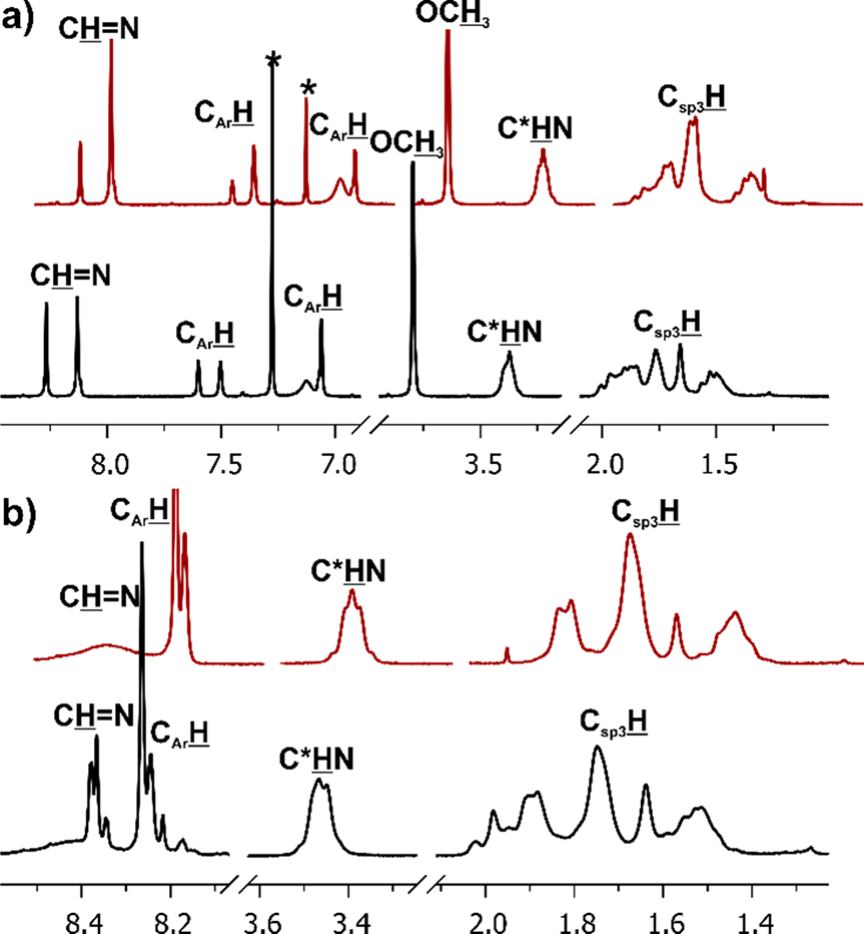
Example of ^1^H NMR spectra of isotrianglimines (a) **3c** and (b) **3d** (anhydrous CDCl_3_, 300 MHz, 20 °C) measured for the crude products of reactions conducted at an elevated temperature (black lines) and for freshly dissolved crystals of the [3 + 3] products (red lines). Asterisks indicate trace solvent peaks.

The formation of the mixture of cyclic products has been visible for the reactions intentionally carried out at an elevated temperature. The mixture of equimolar (*R*,*R*)-**1** and the respective aldehyde was heated in chloroform to reflux (ca. 61 °C) and maintained for 48 h. The direct comparison of the ^1^H NMR spectra measured for crude reaction mixtures and those for authentic samples is shown in Figure 1. In the case of **3c**, the differences in the downfield region between ^1^H NMR spectra measured for freshly dissolved crystals and those for the crude reaction mixture are limited to changes in the relative intensity of the signals. However, in the upfield region of the ^1^H NMR spectrum measured for the crude reaction mixture a multiplication of the C_sp^3^_H signals is visible. More significant changes were observed for **3d**. In both, the upfield and downfield regions of the ^1^H NMR spectrum measured for the crude reaction mixture, the respective signals were multiplied. Notably, we have not been able to shift the expected equilibria between macrocycles toward the smaller [2 + 2] products even after two weeks of continuously heating the reaction mixtures in chloroform.

### 2.2 Solid-State Supramolecular Assemblies of Isotrianglimines

Isotrianglimine crystals (**3a**–**3e**) suitable for single-crystal X-ray diffraction analysis were obtained by slow evaporation from a solution of single or mixed polar and nonpolar solvents at room temperature. Except for **3a**, the crystal structures of isotrianglimines were unknown. Therefore, the crystallization from various solvents was aimed at (i) determining the preference of macrocycles to self-assemble in the crystalline phase and (ii) examining whether the use of certain solvents would induce changes in the arrangement of macrocyclic supramolecular assemblies. As a result of the crystallization process, we have obtained three inclusion forms of **3a** (**3a_1**, **3a_2**, and **3a_3**), two forms of **3b** (**3b_1** and **3b_2**), one each of **3c** and **3e**, and a guest-free phase of **3d**.

**Figure 2:**
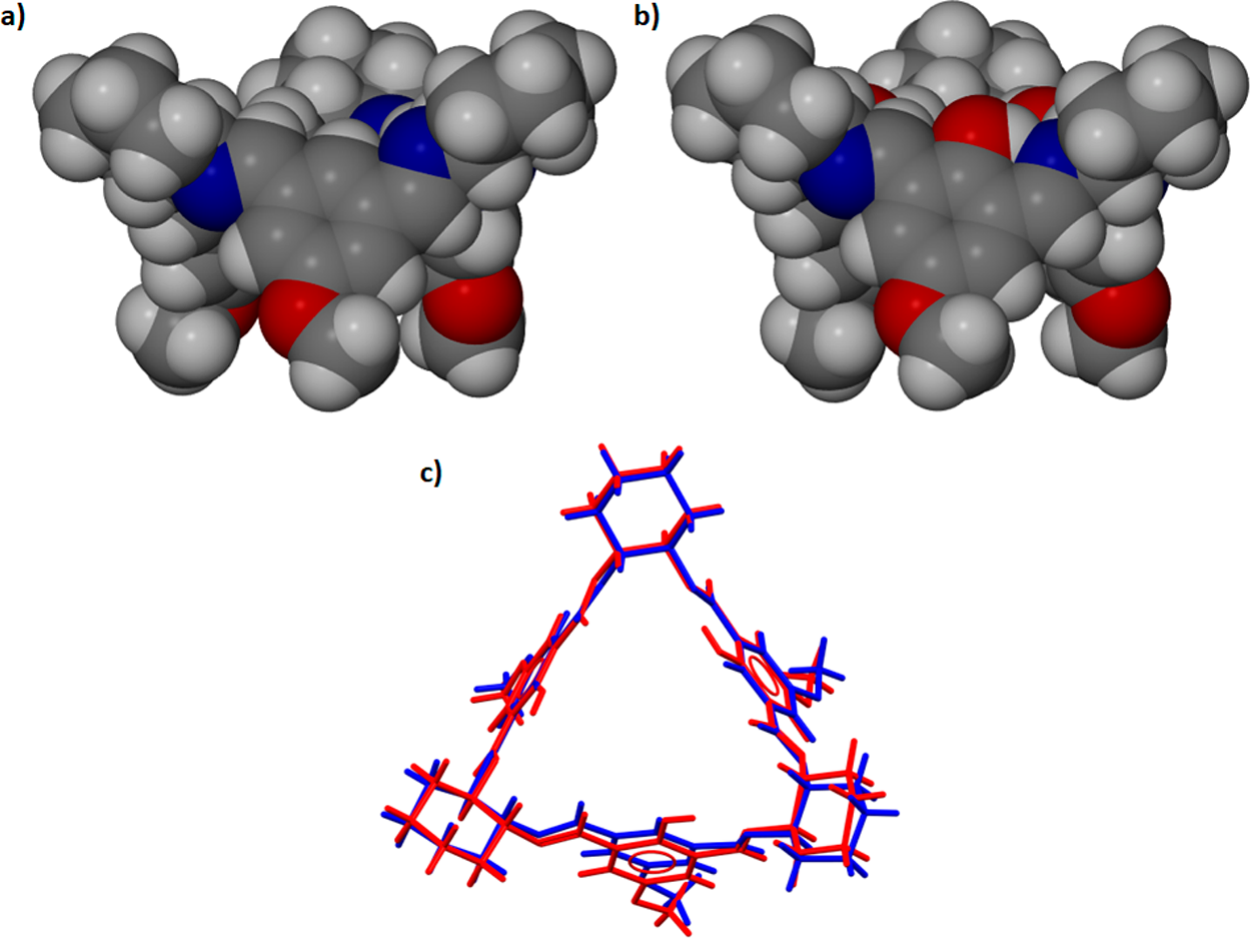
Van der Waals representation of the molecular shape of (a) isotrianglimine and (b) its calixsalen counterpart. Both macrocycles presented here have the same functional groups (OCH_3_) at the C5 position of the 1,3-dialdehyde skeleton. (c) The structural overlay of the isotrianglimine (blue) and calixsalen (red) skeletons.

In terms of its molecular shape, isotrianglimine (Figure 2a) has a vase-like structure and strongly resembles the calixsalen shape with the hydroxyl groups removed (Figure 2b). Such remarkable similarities in shape and dimensions are dictated using a fixed geometry of the building blocks (diaminocyclohexane and aromatic 1,3-dialdehyde) in the synthesis of both macrocycles. Using dialdehydes with similar lengths in turn makes the cavity sizes of the isotrianglimines (ranging from 6.0 to 7.9 Å) almost the same as those of their calixsalen counterparts (ranging from 6.0 to 7.8 Å),^51−55^ as shown in Figure 2c. The upper rim of isotrianglimine comprises imine groups and cyclohexyl rings, while substituents at the C5 position of the 1,3-dialdehyde skeleton are situated on the lower rim. Thus, the upper rim remains hydrophobic by nature, while the character of the lower rim can be controlled by the nature of the substituents attached to the aromatic rings.

We have previously reported that the solid-state self-assembly of enantiomerically pure calixsalens is driven by the type of the substituent at the C5 position, i.e., small or polar groups would lead to the self-assembly of dimers, while bulky and hydrophobic groups would form capsules or unimolecular cages.^51−55^ The question then arises whether the self-assembly of isotrianglimines would be affected by the type of the substituent attached to their skeleton in the same manner since isotrianglimines are so similar to calixalens? In the following paragraph, we have made attempts to answer this question.

**Figure 3:**
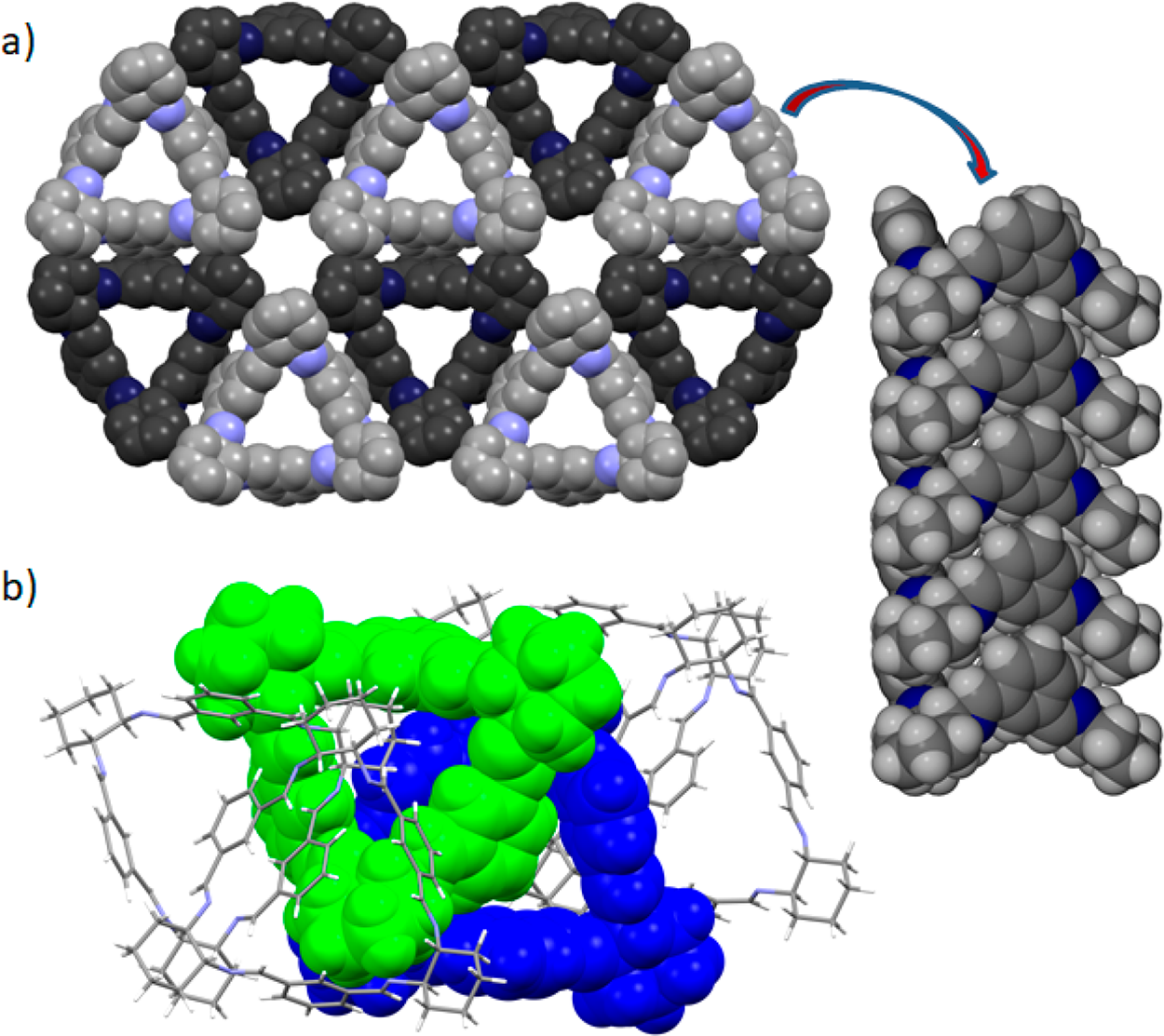
(a) Van der Waals representation of the honeycomb-like arrangement of stacks in the crystals of **3a_1** and **3a_2**. (b) An isolated dimeric motif was observed in the crystals of **3a_3** and **3b_1**. Symmetry-independent macrocycles that constitute the tail-to-tail dimer are shown in different colors as a space-filling model.

When **3a** is crystallized from toluene or *para*-xylene, it tends to self-associate in stacks (see Figure 3a) that propagate along the crystallographic *c* direction. In both crystal structures, the mutual arrangement of stacks is very similar. Each stack is surrounded by three adjacent stacks in contact with each other in the area of an aromatic linker. Consequently, it extends the crystal structure, forming a honeycomb-like arrangement held exclusively by van der Waals interactions. An arrangement of **3a** in stacks leads to the formation of two types of channels in the macrocycle matrix, both of which are fully occupied by a solvent. One channel passes through the cavities of stacked macrocycles, while the other is formed in intermolecular space between six adjacent stacks. The total solvent-accessible volume calculated with the probe radius of 1.5 Å is 18.0% in **3a_1** and 19.1% in **3a_2**, corresponding to unit cell volumes of 395.3 and 416.64 Å^3^, respectively. The formation of similar stacked assemblies was recently reported by Cooper.^56^ Interestingly, the stacking arrangement is not a structural feature of calixsalens and has thus far not been observed in their crystals. On the other hand, such an arrangement is typical to other triangular-shaped macrocycles such as trianglimines^23−31,45,59^ and trianglamines,^60^ the skeletons of which are based on aromatic 1,4-dialdehydes units.

Isotrianglimine **3a** may associate differently in the presence of a bulky solvent such as *meta*-xylene, which does not match the shape of the macrocyclic cavity. During crystal growth, isotrianglimine undergoes packing reorganization from stacks to dimers to incorporate solvent molecules in the internal space, reducing excess free space in the crystal structure. The calculated solvent-accessible volume in crystals of **3a_3** is 9.1%, corresponding to a unit cell volume of 367.59 Å^3^.

In the crystals of **3a_3**, two symmetry-independent molecules form tail-to-tail dimers by the mutual insertion of one of the phenyl rings of each molecule into the cavity of the other macrocycle (mutually penetrating dimers). The dimeric motif is stabilized by π···π stacking interactions with a centroid-to-centroid distance of 3.506(3) Å and a ring offset of 1.07(1) Å and is supported by numerous C–H···π interactions (mean *d*_H···π_ = 2.77 Å). Two pairs of adjacent dimers are mutually offset such that the cyclohexane ring of one of its molecules is located directly above the entrance of the cavity of an adjacent dimer, making each of them isolated aggregates as shown in Figure 3b.

**Figure 4:**
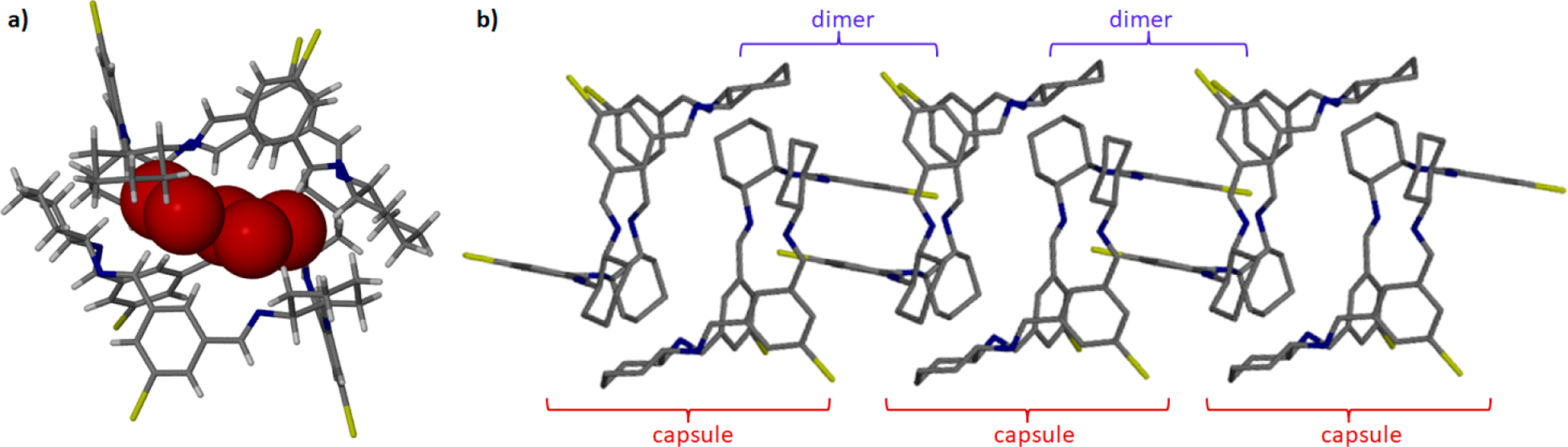
(a) A capsule of **3b** containing water molecules entrapped in the capsule. The positions of water molecules are represented by oxygen atoms in red as space-filling models. (b) Alternate arrangements of two supramolecular dimers and capsules solely in **3b_2**. Hydrogen atoms have been omitted for clarity.

Similarly to **3a_3**, the symmetry-independent macrocycles of **3b_1** self-assemble into isolated tail-to-tail dimers that are stabilized mainly by π···π interactions with a relatively short centroid-to-centroid distance of 3.671(5) Å (aromatic ring offset of 1.21(1) Å) and is supported by multiple C–H···π (mean *d*_H···π_ = 2.84 Å) and Br···π (mean *d*_Br···π_ = 3.353(7) Å) contacts. Changing the solvent used during crystallization from a mixture of CH_2_Cl_2_ and acetonitrile to acetonitrile containing water also leads to the self-assembly of the macrocycles in a tail-to-tail fashion. However, the mutual arrangements of the adjacent dimers vary fundamentally. In **3b_2**, one dimer is situated above the adjacent dimer relative to which it is twisted by about 60°. This arrangement promotes self-aggregation in a head-to-head manner (capsule) between successive dimers. The solvent used for crystallization plays a vital role in forming these capsules. In particular, we used acetonitrile, which was not anhydrous. Consequently, except for acetonitrile, we also found water molecules in the crystal structure. Water mainly occupies the space between the isotrianglimine molecules that are arranged as capsules and is responsible for the stabilization of this motif (Figure 4a). A similar role is played by the hydroxyl groups of the calixsalens in some of their crystal structures. Therefore, we can speculate that the head-to-head motif will only appear in isotrianglimine crystals when solvent molecules containing functional groups capable of forming hydrogen bonds are involved. The expansion of the crystal structures along the [100] direction leads to alternating dimers and capsules, forming pillars as shown in Figure 4b.

The tail-to-tail motif (interdigitating) is commonly observed in the crystals of calixsalens substituted by small or polar groups. It appears as an isolated dimer in the apohost crystals of enantiomerically pure calixalens and in combination with head-to-head dimers in solvated crystals of both enantiomerically pure and racemic calixsalens. This interdigitating motif is also found in the crystal structures of other calix- and bowl-shaped macrocycles such as calix[4]arenes^61,62^ and cyclotriveratrylenes, respectively.^63−65^ However, it is a novelty that isotrianglimines, in addition to the “classic” tail-to-tail dimer, can form a different type of tail-to-tail dimer in the crystals. Such a dimer, which we call the “external” dimer, is not formed by the interdigitation of macrocycles but instead by neighboring macrocycles arranged side by side in an antiparallel fashion. It is noteworthy that the “external” dimer does not appear as an independent or main supramolecular motif. Instead, it is always accompanied by a “classic” dimer.

**Figure 5:**
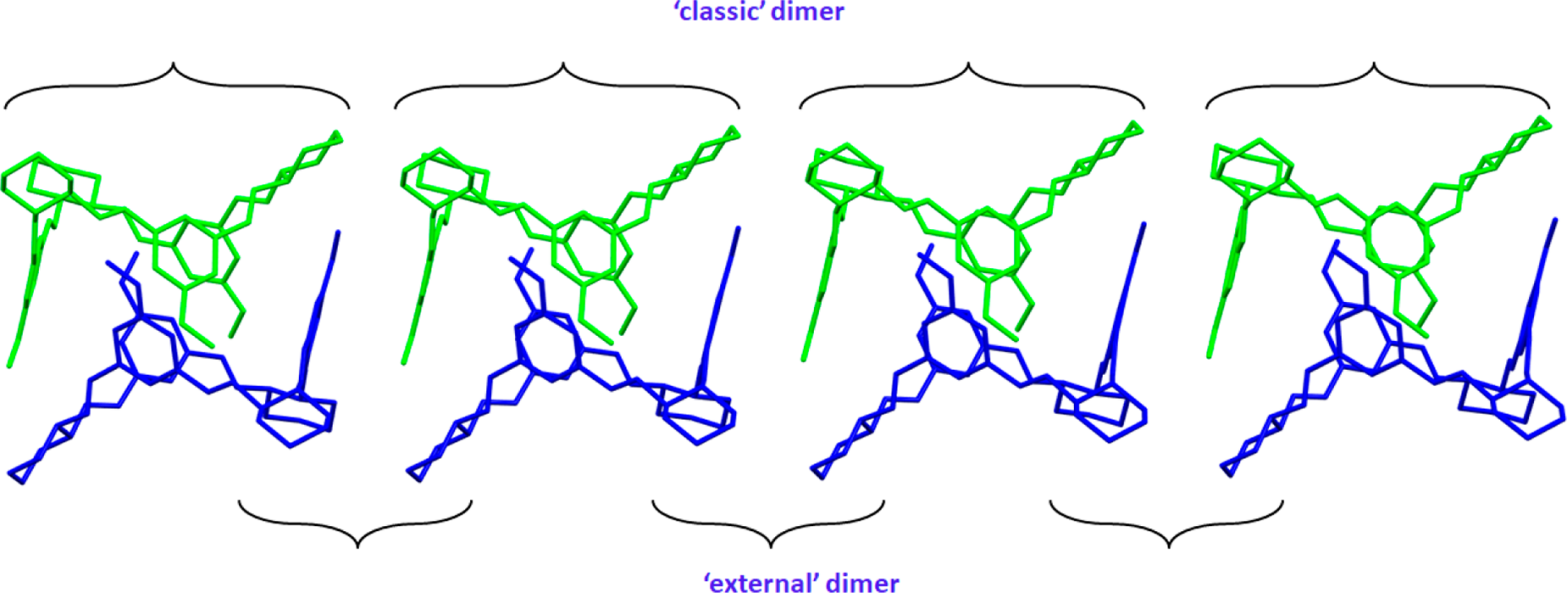
Alternate arrangements of two supramolecular dimers present in the crystals of **3c** and **3d**. Symmetry-independent macrocycles are shown in different colors. Hydrogen atoms have been omitted for clarity.

In **3c**, two macrocycles form the asymmetric unit and are arranged in the “classic” tail-to-tail motif; no π···π interactions were observed within this motif. The interacting phenyl rings are mutually offset in a linear fashion such that the oxygen atom of the methoxy substituent of one macrocycle is located just above the aromatic ring of a neighboring molecule. This arrangement favors (O)_lone-pair_···π interactions (*d*_O···π_ = 3.479(8) and 4.498(9) Å) over the classic π···π interactions, which were excluded due to unfavorable geometric parameters: the centroid-to-centroid distance of 4.563(7) Å and the ring offset by more than 2.7 Å. Propagation of the crystal structure of **3c** in the [101] direction reveals that neighboring pairs of “classic” dimers self-aggregate in a tail-to-tail fashion to generate “external” dimers. Each “external” dimer thus obtained is stabilized by C–H···π interactions that occur between a methyl unit of the methoxy group and an aromatic ring and have an H···π distance of 2.85 A. The C–H···π interactions are supported by lone-pair···π interactions with short O···π contacts of 3.55(1) Å. As a result, the expanded framework along the [101] lattice direction is constructed from alternately arranged “classic” and “external” dimers that form pillars, as shown in Figure 5.

Stabilized by different types of interactions, similar supramolecular motifs are also present in the crystals of **3d**. Unlike those of **3c**, “classic” dimers of **3d** are stabilized mostly through π···π stacking interactions with a centroid-to-centroid distance of 3.730(2) Å and the ring offset of 1.51(1) Å. This motif, due to the presence of nitro groups, is strongly supported by multiple C–H···O, C–H···π, O···π, and (O)_lone-pair_···π interactions (mean *d*_H···O_ = 2.62 Å, mean *d*_H···π_ = 2.89 Å, mean *d*_O···π_ = 2.997(1) Å, and mean *d*(O)_l.p._···π = 3.106(1) Å, respectively) for which the contact lengths are shorter than the sum of the van der Waals radii of atoms involved in these interactions (sum of van der Waals radii for O and H is 2.72 Å, that for H and C is 2.9 Å, and that for O and C is 3.22 Å).^66^ The “classic” dimers aggregate themselves into “external” dimers along the [100] direction. Within these aggregates, two molecules are held together by π···π interactions that are characterized by short centroid-to-centroid distances (3.447(2) Å) and perfectly overlapping aromatic rings. In addition, π···π interactions in this motif are supported by C–H···O interactions, where the mean H···O distance is 2.69 Å and the mean C–H···O angle is 146°. As with **3c**, the further expansion of the structure also leads to the alternate arrangement of two supramolecular dimers in **3d**, the options for which are shown in Figure 5.

**Figure 6:**
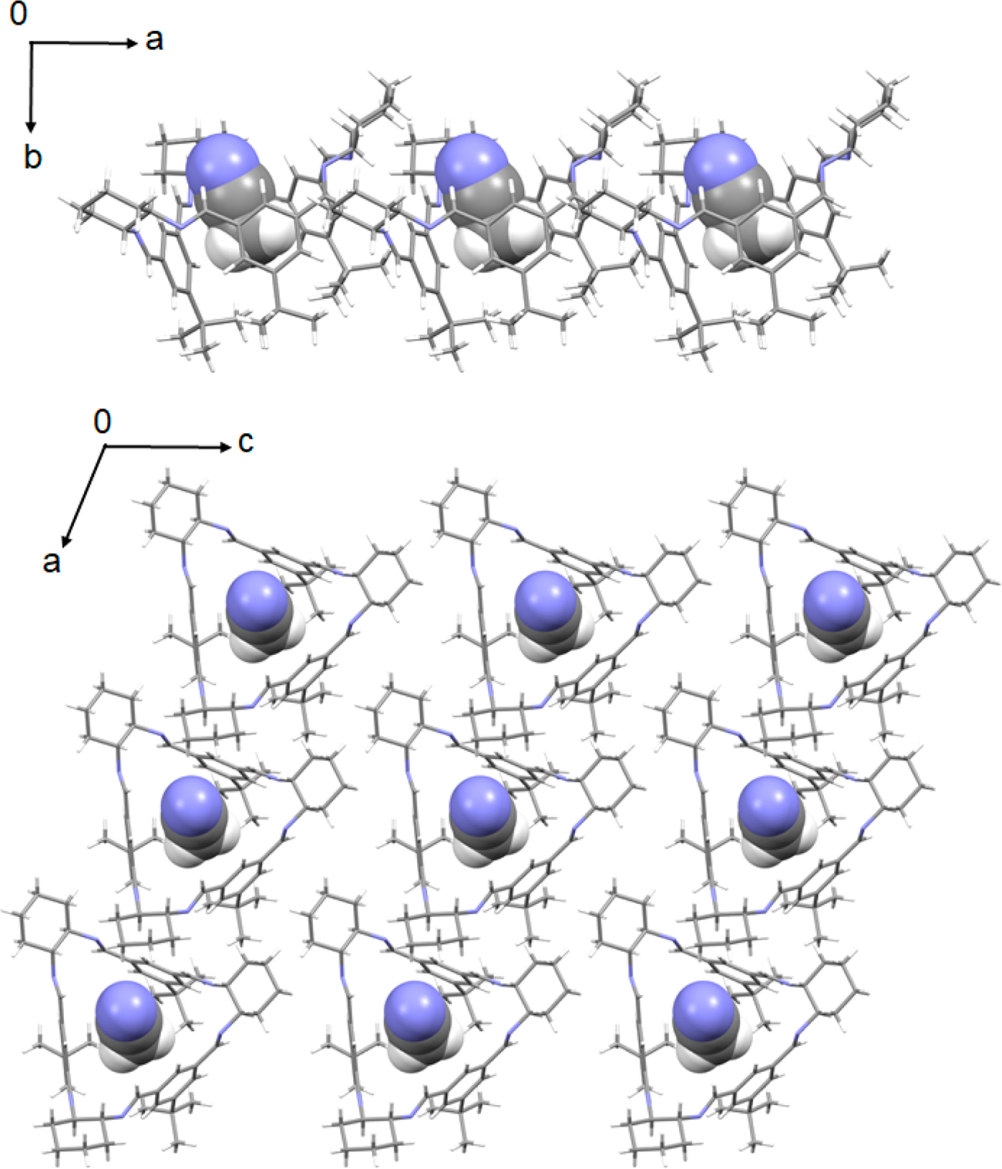
Side (above) and top (below) views of the layers in **3e**. Acetonitrile molecules accommodated in the molecular voids are represented as space-filling models.

Bulky *tert*-butyl substituents attached to the aromatic part of the lower rim usually prevent interdigitation by limiting access to the inner cavity. As we observed in the crystals of enantiomerically pure calixsalen substituted by *tert*-butyl groups, the formation of capsules is favored. On the other hand, racemic counterparts display a preference to self-organize into homochiral tail-to-tail dimers and heterochiral head-to-head capsules.^51−55^ In contrast, isotrianglimine **3e** does not show a preference to self-organize into either a tail-to-tail or head-to-head arrangement. Instead, it forms layers. The layers are perpendicular to the [010] direction and are composed of macrocycles with the same orientation (see Figure 6). The layer thickness is approximately 12.6 Å. Admittedly another layer is constructed in the same manner, but its orientation is different; it is twisted in relation to the previous one by approximately 78° and translated in such a way that none of the molecular fragments belonging to the macrocycles in the layer above obscure the macrocyclic cavities in the layer below. This isotrianglimine packing arrangement allows acetonitrile molecules to occupy each molecular cavity within the layer.

### 2.3 Quantum Chemical Calculations

Theoretical calculations done parallel to experimental studies would have provided not only structures of the neutral monomeric species and their respective assemblies but have also allowed for the estimation of interaction energies between respective assembly forming monomers. Due to the size of the molecules and their assemblies, we have employed the B3LYP hybrid functional for geometry optimization together with and empirical GD3BJ correction for dispersion and the triple-ζ basis set 6-311G(d,p).^67−73^ In the case of molecular complexes, the counterpoise correction in the scheme proposed by Boys and Bernardi has been utilized in a precise calculation of interaction energies.^74^

**Figure 7:**
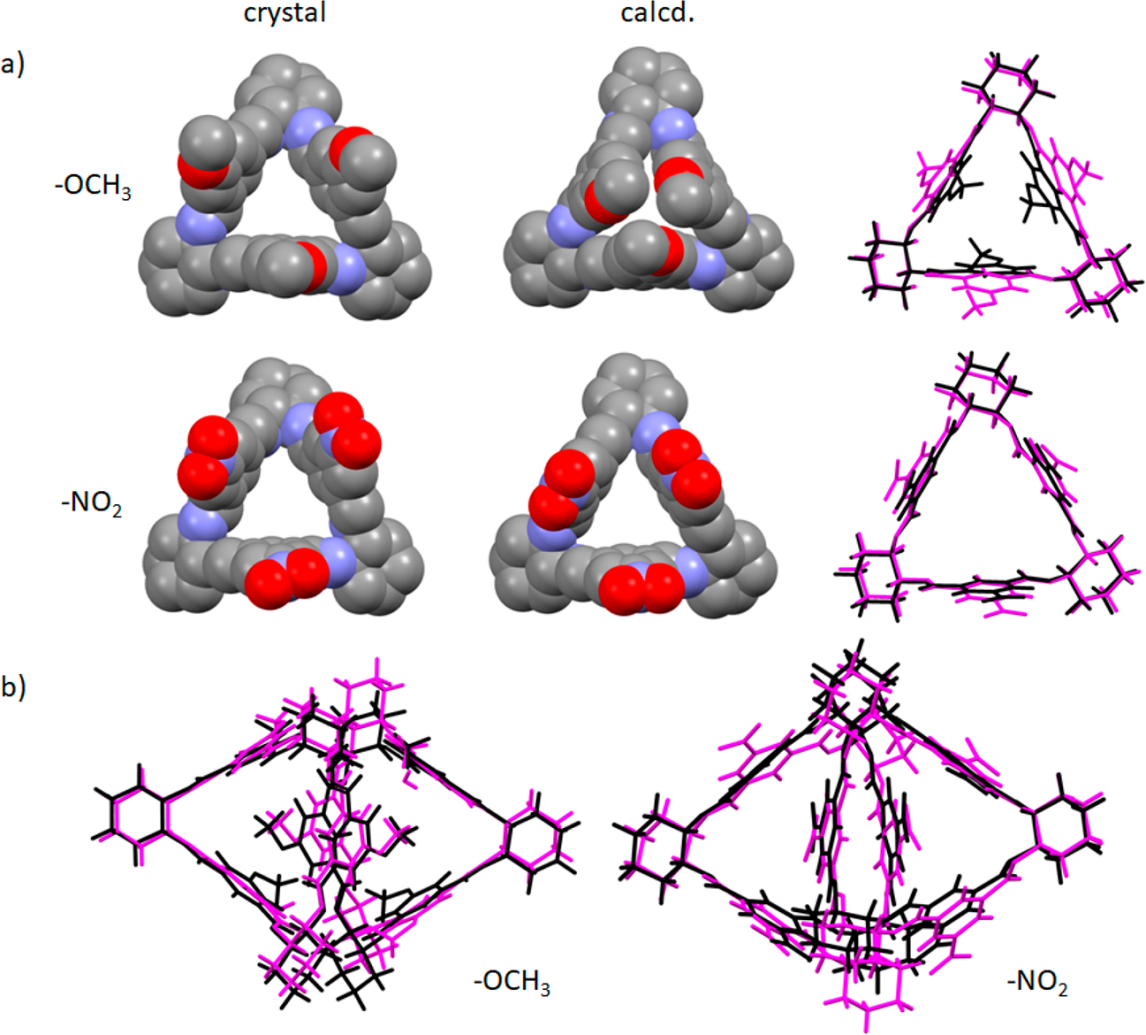
(a) The space-filling representation of experimentally determined and calculated structures of **3c** and **3d** and the overlay of the X-ray determined (pink) and calculated (black) structures. Some hydrogen atoms were omitted for clarity. (b) The overlay of X-ray determined (pink) and calculated (black) structures of isotrianglimine **3c** and **3d** dimers.

The comparison between example structures of isolated (calculated) isotrianglimines and those found in the crystals is shown in Figure 7.

A good match between experiment and theory was achieved only for **3d**. In the remaining cases, the macrocycle cavity is either obscured by aromatic fragments or fully opened. In the former case, especially for *tert*-butyl-substituted isotrianglimine **3e**, the steric repulsion between bulky alkyl fragments forced the aromatic rings to split outward. In the nonsubstituted **3a** and isotrianglimines substituted by electronegative atoms (**3b** and **3c**), the aromatic rings are facing inward, closing the cavity. However, these findings need to be taken with a certain amount of criticism. Structures of different origins were compared. The “experimental” structures were the parts of supramolecular assemblies; for that reason, their conformations are the resultant of intra- and intermolecular interactions, with the dominance of the latter. For the optimized in vacuo monomeric **3a**–**3e**, only intramolecular interactions determine the structure of the given species.

The direct comparison between structures of respective assemblies is more valuable. Indeed, in most cases the experimentally determined structures and those calculated show a high degree of similarity (see Table 1). Significant deviations are visible when calculated structures are compared with the experimentally determined ones that contain entrapped solvent molecules.

**Table 1:** Average Diameter of the Macrocycle Cavity (Å)

isotrianglimine		crystal	dimer (calcd.)^a^	monomer (calcd.)^a^
**3a**_**3**	mol1	7.319	6.708	6.181
	mol2	7.409	6.792	
**3b**_**1**	mol1	6.897	6.897	5.276
	mol2	6.992	6.897	
**3b**_**2**	mol1	7.224	6.897	5.276
	mol2	7.189	6.897	
**3c**	mol1	6.652	6.705	5.271
	mol2	6.698	6.705	
**3d**	mol1	6.945	6.746	6.389
	mol2	7.091	6.746	
**3e**		6.073	–b	6.991

a Calculated at the B3LYP-GD3BJ/6-311G(d,p) level.

b Tail-to-tail dimers have not been found in the solid-state.

**Figure 8:**
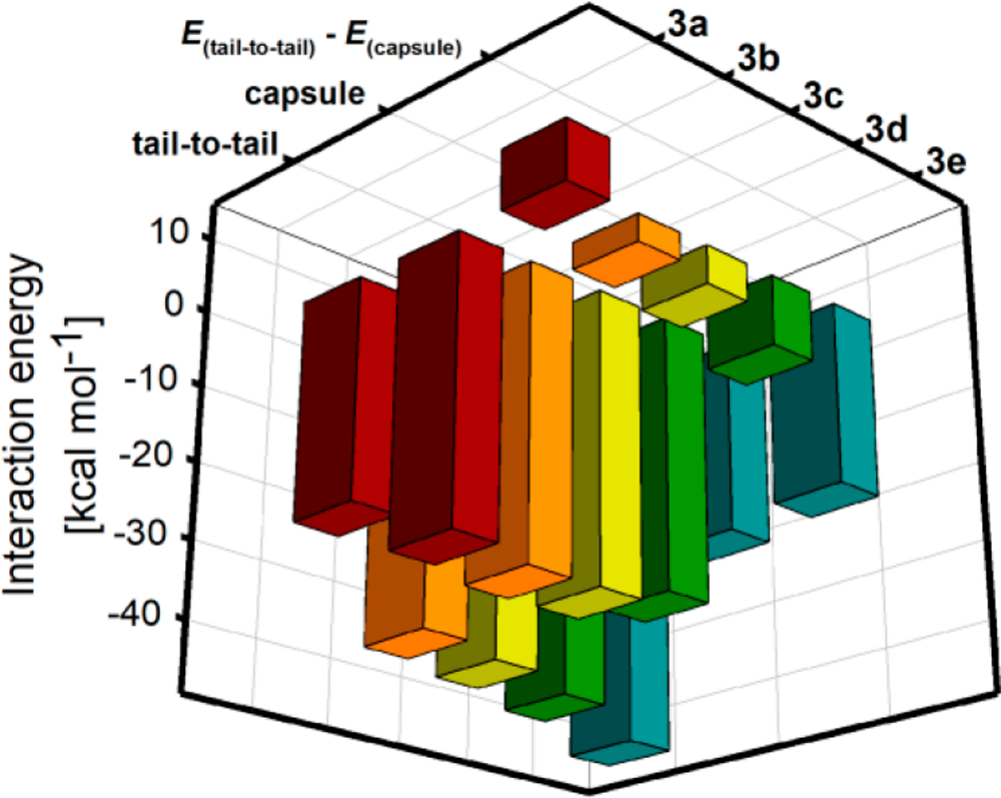
Interaction energies between **3a**–**3e** monomers forming respective tail-to-tail and capsule-type dimers calculated at the B3LYP-GD3BJ/6-311G(d,p) level and the difference in interaction energies between tail-to-tail and capsule-type dimers (Δ*E* = *E*_tail-to-tail_ – *E*_capsule_).

Except for **3a**, the energy preference for the formation of tail-to-tail dimers over the head-to-head dimers is visible (see Figure 8). The preference remained in agreement with experimental data for isotrianglimines **3a−3d**. In the case of isotrianglimine **3e**, the tail-to-tail dimeric forms were not found in the solid-state. Surprisingly, the strongest preference toward the interdigitating dimeric form was noticed for **3e**. While dimers of **3b**–**3d** are stabilized mostly by π···π, heteroatom···π, and dipole–dipole interactions, the extraordinarily stable dimer of **3e** is stabilized primarily by multiple C_sp^3^_–H···π interactions. For the in silico structure of **3e**, the energy gain resulting from these interactions prevails over the energy increase due to the deformation of the macrocycle. On the other hand, a lack of dipole–dipole and heteroatom···π interactions in the tail-to-tail dimer of **3a** constitutes the main reason why in this particular case the interdigitation does not bring energy profit. For all calculated tail-to-tail structures, the dimerization energy values for **3a**–**3e** diminish in the order **3a** > **3b** > **3c** > **3d** > **3e**. Unlike the tail-to-tail dimers, the capsules (head-to-head or window-to-window dimers) do not use both aromatic ring electron cloud interactions or heteroatom···π interactions to stabilize the associates. Instead, the interactions between aliphatic and aromatic parts of the dimer-forming molecules are visible. However, the tendency to maximize the C_sp^3^_–H···π interactions between axial protons from cyclohexane rings and aromatic π-electron clouds caused the significant deformation of the structures of individual monomers in the capsules. It is worth noting that except for **3e** the kind of substituent attached to the aromatic rings has only a small effect on calculated dimerization energies.

### 2.4 Self-Sorting Study

The comparison of the lattice parameters and the analysis of the packing mode of isotrianglimines in the crystals of **3c** and **3d** have shown that these structures are isostructural. Therefore, we questioned whether it was possible to replace the methoxy group in the isotrianglimine skeleton with a nitro group or vice versa and what, if any, the structural consequences of this modification would be.

We have repeated the synthesis using equimolar amounts of dialdehydes **2c** (0.5 equiv) and **2d** (0.5 equiv) and doubled the amount of (*R*,*R*)-**1** (1 equiv). The initial reaction was run for 12 h at room temperature in dichloromethane as the solvent. The concentration of substrates was kept at 0.02 and 0.01 M for **1** and the aldehydes, respectively. We observed the formation of a white precipitate during the reaction, which in turn affected the equilibria between possible products. However, repeating of this reaction in conditions that ensured solubility of all ingredients led to conclusion that in such a particular case the formation of mixed macrocycles containing two of the same aldehyde units and one unit of the second aldehyde is equally possible compared to the formation of “pure” **3c** and **3d** (see Figure S30). ESI-TOF mass spectra further confirmed the formation of the mixture of macrocycles.

The mixture of products was then crystallized at room temperature from a mixture of chloroform and acetonitrile by the slow evaporation of the solvents. We obtained single crystals that were subjected to single-crystal X-ray structure analysis. This, in turn, revealed that each of the studied crystals contained only one type of isotrianglimine, either **3c** or **3d**, of the same crystalline phase as those described above in this article.

## 3 Conclusion

In these studies, we have drawn our attention to the possibility of forming various supramolecular architectures using isotrianglimines that have substituents with different electronic and structural properties at the upper rim of the macrocycle. An additional premise for dealing with this group of compounds was the presence of the isotrianglimine moieties in one of the most intensely studied polyimine cages (see Scheme 1c),^37−40^ which points to the particular importance of this structural fragment in building more complex systems.

For most of the cases analyzed here, a propensity for forming tail-to-tail dimers by the mutual insertion of the aromatic rings of each molecule into the void of the other isotrianglimine is visible. The tail-to-tail dimers might further assemble into higher-order structures consisting of alternating tail-to-tail dimer and capsule motifs. Such an association pattern has been demonstrated for the first time for an optically pure triangular and vase-like macrocycle. Although it is theoretically possible, the presence of bulky *tert*-butyl groups in the molecule of **3e** prevented interdigitation in the solid-state. The formation of the window-to-window (the capsule) dimer was also not observed in the crystal. Instead, in the crystal this particular macrocycle is associated with layers mutually twisted by 78°. As a result of this mode of association, each macrocycle can serve as a container for one solvent molecule.

Surprisingly, nonsubstituted **3a** formed solid-state superstructures that resembled a honeycomb. The detailed mode of the organization of the molecules in the crystal lattice is affected by the presence of the molecule. Note that the presence of isomeric solvents that do not differ much in their structural and chemical properties may significantly change the mode of association in the solid state.

Structurally similar to calixsalens, the [3 + 3] isotrianglimines cannot be fully treated as calixsalens without OH groups. The differences are evident on many levels starting from synthesis, through optical properties, and ending with the way of association. Contrary to isotrianglimines, calixsalens can be obtained only as triangular [3 + 3] products. The lack of OH groups caused the synthesis of isotrianglimines to be less predictable, especially at elevated temperatures. Chiroptical properties of isotrianglimines (discussed in detail in the SI) are different from those found for calixsalens in terms of the electronic transition energies, rotatory strengths, and amplitudes of Cotton effects. Contrary to calixsalens, the polarity of the solvent and the nature of the substituent at the C5 position of an aromatic ring have negligible effects on the ECD spectra of isotrianglimines. The 280–220 sequence of exciton Cotton effects (CEs) observed at the spectral region is negative–positive and reflects both the negative helicity of (*R*,*R*)-**1** and the *C*_3_ symmetry of a triple chromophoric structure of the given isotrianglimine macrocycle (see Figure S12 in the SI).

Finally, although the association mode of isotrianglimines and calixsalens seems to be the same, the origin of the interactions that bind the superstructure is different. In the case of calixsalens, the interdigitating dimers are stabilized primarily by π···π and dipole···dipole interactions between aromatic rings. For isotrianglimines, these latter interactions are rather exceptional, and dimers are stabilized by weaker interactions, including those with solvent molecules. The (super)structure-forming role of solvent molecules is a feature of the isotrianglimine association mode.

The possibility of separating a mixture of two isotrianglimines by crystallization is worth noting. Such a process has not been reported previously for any triangular-shaped macrocycles.

Isotrianglimines, although not easy “in collaboration” as calixsalens, are characterized by their not fully explored potential in crystal engineering. The propensity of these compounds to form hybrid materials consisting of molecules differing in their absolute configuration (the heterochiral pairing strategy proposed Cooper)^56^ or substitution pattern (formation of cocrystals or solid solutions) is especially interesting. Although the results presented here do not confirm the last hypothesis, the work on this current topic is in progress.

## Supporting Information Available

Detailed procedures for the syntheses of all compounds; characterizations in gas, solution, and solid states; and computational methods, including Cartesian coordinates (PDF)

## Accession Codes

CCDC 2096600–2096607 contain the supplementary crystallographic data for this paper. These data can be obtained free of charge via www.ccdc.cam.ac.uk/data_request/cif, or by emailing data_request@ccdc.cam.ac.uk, or by contacting The Cambridge Crystallographic Data Centre, 12 Union Road, Cambridge CB2 1EZ, UK; fax: +44 1223 336033.

## Dedication

Dedicated to Professor Bernard Lucas Feringa on the occasion of his 70th birthday.
