# Supplementary material for: Specific Noncovalent Association of Truncated exo-Functionalized Triangular Homochiral Isotrianglimines through Head-to-Head, Tail-to-Tail, and Honeycomb Supramolecular Motifs
Source: J Org Chem. 2022 Jan 14;87(5):2356–66. doi: 10.1021/acs.joc.1c02238 (PMC8902749; doi:10.1021/acs.joc.1c02238)
Supplement: Supplementary file 1 — jo1c02238_si_001.pdf [file jo1c02238_si_001.pdf]

Supporting Information

for

Specific non-covalent association of truncated, exo-functionalized triangular homochiral isotrianglimines through head-to-head, tail-to-tail, and honeycomb supramolecular motifs

Agnieszka Janiak,[†] Jadwiga Gajewy,[†] Joanna Szymkowiak,[† ‡] Błażej Gierczyk,[†] Marcin Kwit*[,†]*

[†]Faculty of Chemistry, Adam Mickiewicz University, Uniwersytetu Poznańskiego 8, 61-614 Poznań, Poland

[‡]present address: Faculty of Science, Department of Chemistry, University of British Columbia,

2036 Main Mall, Vancouver, British Columbia, BC Canada V6T 1Z1

Correspondence email: agnieszk@amu.edu.pl or marcin.kwit@amu.edu.pl

Table of Contents

1. Experimental details ....SI 5

2. Synthesis ....SI 6

3. Computational details....................SI 8

4. Chiroptical properties ....SI 9

Table S1. Total energies (in Hartree) estimated interaction energies with (IE_{CP}, in kcal mol^{-1}) and without counterpoise correction (IE, in kcal mol^{-1}) and number of imaginary frequencies (#ImFreq) calculated for respective monomers (m), tail-to-tail (t-to-t) and head-to-head (h-to-h, capsule) dimers of isotrianglimines 3a-3e, at the B3LYP-GD3BJ/6-311G(d,p) level of theory....................SI 11

Table S2. Specific, concentration-dependent optical rotations for isotrianglimines 3b-3e measured in chloroform solution and calculated at the IEFPCM/B3LYP/6-311G(d,p) level of theory for respective monomers, tail-to-tail (t-to-t) and head-to-head (h-to-h, capsule) dimers of isotrianglimines 3b-3e.SI 12

Figure S1. Parts of the ^{1}H NMR spectra (all spectra were measured in $CDCl_3$, 300 MHz, 20 °C) recorded during a cycloimination reaction between (*R*,*R*)-1 and 2c....................SI 13

Figure S2. Parts of the ^{1}H NMR spectra (all spectra were measured in $CDCl_3$, 300 MHz, 20 °C) recorded during a cycloimination reaction between (*R*,*R*)-1 and 2d. ....SI 14

Figure S3. The ^{1}H NMR spectra recorded at certain time intervals for the solution of isotrianglimine 3b (all spectra were measured in $CDCl_3$, 300 MHz, 20 °C)....................SI 15

Figure S4. The ^{1}H NMR spectra recorded at certain time intervals for the solution of isotrianglimine 3c (all spectra were measured in $CDCl_3$, 300 MHz, 20 °C)....................SI 16

Figure S5. The ^{1}H NMR spectra recorded at certain time intervals for the solution of isotrianglimine 3d (all spectra were measured in $CDCl_3$, 300 MHz, 20 °C)....................SI 17

Figure S6. The ^{1}H NMR spectra recorded at certain time intervals for the solution of isotrianglimine 3e (all spectra were measured in $CDCl_3$, 300 MHz, 20 °C)....................SI 18

Figure S7. Structures of low-energy a) monomer, b) tail-to-tail and c) head-to-head (capsule) dimers of isotrianglimine 3a, calculated at the IEFPCM/B3LYP-GD3BJ/6-311G(d,p) level of theory. ....SI 19

Figure S8. Structures of low-energy a) monomer, b) tail-to-tail and c) head-to-head (capsule) dimers of isotrianglimine 3b, calculated at the IEFPCM/B3LYP-GD3BJ/6-311G(d,p) level of theory. ....SI 20

Figure S9. Structures of the lowest energy a) monomer, b) tail-to-tail and c) head-to-head (capsule) dimers of isotrianglimine 3c, calculated at the IEFPCM/B3LYP-GD3BJ/6-311G(d,p) level of theory.SI 21

Figure S10. Structures of low-energy a) monomer, b) tail-to-tail and c) head-to-head (capsule) dimers of isotrianglimine 3d, calculated at the IEFPCM/B3LYP-GD3BJ/6-311G(d,p) level of theory. ....SI 22

Figure S11. Structures of low-energy a) monomer, b) tail-to-tail and c) head-to-head (capsule) dimers of isotrianglimine 3e, calculated at the IEFPCM/B3LYP-GD3BJ/6-311G(d,p) level of theory. ....SI 23

Figure S12. UV (upper panels) and ECD (lower panels) spectra of isotrianglimines 3a-3e measured in chloroform solutions. ....SI 24

Figure S13. UV (upper panels) and ECD (lower panels) spectra of isotrianglimines a) 3c and b) 3d measured in chloroform solutions (black lines) and calculated for respective monomers (red lines), tail-to-tail dimers (blue lines) and capsules (green lines) of isotrianglimines 3c and 3d at the IEFPCM/M06-2X/6-311G(d,p) level. The wavelengths have been corrected to match experimental UV maxima. ....SI 25

5. Single-Crystal X-ray diffraction ....SI 26

Table S3. Crystal data and structure refinement for 3a-3e. ....SI 27

Table S4. The estimated size of the macrocyclic cavity in the investigated crystals. ....SI 28

Figure S14. A perspective view of a) 3a_1 and b) 3a_2. Labels mark the symmetry independent part of molecules. Ellipsoids are drawn at the 40% probability level, hydrogen atoms are represented by spheres of arbitrary radii. ....SI 29

Figure S15. A packing diagram of macrocycles in the crystals of 3a_1 and 3a_2 as viewed along the *c* lattice direction. The host molecules are shown in space-filling style. ....SI 30

Figure S16. A packing diagram of 3a_1 showing the void (in green) from which the solvent molecules were removed. The view is along the *c* lattice direction. ....SI 30

Figure S17. An illustration of asymmetric unit of 3a_3. For clarity only the heteroatoms were labeled. Ellipsoids are drawn at the 40% probability level, hydrogen atoms are represented by spheres of arbitrary radii. ....SI 31

Figure S18. A packing diagram of 3a_3 as viewed along the *a* lattice direction. The two symmetry independent host molecules are shown in green and blue whereas *m*-xylene molecules are shown in red ....SI 32

Figure S19. a) Top and b) side view of the internal space in 3a_3. The host molecules are shown in stick style whereas the guest molecules are shown as space-filling models. The solvent accessible volume of 84 $Å^3$ is shown as pink Connolly surfaces[21] using a probe radius of 1.5 Å. ....SI 33

Figure S20. An illustration of asymmetric unit of 3b_1. For clarity only the heteroatoms were labeled. Ellipsoids are drawn at the 40% probability level, hydrogen atoms are represented by spheres of arbitrary radii. Two symmetry independent macrocyclic molecules form interdigitated dimeric motif. ....SI 34

Figure S21. A packing diagram of 3b_1 as viewed along the *a* lattice direction. The two symmetry independent host molecules are shown in green and blue in stick style. ....SI 34

Figure S22. a) A packing diagram of 3b_2 as viewed along the *a* lattice direction. b) The column consists of two symmetry independent host molecules that form capsules and interdigitated dimeric motifs. These motifs are shown as space-filling model. All H atoms were omitted for clarity. ....SI 35

Figure S23. An illustration of asymmetric unit of 3c. For clarity only the heteroatoms were labeled. Ellipsoids are drawn at the 40% probability level, hydrogen atoms are represented by spheres of arbitrary radii. ....SI 36

Figure S24. A packing diagram of 3c as viewed along the *a* lattice direction. The two symmetry independent host molecules are shown in green and blue in stick style whereas water molecules are shown as space-filling model. ....SI 37

Figure S25. Asymmetric unit of 3d. For clarity only the heteroatoms were labeled. Ellipsoids are drawn at the 40% probability level, hydrogen atoms are represented by spheres of arbitrary radii. Two symmetry independent macrocyclic molecules form interdigitated dimeric motif. ....SI 38

Figure S26. A packing diagram of 3d as viewed along the *a* lattice direction. The two symmetry independent host molecules are shown in green and blue in stick style. ....SI 38

Figure S27. Asymmetric unit cell of 3e contains a molecule of isotrianglimine and three molecules of acetonitrile. For clarity only the heteroatoms were labeled. Ellipsoids are drawn at the 40% probability level, hydrogen atoms are represented by spheres of arbitrary radii............................SI 39

Figure S28. A packing diagram of 3e as viewed along the *a* lattice direction. The host molecules are shown in capped sticks style whereas guest molecules are shown as space-filling model. The colors represent symmetry independent acetonitrile molecules. ....SI 40

Figure S29. Definition of the method of calculating the size of the macrocyclic cavity. ....SI 41

Figure S30. The ^{1}H NMR spectrum recorded for the crude reaction mixture obtained in reaction between equimolar amounts of dialdehydes 2c (0.5 eq) and 2d (0.5 eq) and doubled the amount of (*R*,*R*)-1 ($CDCl_3$, 300 MHz, 20 °C). ....SI 42

6. Cartesian coordinates (IEFPCM/B3LYP-GD3BJ/6-311G(d,p) method) ....SI 43

7. References ....SI 100

1. Experimental details

The ^{1}H and $^{13}C\{1H\}$ NMR spectra were recorded on a BRUKER ASCEND™ 600 MHz or a Bruker Ultrashield 300 MHz instruments. Chemical shifts (δ) are reported in ppm relative to $SiMe_4$ or trace solvent signals. Mass spectra were recorded on an AB SciexTripleTOF® 5600+ System. IR spectra were recorded on a Thermo Scientific Nicolet iS50 FT-IR spectrometer and reported as wave numbers ν in cm^{-1}. A Jasco P-2000 polarimeter was used for optical rotation measurements (at 20 °C, $CHCl_3$). For isotrianglimines **3b**-**3e**, the optical rotations were measured at four different wavelengths: 436, 546, 578 and 589 nm. The concentration of analytes ranged from 2.0 to 0.125 g 100 mL^{-1}.

ECD and UV spectra were measured using a Jasco J-810 spectropolarimeter at room temperature in dry and degassed chloroform solutions and with the use of a quartz cell of optical lengths 0.1 cm. The concentration of analytes ranged from 1.0 to 2.0×10^{-4} mol L^{-1}. Background spectra of the pure solvents were recorded from 600 to 220 nm with the scan speed of 100 nm min^{-1}. The ECD spectra of analytes were measured with 16 accumulations. Dilution of the samples up to 10^{-6} mol L^{-1} did not have any profound effect on the measured UV and ECD spectra.

Flash column chromatography was performed on Merck Kieselgel type 60 (250 - 400 mesh). Merck Kieselgel type $60F_{254}$ analytical plates were employed for TLC. Melting points were measured on a Büchi Melting Point B-545 and uncorrected.

2. Synthesis

All reagents were provided by commercial suppliers and were used as received.

All known compounds were identified by spectroscopic comparison with authentic samples. The isophthalaldehyde (**2a**) was purchased from Sigma-Aldrich and used as received. Aldehydes: 5-bromoisophthalaldehyde (**2b**), 5-methoxyisophthalaldehyde(**2c**), 5-*tert*-butylisophthalaldehyde (**2e**) were obtained from commercially available respective 5-substituted isophthalic acids by reduction to a corresponding diol and subsequent oxidation to dialdehyde.[1] 5-Nitroisophthalaldehyde (**2d**) was obtained by nitration of commercially available isophthaldehyde (**2a**) according to the previously published procedure.[1]

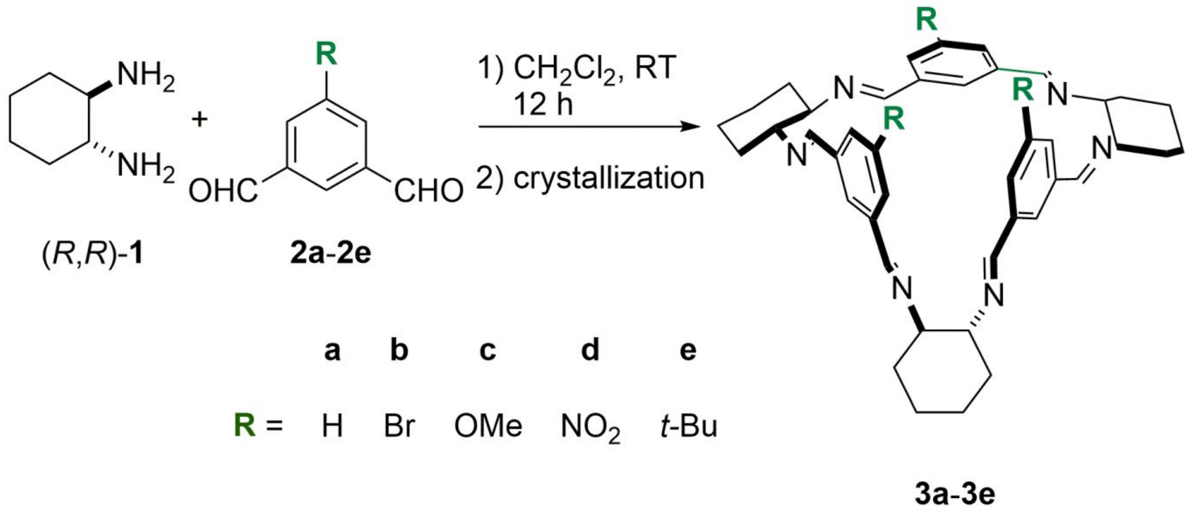

Scheme S1.

The general procedure for the synthesis of isotrianglimines 3a-3e (**Scheme S1**). To a stirring solution of *trans*-(1*R*,2*R*)-diaminecyclohexane (114 mg, 1 mmol) in 25 mL of dry dichloromethane, a solution of appropriate dialdehyde (1 mmol, 1 eq) in 5 mL of dry dichloromethane was added dropwise. The whole mixture became turbid within 30 minutes. The stirring was continued for additional 12 hours, in room temperature and under inert atmosphere. After evaporation of all volatiles under reduced pressure, the crude products were pure enough for the further use (isolated yields >99%). The analytically pure samples were obtained by dissolving the crude product in small amount of anhydrous dichloromethane and then anhydrous acetonitrile was added drop-wise. The pure macrocycles precipitated as white solids. Yields after precipitation procedure: **3a** – 189 mg (89%), **3b** – 201 mg (69%), **3c** – 172 mg (71%), **3d** – 218 mg (85%), **3e** – 142 mg (53%).

Isotrianglimines **3a**-**3e** gave all spectra identical to that previously published.[2]

Synthesis of isotrianglimines 3c and 3d at elevated temperature. To a stirring solution of *trans*-(1*R*,2*R*)-diaminecyclohexane (114 mg, 1 mmol) in 25 mL of dry chloroform, a solution of dialdehyde **2c** or **2d** (1 mmol, 1 eq each) in 5 mL of dry chloroform was added drop-wise. The whole mixture was heated to reflux for 48 hours using heating mantle. After evaporation of all volatiles under reduced pressure, the crude products (**3c** = 258 mg, 100% of yield, mixture of isomers; **3d** = 273 mg, 100% of yield, mixture of isomers) were analyzed by means of ^{1}H NMR spectroscopy. The reactions were repeated at prolongated time. However, after heating of the reaction mixtures to reflux (heat source: heating mantle) up to two weeks no substantial change in the NMR pictures have been noticed.

Synthesis of 'mixed' isotrianglimine. To a stirring solution of *trans*-(1*R*,2*R*)-diaminecyclohexane (114 mg, 1 mmol) in 75 mL of dry dichloromethane, a solution of dialdehyde **2c** and **2d** (0.5 mmol each, 0.5 eq each) in 10 mL of dry dichloromethane was added drop-wise. The stirring was continued for additional 12 hours, at room temperature and under inert atmosphere. After evaporation of all volatiles under reduced pressure, the crude product (264 mg, 100% of yield) as a mixture of macrocycles , was subjected for the further use.

NMR studies. The ^{1}H NMR spectra recorded at certain time intervals for the model reactions between (*R*,*R*)-**1** and **2c** and **2d**, carried out in $CDCl_3$ (Figures S1 and S2), gave an insight into the course of the cycloimination reactions. The test reactions have run in NMR tubes, in anhydrous $CDCl_3$ at room temperature and for 120 hours. Despite a different character of the substituent attached to the aromatic ring, both aldehydes are characterized by similar reactivity. Within 7 hours, most of the respective aldehyde disappeared and full conversion was achieved within 12 hours.

Stability tests. The ^{1}H NMR spectra recorded at certain time intervals for chloroform solution gave an insight into the stability of the isotrianglimines **3b**-**3e** in solution. A small portion (ca. 5 mg) of the crystals of a given trianglimine was dissolved in deuteriochloroform (0.7 mL, the solvent was used without special purification and drying). Then, the ^{1}H NMR spectra were recorded at certain time intervals for each of the solutions (see Figures S3-S6). The pure isotrianglimine **3b** has persisted in the extended form for at least 7 hours after dissolving in $CDCl_3$. On the contrary, for the alkyl-substituted **3e,** the first symptoms of a ring contraction were visible just after one hour. The isotrianglimine **3d** turned out to be the most resistant to the contraction of the ring and the equilibrium between [3 + 3] and [2 + 2] products was reached after 168 hours, whereas the methoxy-substituted **3c** reached equilibrium between cyclic products within 56 hours.

3. Computational details

Based on the molecular geometries in the crystals, we optimized the structures of monomers and dimers of **3a**–**3e** using DFT calculations, both *in a vacuo* and in a chloroform solution.

Starting geometries of monomeric macrocycles **3a**-**3e** with assumed conformations were obtained from crystallographic data and pre-optimized at the B3LYP-GD3BJ/6-31G(d) level.[3,4] The structures of respective monomers were further optimized *in a vacuo* and in a chloroform solution, using the polarizable continuum model (IEFPCM)[5] all with the use of B3LYP-GD3BJ functional in conjunction with the 6-311G(d,p) basis set.[3,4] The structures thus obtained were the real minimum energy conformers (no imaginary frequencies have been found). The same scheme was used in the cases of respective dimeric structures of **3a**-**3e**. We did not notice any differences between the structures optimized with and without the solvent model, therefore only the structures optimized with the IEFPCM solvent model are shown.

The counterpoise procedure was utilized in precise calculation of the interaction energies between monomers making up the dimers.[6] Calculated structures were shown in Figures S6-S11, total energies and estimated interaction energies with and without counterpoise correction were collected in Table S1.

Additionally, for all investigated compounds, ECD spectra were measured in chloroform solution and calculated at the IEFPCM/TDDFT/6-311G(d,p) level for all stable geometries, including monomers and dimers, according to the previously described procedure.[7] We employed four different hybrid functionals to calculate ECD spectra: M06-2X,[8] B2LYP,[9] LC-wPBE[10] and CAM-B3LYP.[11] Rotatory strengths were calculated using both length and velocity representations. In the present study, the differences between the length and velocity calculated values of rotatory strengths were quite small and, for this reason, only the velocity representations were further used. ECD spectra were simulated by overlapping Gaussian functions for each transition,[12] according to the procedure previously described.[7] It worth noting that all of employed functionals produced similar results, thus we restrict discussion to the results obtained with the use of M06-2X hybrid functional only.

The optical rotation values were calculated for **3b**-**3e** at the IEFPCM/B3LYP/6-311G(d,p) level.

All the above-mentioned calculations were performed with the use of Gaussian 09 package.[13]

In the case of isotrianglimine **3c**, rotation of methoxy groups has only a negligible effect on the calculated ECD spectra, thus, only the most stable conformer of **3c** is discussed.

4. Chiroptical properties

Measurements of ECD and ORD spectra were to have provided an alternative method to DOSY NMR measurements. We have reasoned that the direct comparison of experimental and calculated for respective monomeric/dimeric species ECD spectra would allow for the determination of preferred structure in the solution.

Absorption (UV) and electronic circular dichroism (ECD) spectra were measured in chloroform solution for all of the isotrianglimines discussed here. The UV and ECD spectra were shown in Figure S12. As it has been clearly seen, the ECD spectra of **3a**-**3e** showed a very high degree of similarity. Despite the nature of the substituents at C5 position of an aromatic ring, the sequence of strong Cotton effects (CEs) observed at the spectral region 280-220 is negative/positive. These CEs have formed exciton couplet[14] corresponding to the allowed electronic transitions of the diimine chromophores observed at around 240-230 nm and polarised along the axis connected the midpoints of the C=N imine bonds. The negative sign of these excitons have been determined by the negative helicity of (*R*,*R*)-DACH (the negative N–C*–C*–N torsion angle in the diaminocyclohexane moiety) and C_3 symmetry of a triple chromophoric structure of the macrocycle.

The change of solvent polarity from a mixture of chloroform and cyclohexane, through pure chloroform and up to a 50 : 50 mixture of chloroform and acetonitrile, has a negligible effect on the shape and intensity of the UV and ECD absorption bands.

As the representative example for further discussion, we have chosen the isotrianglimines **3c** and **3d**. Electronic spectra for all stable monomers and dimers of **3c** and **3d** were calculated at the IEFPCM/TD-DFT/6-311G(d,p) levels (see Computational details). As all the DFT methods produced similar results in terms of the shape of the spectrum and excitation energies, we have limited the discussion to the results obtained with the use of IEFPCM/TD-M06-2X/6-311G(d,p) method. The direct comparison of UV and ECD spectra measured for **3c** and **3d** with the theoretical ones, calculated for respective monomers and dimers of **3c** and **3d,** has been shown in Figure S13.

Comparison of the experimental UV and ECD spectra of **3c** and **3d** with the theoretical ones has not provided any unequivocal conclusion. Whereas for **3c** we might have excluded presence of monomeric form in the solution, the calculated ECD spectra for monomeric and dimeric form of **3d** did not differ much from each other. This remains in contrast to the previously published results for calixsalens, where calculated ECD spectra for respective forms were substantially different.[15] The almost perfect agreement between the experimental and calculated spectra for tail-to-tail dimeric species of calixsalens, seen especially in the higher-energy region, would allow for the unequivocal structural predictions.

In addition to ECD spectra, we have measured concentration-dependent ORD spectra for **3b**-**3e**. Due to the very low solubility in chloroform, we have not been able to do similar studies for the simplest isotrianglimine **3a**. In Table S2 we have juxtaposed the experimental data alongside with the results of ORD calculations (IEFPCM/B3LYP/6-311G(d,p) method) for respective monomers and dimers of **3b**-**3e**.

Gradual dilution of the samples from concentration 2 g mL^{-1} to 0.125 g mL^{-1} caused increase of measured absolute values of ORs at a given wavelength. This phenomenon was visible for all compounds under study and led to the conclusion that in such a concentration range, the equilibrium between monomers and dimers/oligomers was established. The calculation results seemed to suggest that the preferred form of the isotrianglimie in the solution was a tail-to-tail dimer. However, until it is possible to estimate the equilibrium constant and, thus, the mole fractions of the respective individuals, it is rather a speculation.

Table S1. Total energies (in Hartree) estimated interaction energies with (IE_{CP}, in kcal mol^{-1}) and without counterpoise correction (IE, in kcal mol^{-1}) and number of imaginary frequencies (#ImFreq) calculated for respective monomers (m), tail-to-tail (t-to-t) and head-to-head (h-to-h, capsule) dimers of isotrianglimines **3a**-**3e**, at the B3LYP-GD3BJ/6-311G(d,p) level of theory.

Compound	E	IE_{CP}	IE	#ImFreq
**3a** (m)	-1958.361262	-	-	0
**3a** (t-to-t)	-3916.705795	-28.78	-35.09	0
**3a** (h-to-h)	-3916.689603	-36.71	-43.67	0
**3b** (m)	-9678.978198	-	-	0
**3b** (t-to-t)	-19357.947997	-41.63	-48.14	0
**3b** (h-to-h)	-19357.936349	-38.08	-45.17	0
**3c** (m)	-2302.015328	-	-	0
**3c** (t-to-t)	-4604.0299963	-42.97	-53.95	0
**3c** (h-to-h)	-4604.011907	-37.97	-45.32	0
**3d** (m)	-2572.037003	-	-	0
**3d** (t-to-t)	-5144.051449	-44.88	-61.05	0
**3d** (h-to-h)	-5144.027780	-35.75	-42.54	0
**3e** (m)	-2430.255456	-	-	0
**3e** (t-to-t)	-4860.473222	-48.55	-55.44	0
**3e** (h-to-h)	-4860.425672	-25.03	-30.31	0

Table S2. Specific, concentration-dependent optical rotations for isotrianglimines **3b**-**3e** measured in chloroform solution and calculated at the IEFPCM/B3LYP/6-311G(d,p) level of theory for respective monomers, tail-to-tail (t-to-t) and head-to-head (h-to-h, capsule) dimers of isotrianglimines **3b**-**3e**.

	Concentration	Optical rotation			
	[g 100 mL^{-1}]	589 nm	578 nm	546 nm	436 nm
**3b**	2	-155	-164	-195	-425
	1	-167	-178	-212	-464
	0.5	-192	-206	-245	-540
	0.25	-217	-232	-278	-617
	0.125	-246	-267	-324	-729
Calcd. for:					
monomer		-732	-772	-911	-1942
t-to-t dimer		-261	-273	-312	-549
h-to-h dimer		-349	-368	-430	-866
**3c**	2	-266	-281	-336	-754
	1	-312	-332	-397	-903
	0.5	-381	-405	-486	-1108
	0.25	-450	-475	-476	-1307
	0.125	-441	-478	-579	-1344
Calcd. for:					
monomer		-854	-904	-1073	-2404
t-to-t dimer		-127	-132	-147	-162
h-to-h dimer		-424	-447	-525	-1094
**3d**	2	-198	-210	-249	-530
	1	-216	-228	-269	-577
	0.5	-246	-258	-309	-664
	0.25	-297	-321	-379	-814
	0.125	-308	-336	-397	-866
Calcd. for:					
monomer		-561	-593	-700	-1513
t-to-t dimer		-509	-539	-640	-1540
h-to-h dimer		-397	-419	-494	-1066
**3e**	2	-122	-129	-155	-355
	1	-133	-142	-170	-390
	0.5	-146	-157	-188	-433
	0.25	-174	-188	-227	-531
	0.125	-196	-220	-270	-636
Calcd. for:					
monomer		-350	-380	-447	-938
t-to-t dimer		92	98	118	271
h-to-h dimer		n.a.	n.a.	n.a.	n.a.

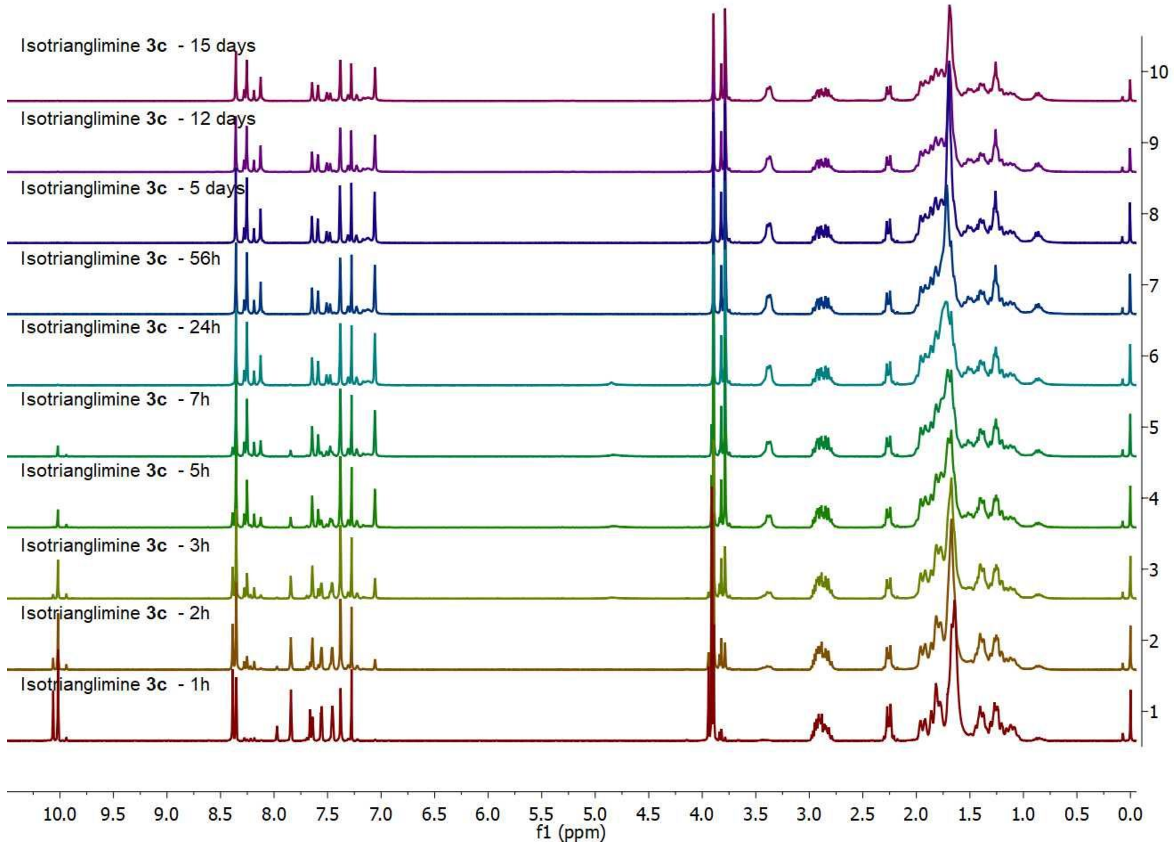

Figure S1. Parts of the ^{1}H NMR spectra (all spectra were measured in $CDCl_3$, 300 MHz, 20 °C) recorded during a cycloimination reaction between (*R*,*R*)-**1** and **2c**.

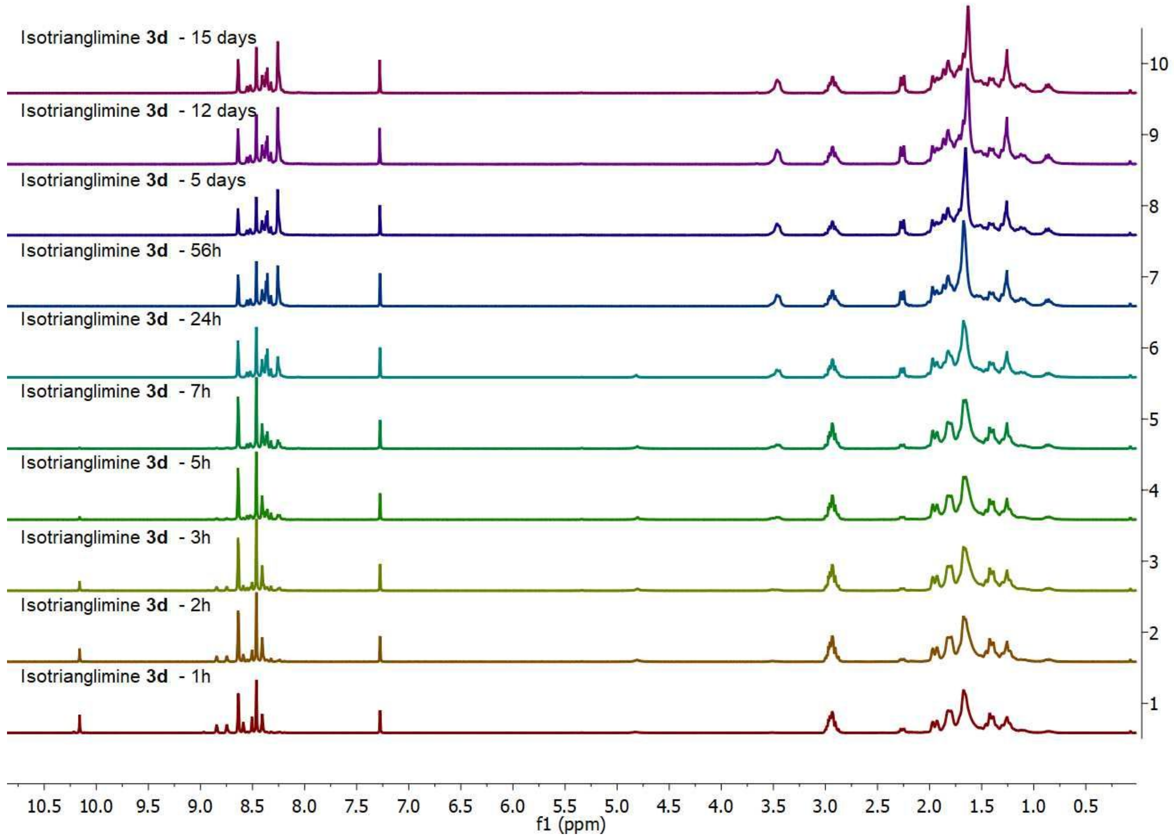

Figure S2. Parts of the ^1H NMR spectra (all spectra were measured in $CDCl_3$, 300 MHz, 20 °C) recorded during a cycloimination reaction between (*R*,*R*)-**1** and **2d**.

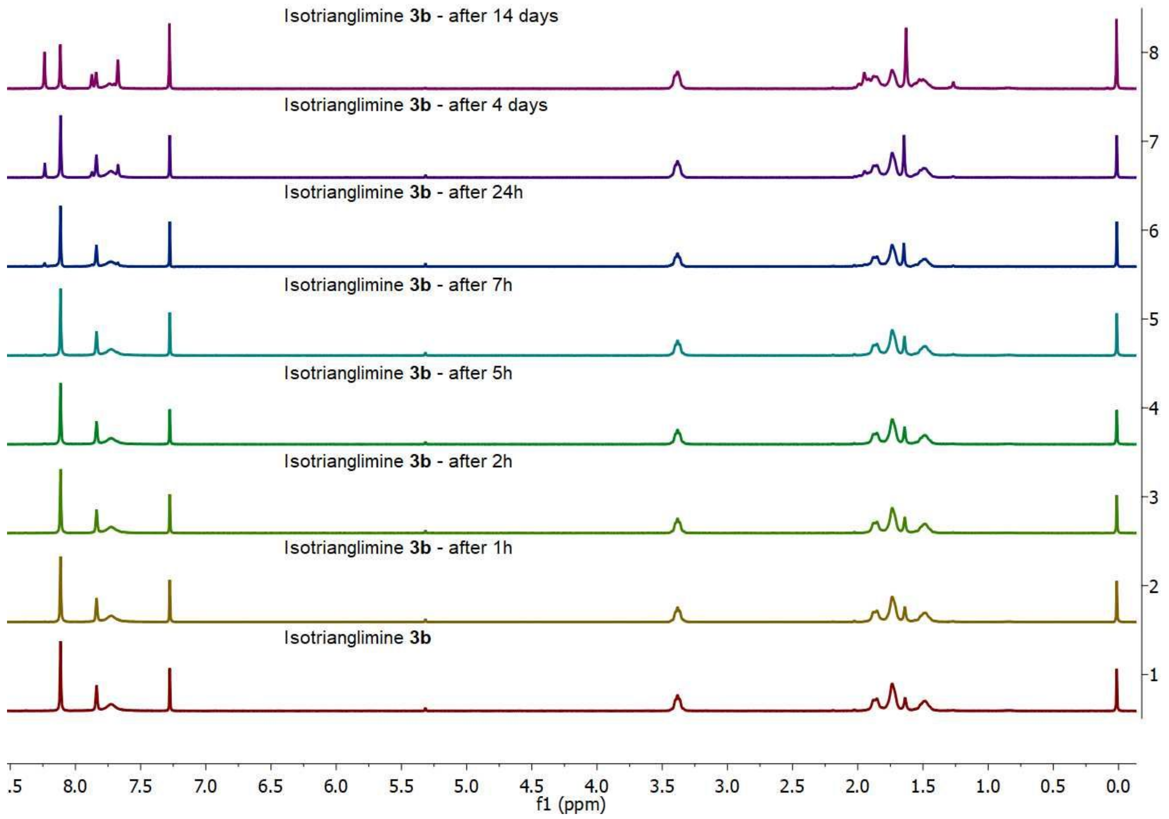

Figure S3. The ^{1}H NMR spectra recorded at certain time intervals for the solution of isotrianglimine **3b** (all spectra were measured in $CDCl_3$, 300 MHz, 20 °C).

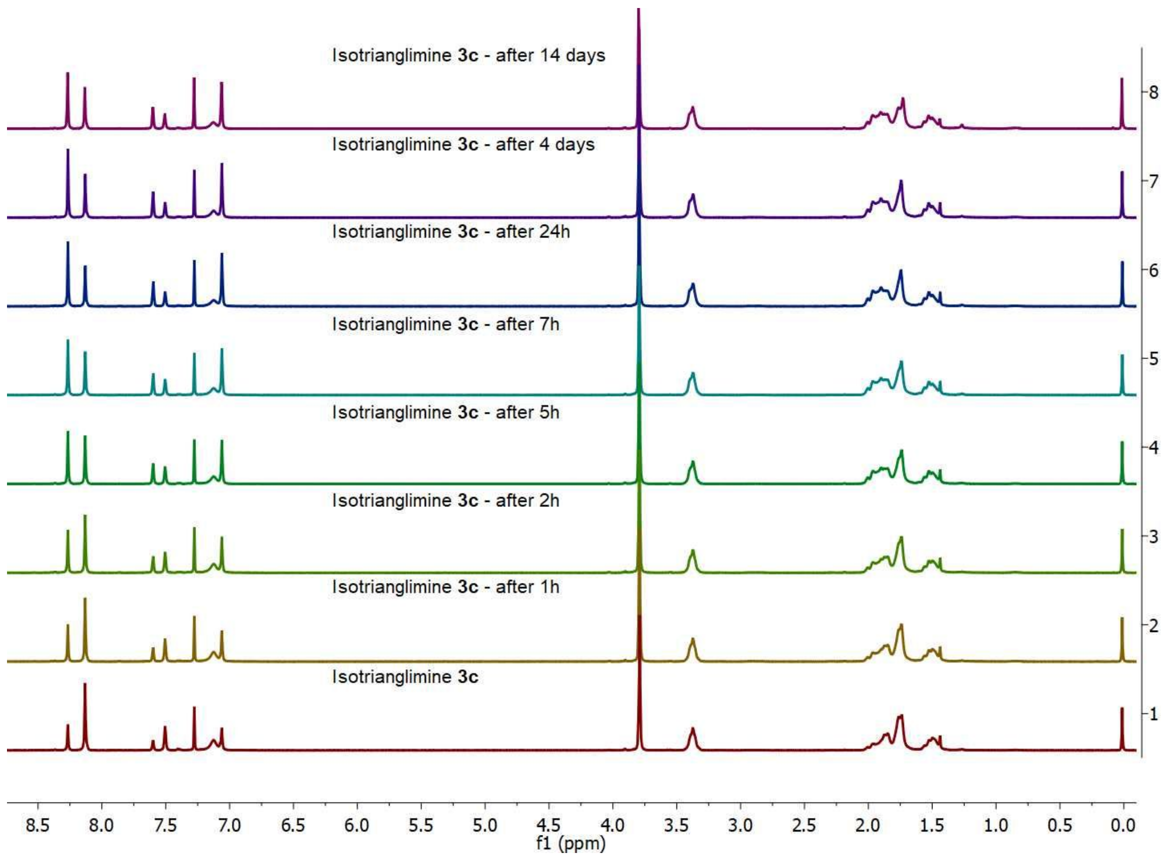

Figure S4. The ^{1}H NMR spectra recorded at certain time intervals for the solution of isotrianglimine **3c** (all spectra were measured in $CDCl_3$, 300 MHz, 20 °C).

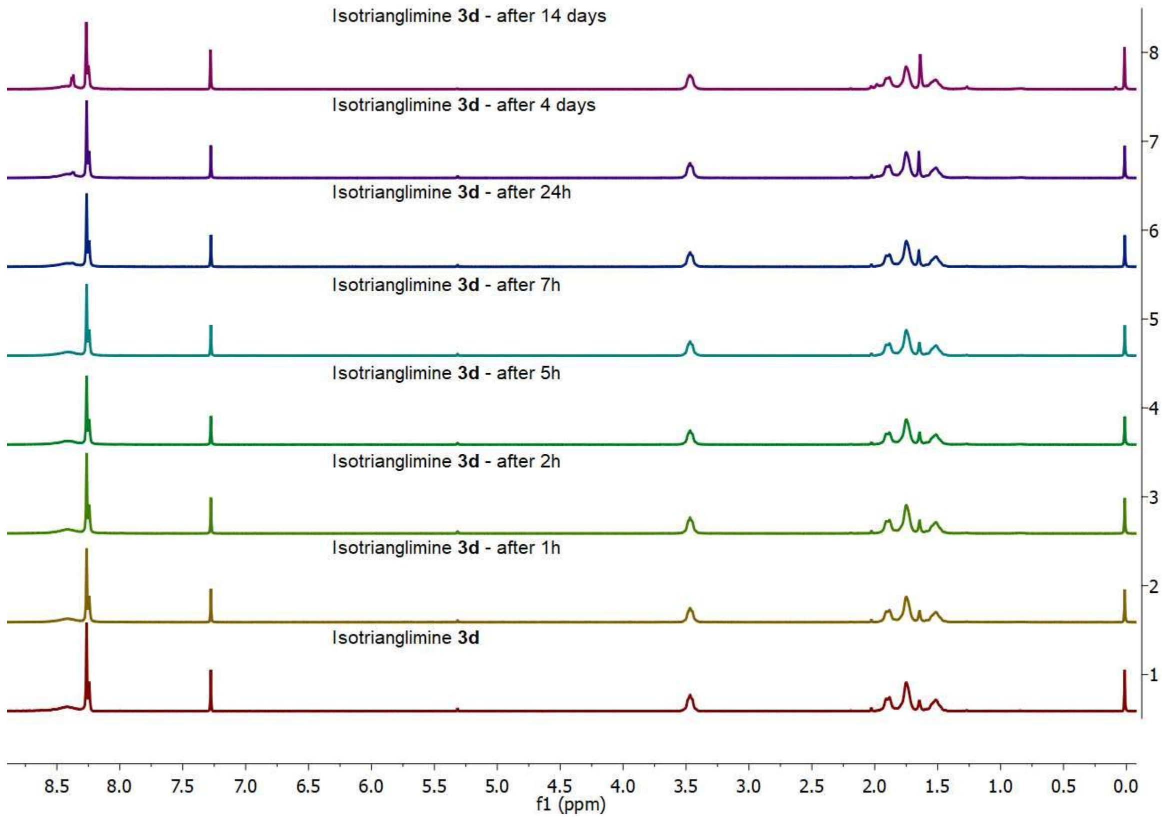

Figure S5. The ^1H NMR spectra recorded at certain time intervals for the solution of isotrianglimine **3d** (all spectra were measured in $CDCl_3$, 300 MHz, 20 °C).

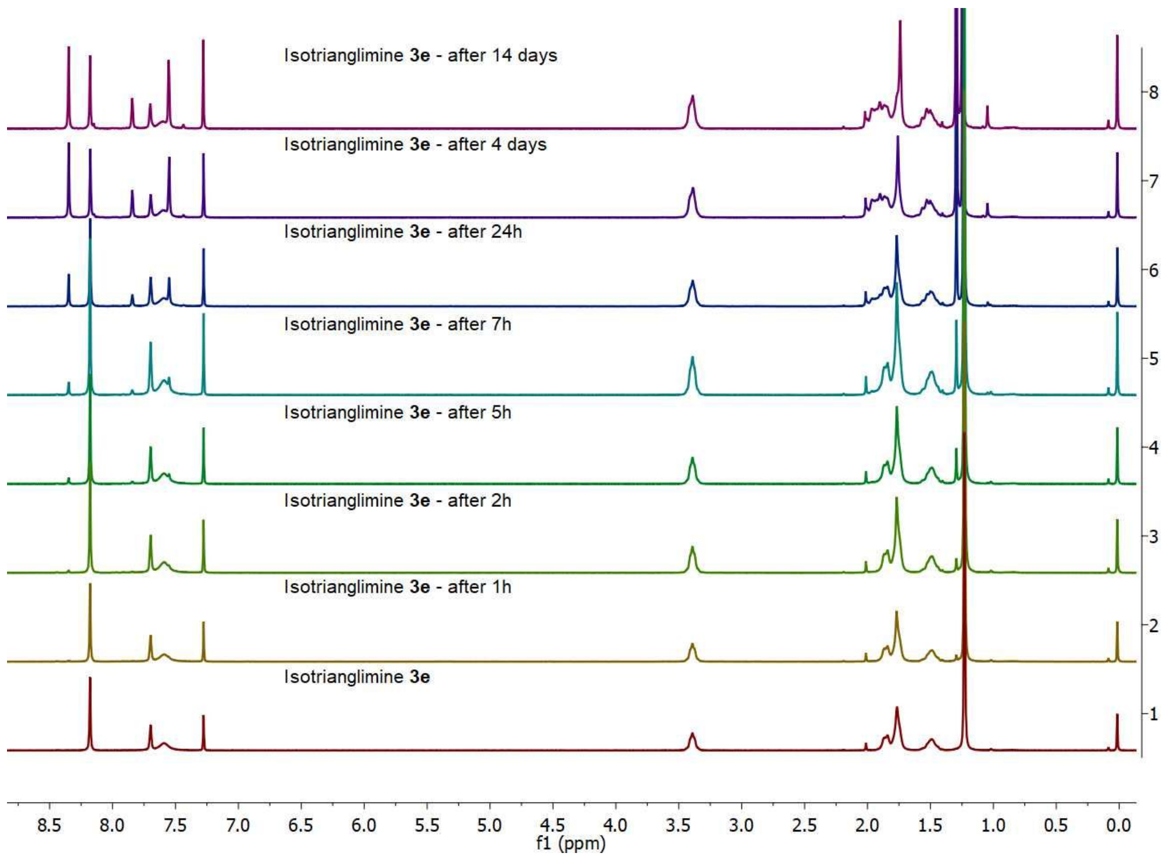

Figure S6. The ^{1}H NMR spectra recorded at certain time intervals for the solution of isotrianglimine **3e** (all spectra were measured in $CDCl_3$, 300 MHz, 20 °C).

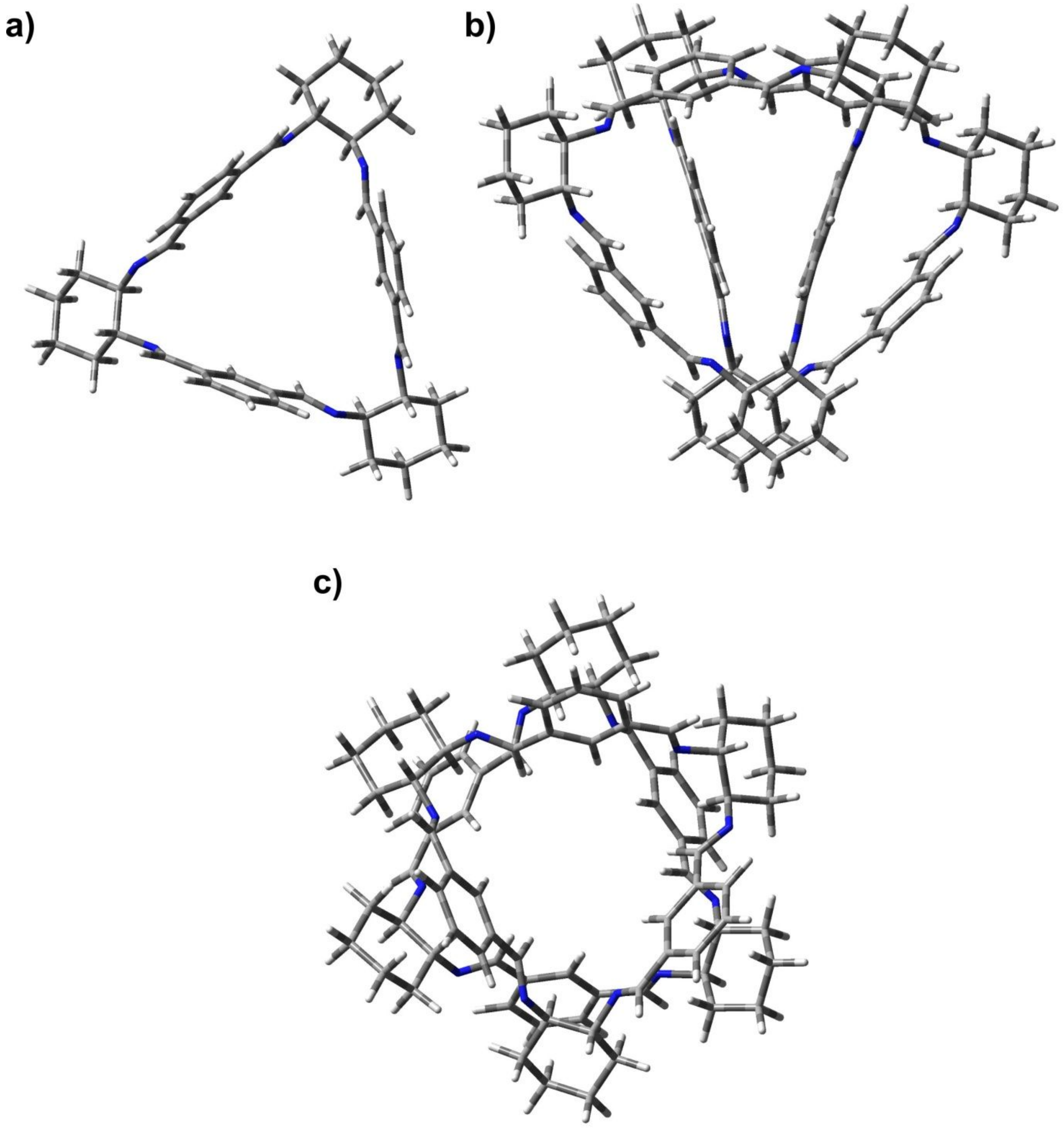

Figure S7. Structures of low-energy a) monomer, b) tail-to-tail and c) head-to-head (capsule) dimers of isotrianglimine **3a**, calculated at the IEFPCM/B3LYP-GD3BJ/6-311G(d,p) level of theory.

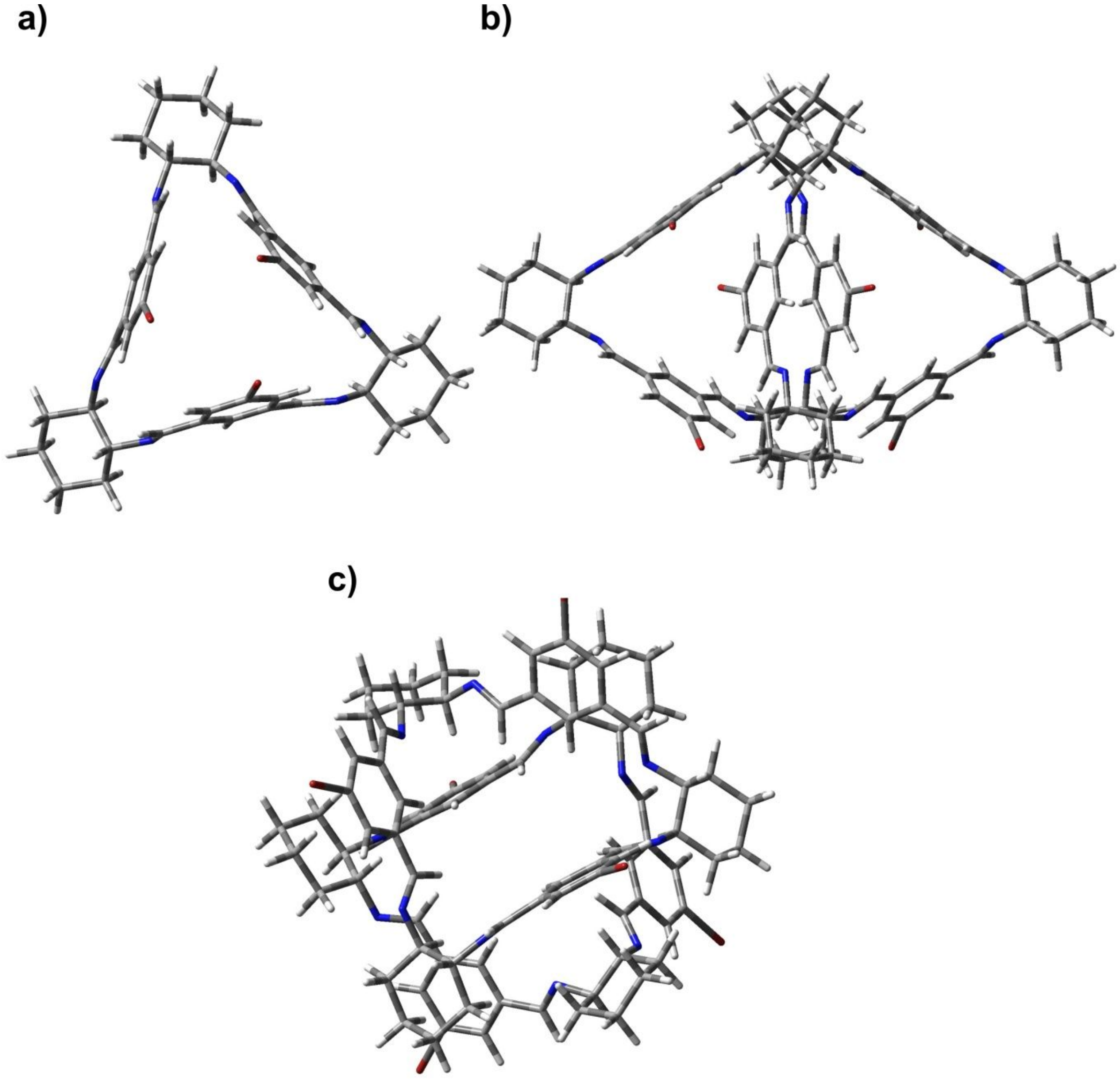

Figure S8. Structures of low-energy a) monomer, b) tail-to-tail and c) head-to-head (capsule) dimers of isotrianglimine **3b**, calculated at the IEFPCM/B3LYP-GD3BJ/6-311G(d,p) level of theory.

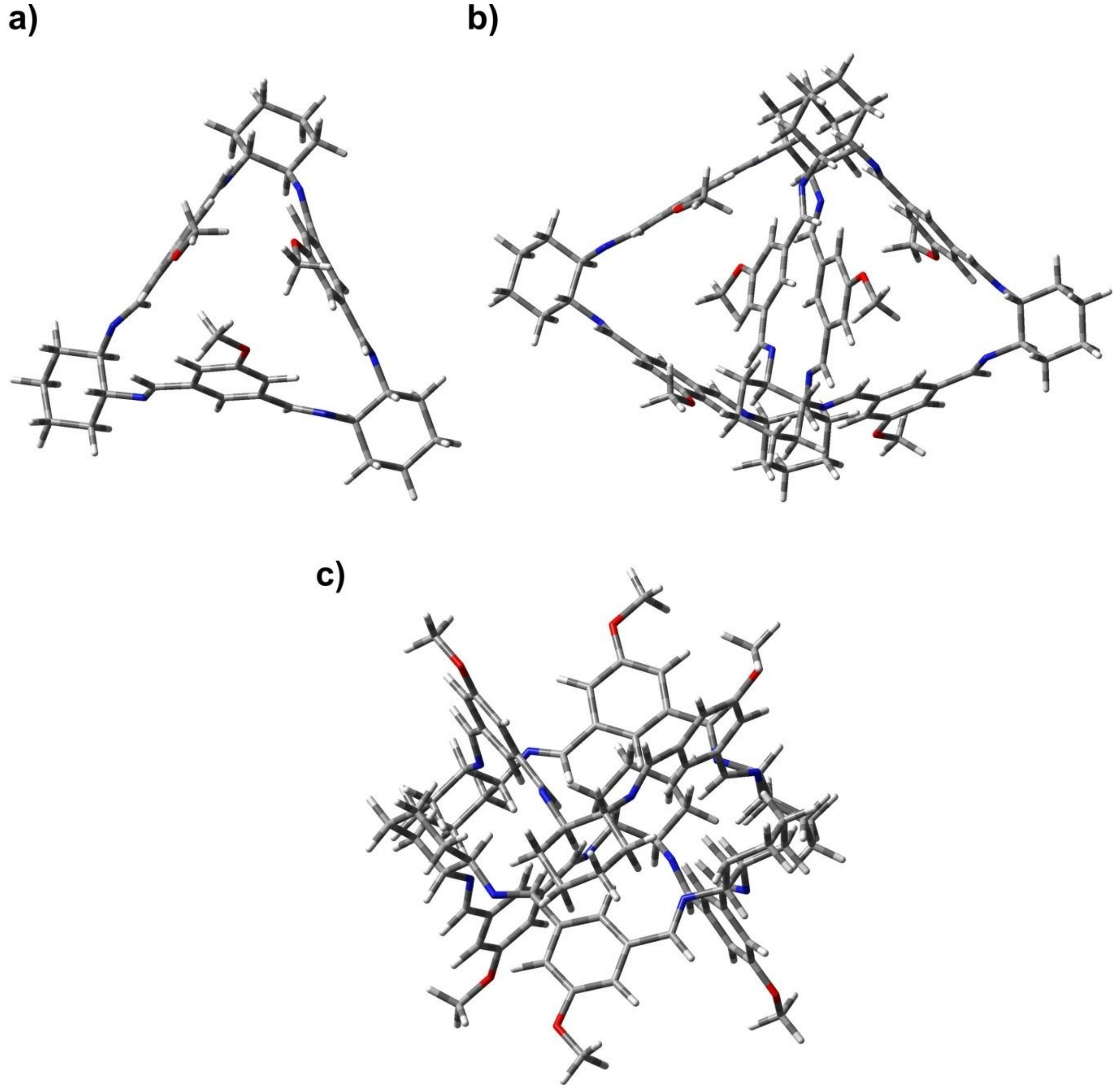

Figure S9. Structures of the lowest energy a) monomer, b) tail-to-tail and c) head-to-head (capsule) dimers of isotrianglimine **3c**, calculated at the IEFPCM/B3LYP-GD3BJ/6-311G(d,p) level of theory.

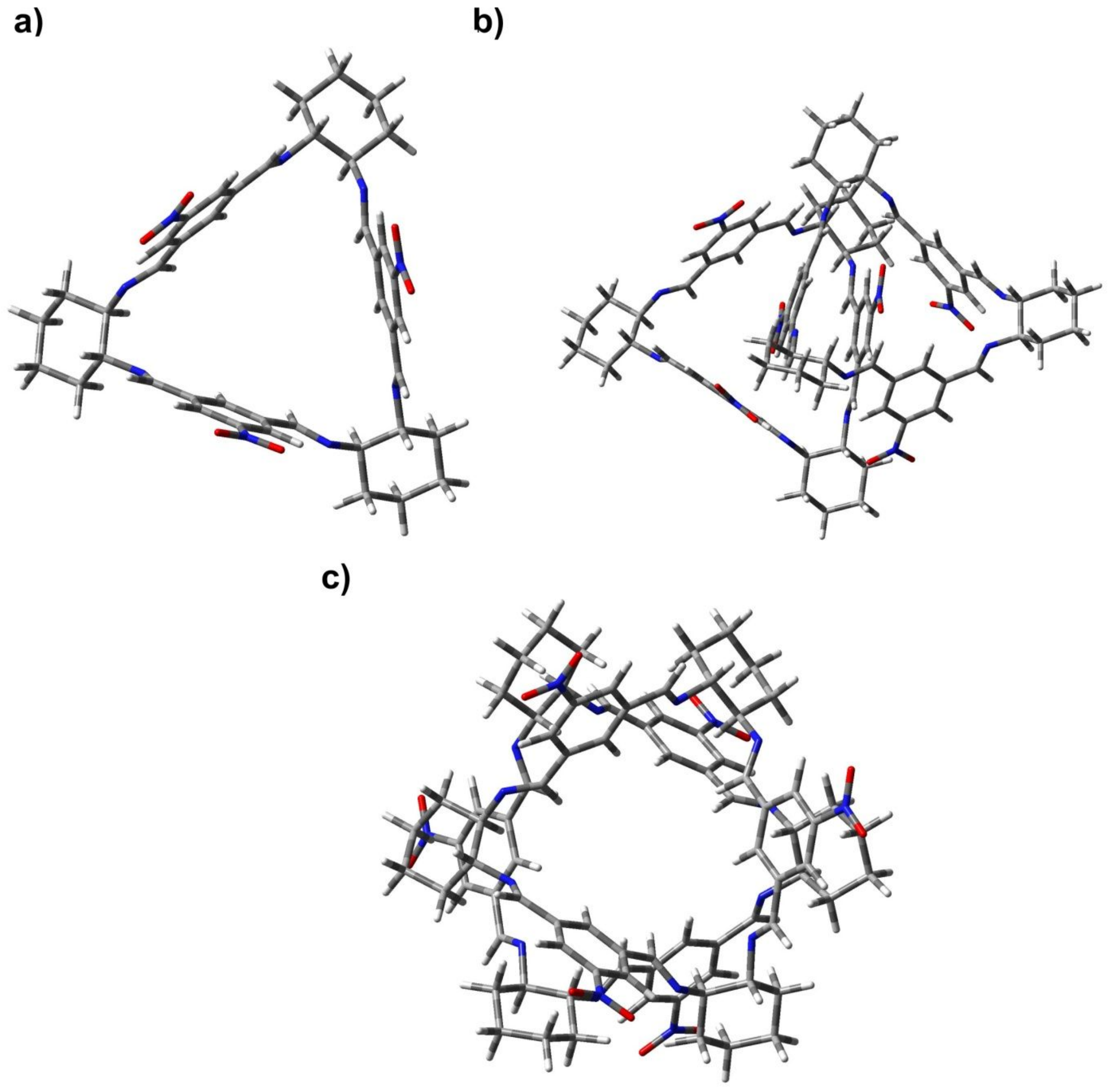

Figure S10. Structures of low-energy a) monomer, b) tail-to-tail and c) head-to-head (capsule) dimers of isotrianglimine **3d**, calculated at the IEFPCM/B3LYP-GD3BJ/6-311G(d,p) level of theory.

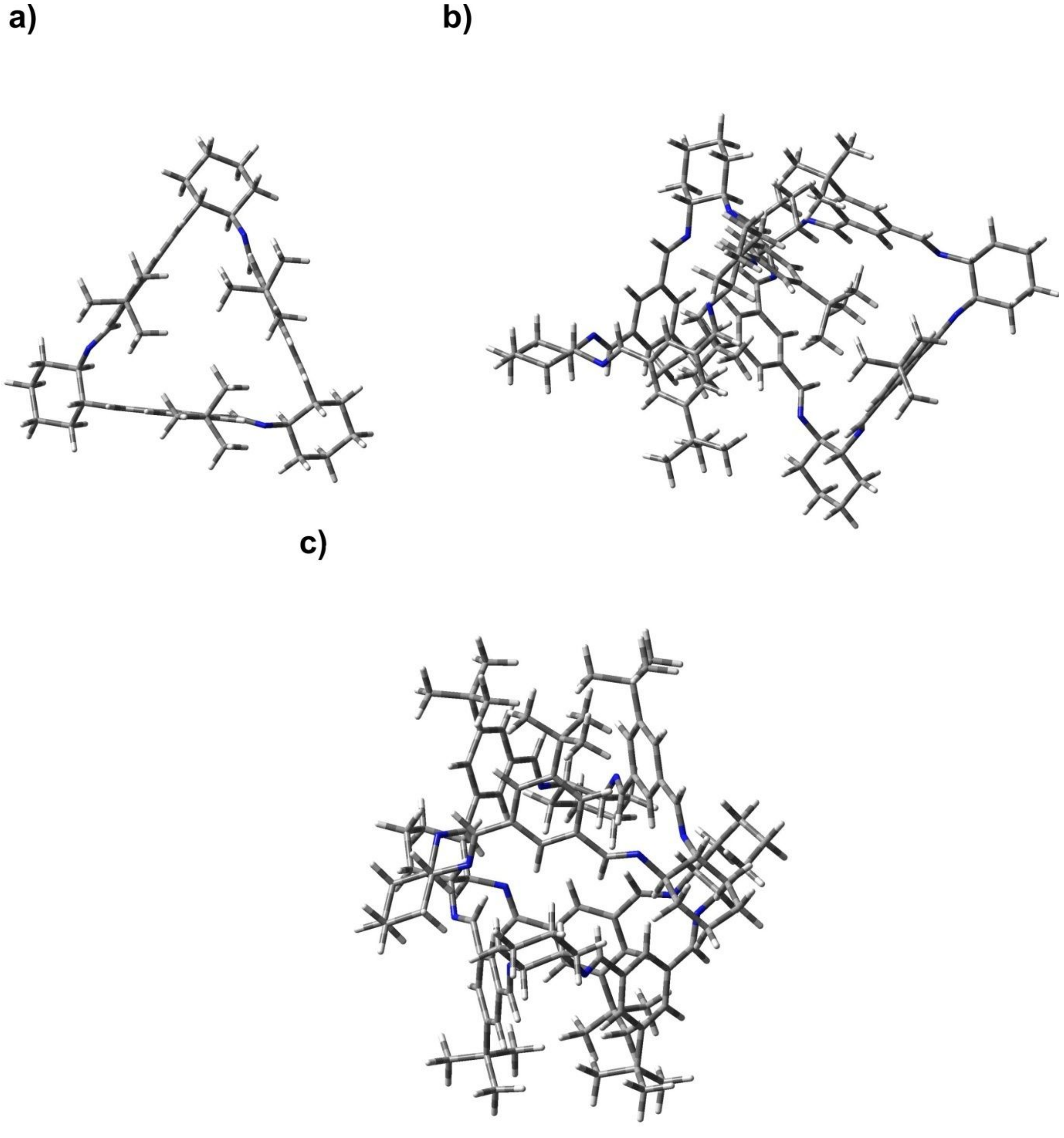

Figure S11. Structures of low-energy a) monomer, b) tail-to-tail and c) head-to-head (capsule) dimers of isotrianglimine **3e**, calculated at the IEFPCM/B3LYP-GD3BJ/6-311G(d,p) level of theory.

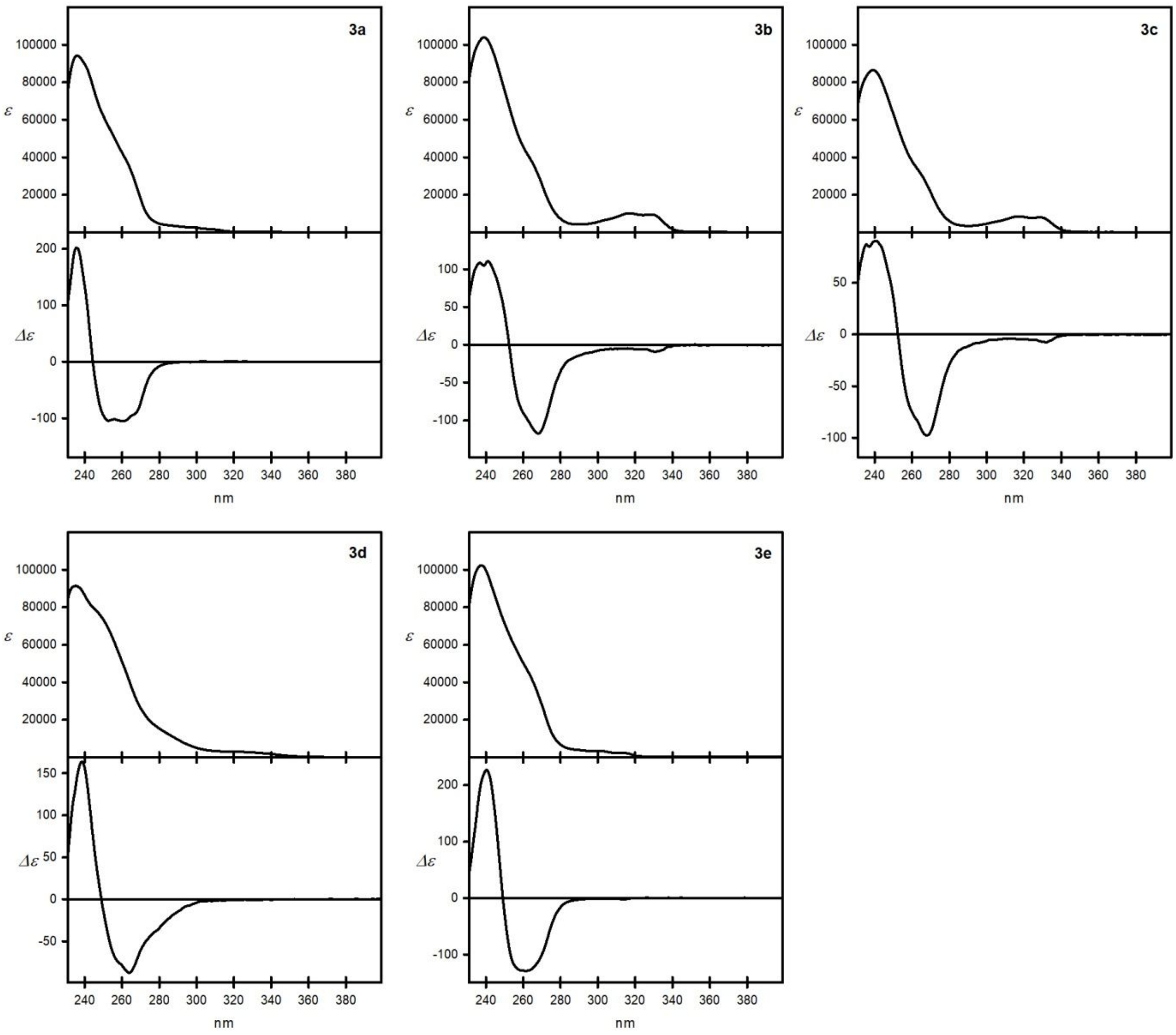

Figure S12. UV (upper panels) and ECD (lower panels) spectra of isotrianglimines **3a**-**3e** measured in chloroform solutions.

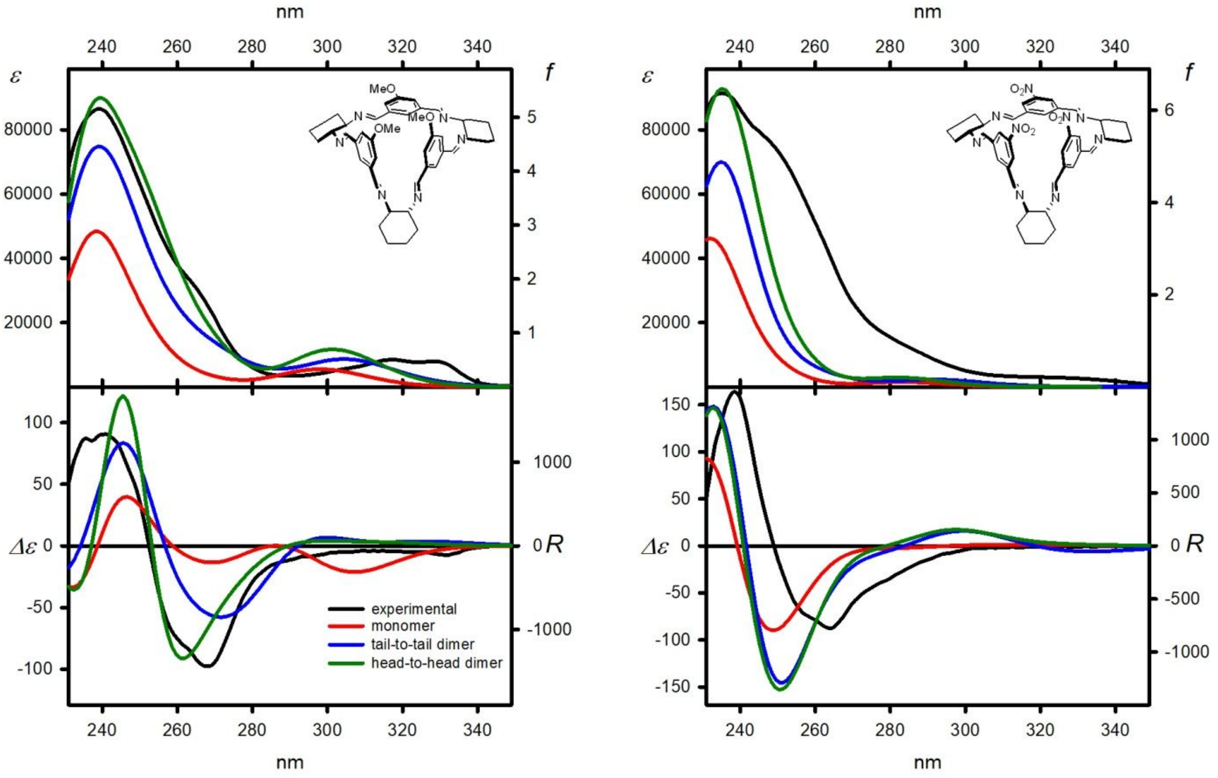

Figure S13. UV (upper panels) and ECD (lower panels) spectra of isotrianglimines a) **3c** and b) **3d** measured in chloroform solutions (black lines) and calculated for respective monomers (red lines), tail-to-tail dimers (blue lines) and capsules (green lines) of isotrianglimines **3c** and **3d** at the IEFPCM/M06-2X/6-311G(d,p) level. The wavelengths have been corrected to match experimental UV maxima.

5. Single-Crystal X-ray diffraction

Reflection intensities for **3a_1**, **3a_2**, **3a_3**, **3b_2, 3d** and **3e** were measured on a SuperNova diffractometer equipped with a Cu microfocus source (λ = 1.54178 Å) and a 135 mm Atlas CCD detector while for **3b_1** and **3c** were measured on a Xcalibur diffractometer (Eos detector) equipped with a graphite monochromator and MoKα radiation (λ = 0.71073 Å). In all single-crystal experiments, except for **3b_1**, **3c** and **3d**, the temperature of the crystals was controlled at 130K with an Oxford Instruments Cryosystem cold nitrogen-gas blower. The remaining X-ray experiments were carried out at a temperature of 294 K. Data reduction and analysis were carried out with the CrysAlisPro software. All crystal structures were solved by direct methods using the SHELXT[16] program and were refined by full matrix least squares on F^2 using the program SHELXL-2018[16] through the graphical interface X-Seed.[17] Non-hydrogen atoms were refined anisotropically, except for atoms constituting the acetonitrile molecule in **3b_2** for which the occupancy factors were refined to 0.5. The hydrogen atoms bound to C atoms were placed at calculated positions and refined using a riding model, and their isotropic displacement parameters were given a value 20% higher than the isotropic equivalent for the atom to which the H atoms were attached (for methyl hydrogen atoms this value has been increased to 50%).

Crystals of **3b_1** and **3c** were weakly diffracting; the diffraction limit was only 0.95 Å (for **3b_1**) and 1.21 Å (for **3c**), and led to collection of a poor quality data. This poor quality data is reflected in the checkcif reports for both crystal structures as Alert B or C (*e.g.* low bond precision on C-C bonds, missing reflections or too low ratio observed/unique reflections).

When it was not possible to determined of the position of solvent molecules that occupy both internal and external cavities, their contribution was subtracted from the diffraction data using the SQUEEZE as implemented in PLATON.[18]

The absolute structure of the investigated crystals was assumed from the known absolute configuration of the *trans*-(*R*,*R*)-1,2-diaminecyclohexane which was used as a starting material in the syntheses. Graphical images were produced in Xseed [17] using Pov-Ray [19] and Mercury [20] programs. The relevant crystal data collection and refinement parameters are listed in Table S3.

CCDC 2096600-2096607 contain the supplementary crystallographic data for this paper. These data can be obtained free of charge from The Cambridge Crystallographic Data Centre via www.ccdc.cam.ac.uk/data%5Frequest/cif

Table S3. Crystal data and structure refinement for **3a**-**3e**.

	**3a_1**	**3a_2**	**3a_3**	**3b_1**	**3b_2**	**3c**	**3d**	**3e**
Chemical formula	$C_{42}H_{48}N_6$•0.5(C_7H_8)	$C_{28}H_{32}N_4$	2($C_{42}H_{48}N_6$)·C_8H_{10}	$C_{42}H_{45}Br_3N_6$	4($C_{42}H_{45}Br_3N_6$)• C_2H_3N•10(O)	$C_{45}H_{54}N_6O_3$• 0.2(H_2O)	$C_{42}H_{45}N_9O_6$	$C_{54}H_{72}N_6$·3(C_2H_3N)
M_r	682.93	636.86	1379.88	873.57	3695.32	730.54	771.87	928.33
Crystal system, space group	Hexagonal, $P6_3$	Hexagonal, $P6_3$	Monoclinic, $P2_1$	Monoclinic, $P2_1$	Monoclinic, $P2_1$	Monoclinic, $P2_1$	Monoclinic, $P2_1$	Monoclinic, $P2_1$
Temperature (K)	130	130	130	294	130	294	294	130
a, *b*, *c* (Å)	20.5141 (2), 6.0135 (1)	20.4947 (14), 5.9933 (3)	14.7530 (1), 15.8771 (1), 17.2266 (1)	14.656 (1), 19.048 (1), 15.135 (1)	12.621 (1), 22.974 (1), 16.111 (1)	14.0360 (7), 20.1550 (9), 15.2140 (7)	14.6077 (1), 18.9581 (1), 15.2796 (1)	10.7754 (1), 19.8015 (2), 14.4698 (1)
$\mathcal{T}$ (°)	90	90	93.850 (1)	92.228 (3)	103.456 (3)	93.188 (5)	92.990 (1)	107.685 (1)
V (Å^3)	2191.61 (6)	2180.1 (3)	4025.96 (4)	4222.0 (5)	4543.2 (5)	4297.3 (3)	4225.68 (5)	2941.50 (5)
Z	2	2	2	4	1	4	4	2
Radiation type	Cu *K*a	Cu *K*a	Cu *K*a	Mo *K*a	Cu *K*a	Mo *K*a	Cu *K*a	Cu *K*a
m (mm^{-1})	0.47	0.44	0.52	2.90	3.62	0.07	0.68	0.48
Crystal size (mm)	0.45 × 0.06 × 0.06	0.41 × 0.08 × 0.08	0.26 × 0.12 × 0.05	0.45 × 0.38 × 0.16	0.53 × 0.36 × 0.30	0.55 × 0.13 × 0.10	0.60 × 0.40 × 0.40	0.50 × 0.13 × 0.08
Absorption correction	Multi-scan	Multi-scan	Multi-scan	Multi-scan	Multi-scan	Multi-scan	Multi-scan	Multi-scan
T_{min}, T_{max}	0.589, 1.000	0.065	0.893, 1.000	0.615, 1.000	0.776, 1.000	0.978, 1.000	0.734, 1.000	0.592, 1.000
No. of measured, independent and observed [$I > 2\sigma(I)$] reflections	18657, 2998, 2924	15371, 3011, 2145	59687, 16475, 15875	36159, 14594, 9551	43057, 18789, 18130	32990, 15127, 5606	38825, 17294, 16594	25298, 12047, 11665
$R[F^2 > 2\sigma(F^2)]$, $wR(F^2)$, *S*	0.041, 0.124, 1.07	15371, 3011, 2145	0.032, 0.081, 1.03	0.045, 0.096, 1.01	0.039, 0.115, 1.08	0.077, 0.147, 0.95	0.038, 0.115, 1.02	0.046, 0.126, 1.06
$\Delta\rho_{max}$, $\Delta\rho_{min}$ (e Å^{-3})	0.44, -0.15	0.28, -0.29	0.23, -0.19	0.74, -0.55	0.55, -0.75	0.15, -0.18	0.24, -0.26	0.33, -0.24
Absolute structure parameter	-0.1 (2)	-0.8 (6)	-0.05 (8)	-0.005 (4)	-0.022 (8)	-2.7 (10)	0.03 (5)	0.46 (19)

Table S4. The estimated size of the macrocyclic cavity in the investigated crystals.

		Size of the the inner cavity* [Å]			Mean size of the inner cavity [Å]
**3a_1**		7.23	7.23	7.23	7.23
**3a_2**		7.22	7.22	7.22	7.22
**3a_3**	mol1	7.19	7.38	7.66	7.41
	mol2	7.02	7.42	7.53	7.32
**3b_1**	mol1	6.58	7.00	7.11	6.90
	mol2	6.92	6.95	7.10	6.99
**3b_2**	mol1	6.99	7.02	7.56	7.19
	mol2	6.80	7.00	7.87	7.22
**3c**	mol1	6.29	6.63	7.17	6.70
	mol2	6.20	6.74	7.01	6.65
**3d**	mol1	6.93	6.97	7.38	7.09
	mol2	6.59	7.08	7.16	6.94
**3e**		5.99	6.10	6.13	6.07

Crystals suitable for single-crystal X-ray diffraction were obtained by the slow evaporation of toluene (**3a_1**) and *p*-xylene (**3a_2**) solution under ambient conditions. In the crystal structures of **3a_1** and **3a_2**, the unit cell contains symmetry independent isotrianglimine molecule that occupies 3 site symmetry. The host matrix of viewed along [001] shows two types of channels: narrow and wide, both able to accommodate solvent molecules. The narrow channels pass through the molecular cavity (interior channels along 3-fold axis) while the wide channels are formed in the intermolecular space (exterior channels along 6_3 screw axis). The guest molecules occupying the interior channels are placed at special positions on 3 site symmetry and hence are disordered over three equal positions required by the symmetry. While in **3a_1** we could model properly the disordered toluene molecule, in **3a_2** it turned out to be impossible. In both crystal structures guest molecules located in the interior channels running along 6_3 screw axis were highly disordered and could not be resolved to yield a satisfactory model, therefore their unresolved electron density was treated with SQUEEZE. In **3a_1** the estimated electron count is 38 in an accessible void volume of 179.3 Å^3 and can indicate to squeezed of 0.76 molecule of toluene per unit cell (38/50=0.76). The estimated electron counts 183 in an accessible void volume of 503.7 Å^3 in **3a_2** which correspond to squeezed of 3 molecules of *p*-xylene per unit cell.

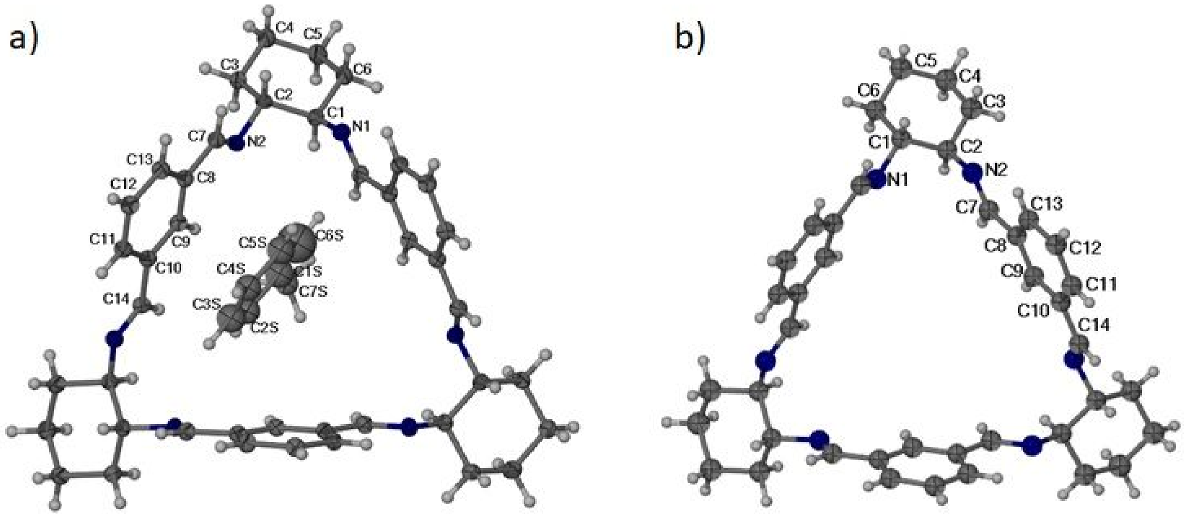

Figure S14. A perspective view of a) **3a_1** and b) **3a_2**. Labels mark the symmetry independent part of molecules. Ellipsoids are drawn at the 40% probability level, hydrogen atoms are represented by spheres of arbitrary radii.

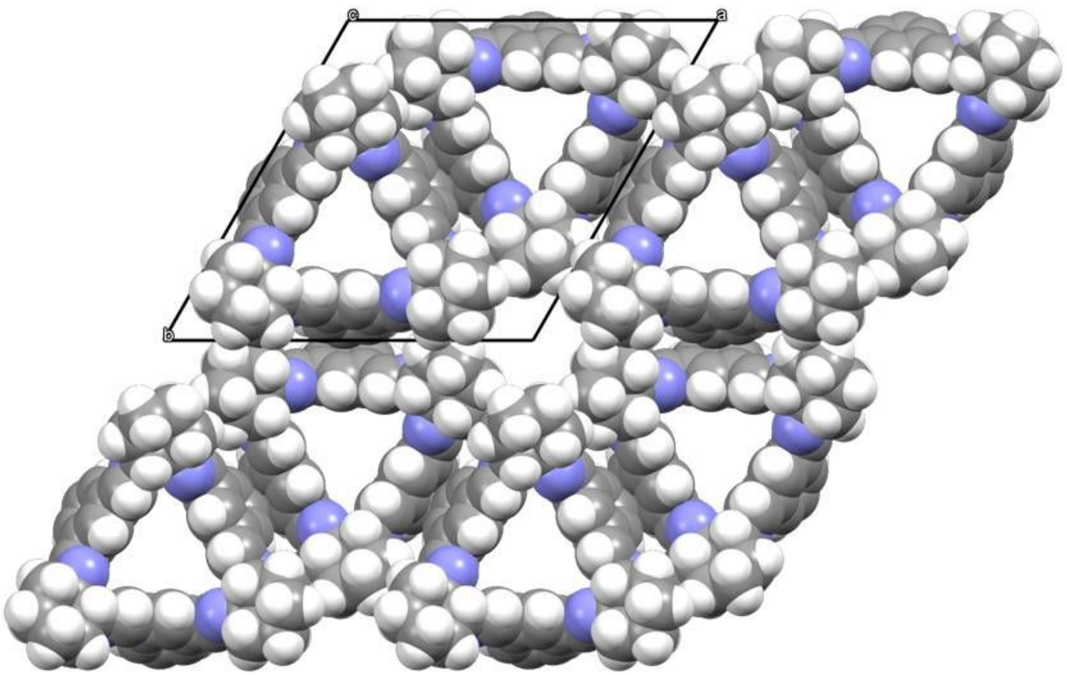

Figure S15. A packing diagram of macrocycles in the crystals of **3a_1** and **3a_2** as viewed along the *c* lattice direction. The host molecules are shown in space-filling style.

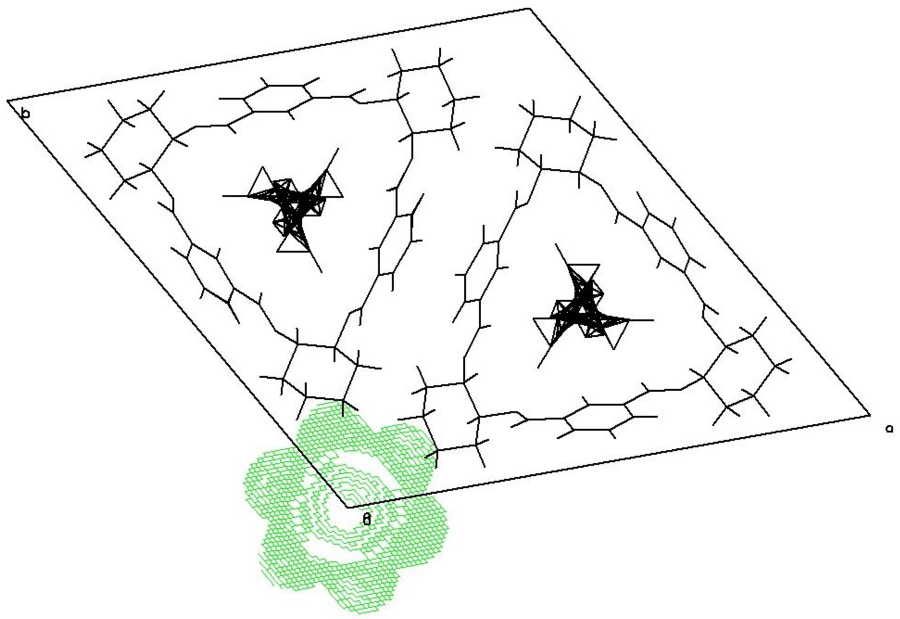

Figure S16. A packing diagram of **3a_1** showing the void (in green) from which the solvent molecules were removed. The view is along the *c* lattice direction.

Crystals of **3a_3** suitable for single-crystal X-ray diffraction were obtained by the slow evaporation of *m*-xylene solution under ambient conditions. The asymmetric unit cell is comprised of symmetry independent macrocyclic molecules that form interdigitated dimeric motif and *m*-xylene molecule included in the internal space. The volume of each void as calculated by the 1.5 Å probe radius is 84 Å^3.

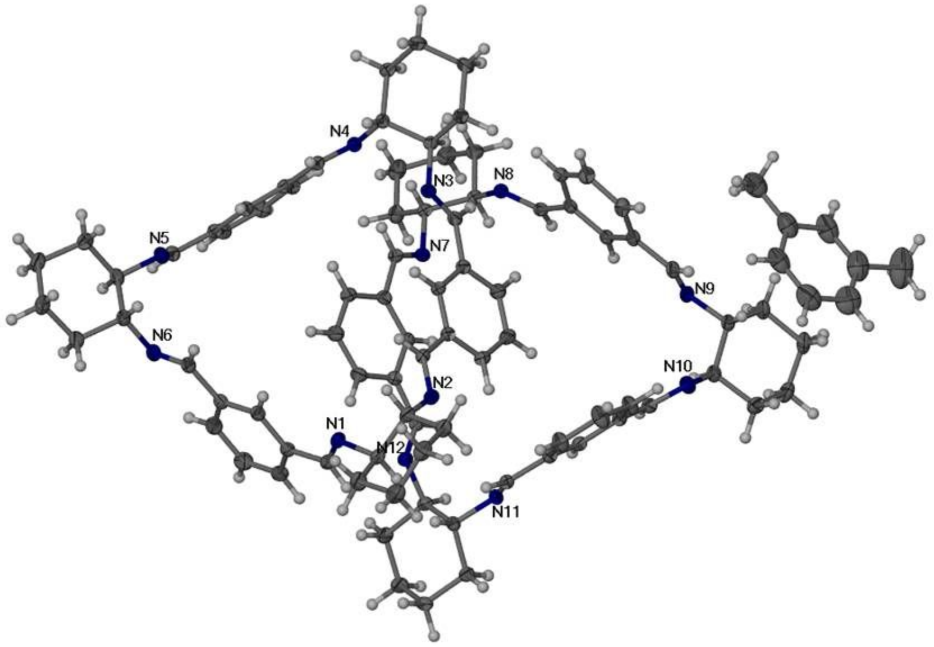

Figure S17. An illustration of asymmetric unit of **3a_3**. For clarity only the heteroatoms were labeled. Ellipsoids are drawn at the 40% probability level, hydrogen atoms are represented by spheres of arbitrary radii.

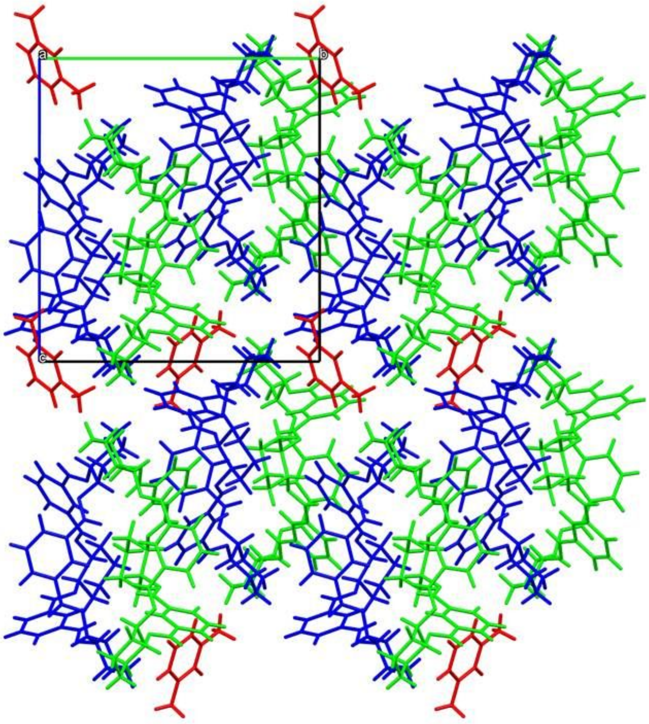

Figure S18. A packing diagram of **3a_3** as viewed along the *a* lattice direction. The two symmetry independent host molecules are shown in green and blue whereas *m*-xylene molecules are shown in red.

a)

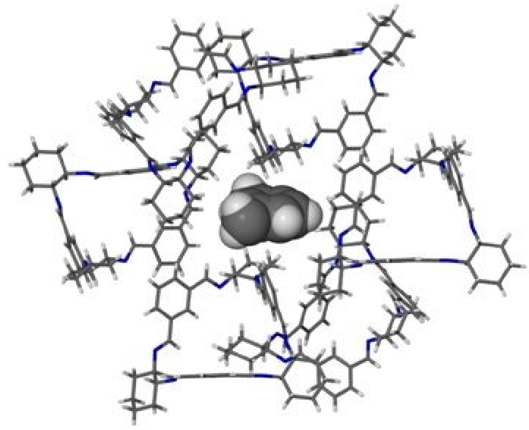

b)

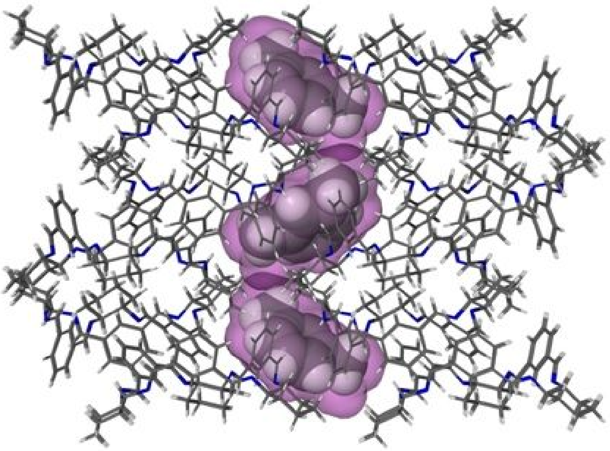

Figure S19. a) Top and b) side view of the internal space in **3a_3**. The host molecules are shown in stick style whereas the guest molecules are shown as space-filling models. The solvent accessible volume of 84 Å^3 is shown as pink Connolly surfaces[21] using a probe radius of 1.5 Å.

Crystals of **3b_1** suitable for single-crystal X-ray diffraction were obtained by the slow evaporation of acetonitrile and dichloromethane mixture under ambient conditions. The asymmetric unit cell contains two molecules of **3b**.

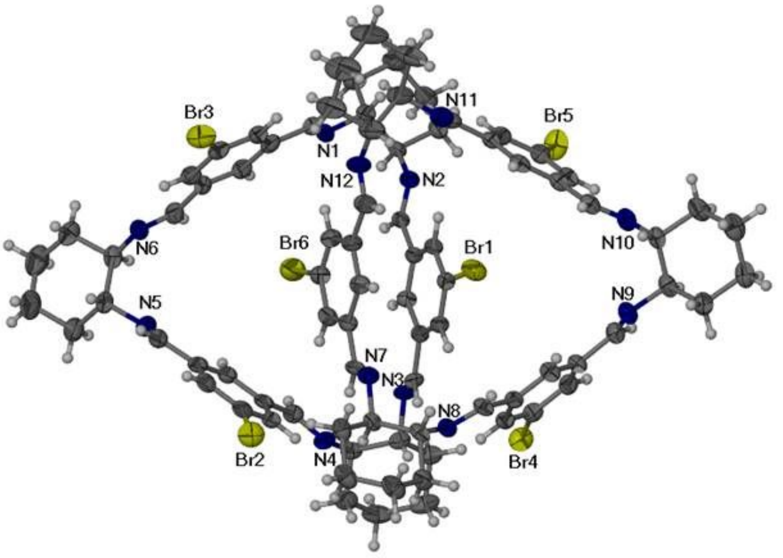

Figure S20. An illustration of asymmetric unit of **3b_1**. For clarity only the heteroatoms were labeled. Ellipsoids are drawn at the 40% probability level, hydrogen atoms are represented by spheres of arbitrary radii. Two symmetry independent macrocyclic molecules form interdigitated dimeric motif.

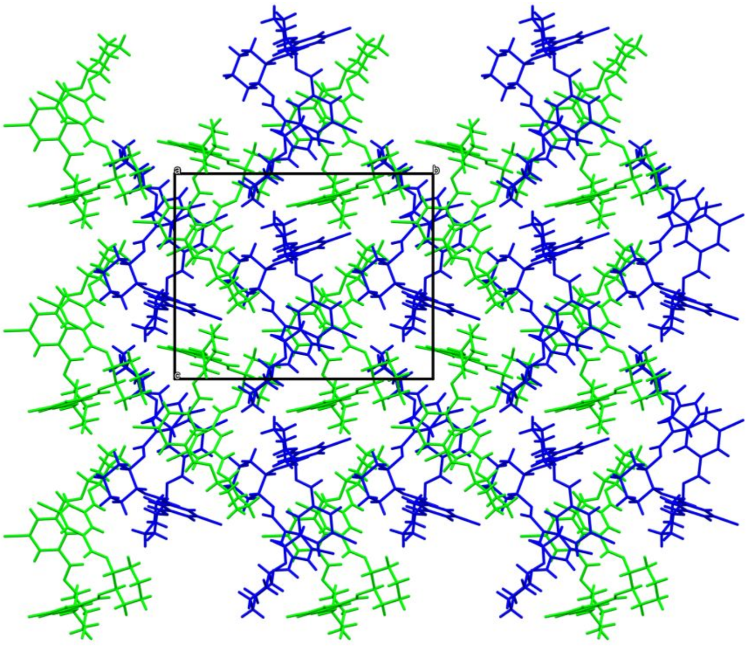

Figure S21. A packing diagram of **3b_1** as viewed along the *a* lattice direction. The two symmetry independent host molecules are shown in green and blue in stick style.

Crystals of **3b_2** suitable for single-crystal X-ray diffraction were obtained by the slow evaporation of acetonitrile under ambient conditions. The asymmetric unit cell contains two molecules of **3b**, five water molecules and a half molecule of acetonitrile. The ratio of host to guests is 4:10:1.

Since some of water molecules are highly disordered it could not be modelled properly; therefore, its contribution was removed from the diffraction data using SQUEEZE. The estimated electron count is 5 in an accessible void volume of 355.6 Å^3 and is correlated with approximately 0.5 water molecules per unit cell.

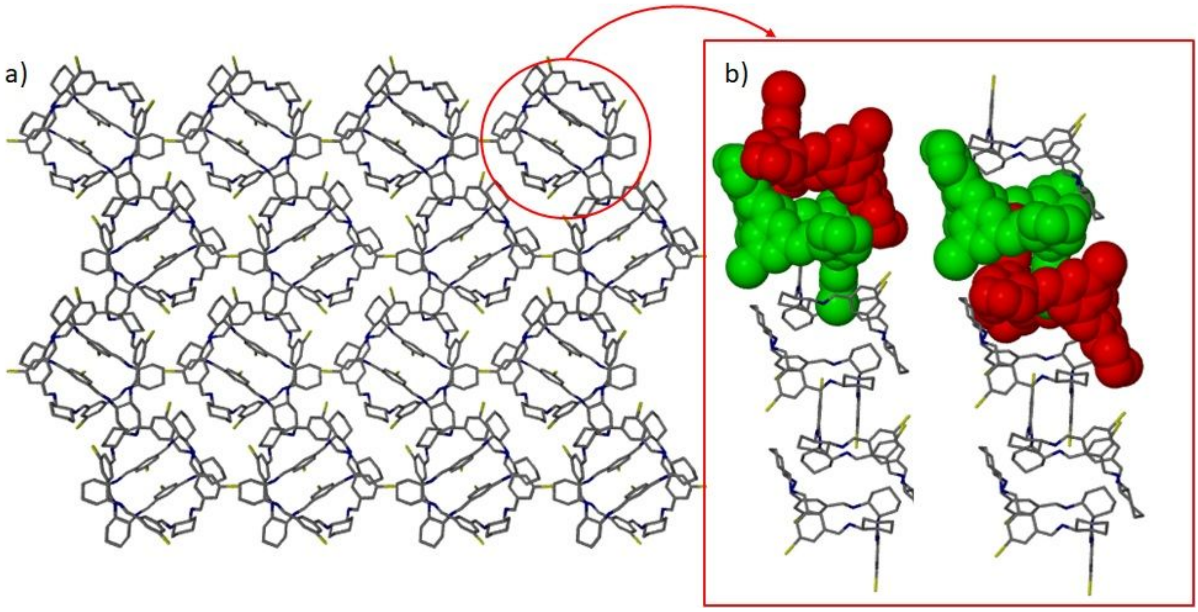

Figure S22. a) A packing diagram of **3b_2** as viewed along the *a* lattice direction. b) The column consists of two symmetry independent host molecules that form capsules and interdigitated dimeric motifs. These motifs are shown as space-filling model. All H atoms were omitted for clarity.

Crystals of **3c** suitable for single-crystal X-ray diffraction were obtained by the slow evaporation a mixture of acetonitrile and dichloromethane under ambient conditions. The asymmetric unit cell is comprised of symmetry independent macrocyclic molecules that form interdigitated dimeric motif and a water molecule that occupies extrinsic void. The occupancy factor for water molecules was estimated as 0.4. The ratio of host to guests is 1:5.

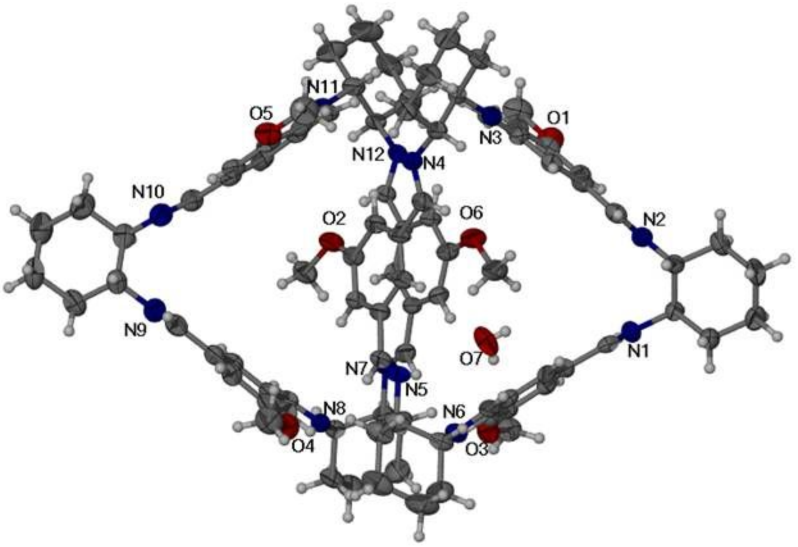

Figure S23. An illustration of asymmetric unit of **3c**. For clarity only the heteroatoms were labeled. Ellipsoids are drawn at the 40% probability level, hydrogen atoms are represented by spheres of arbitrary radii.

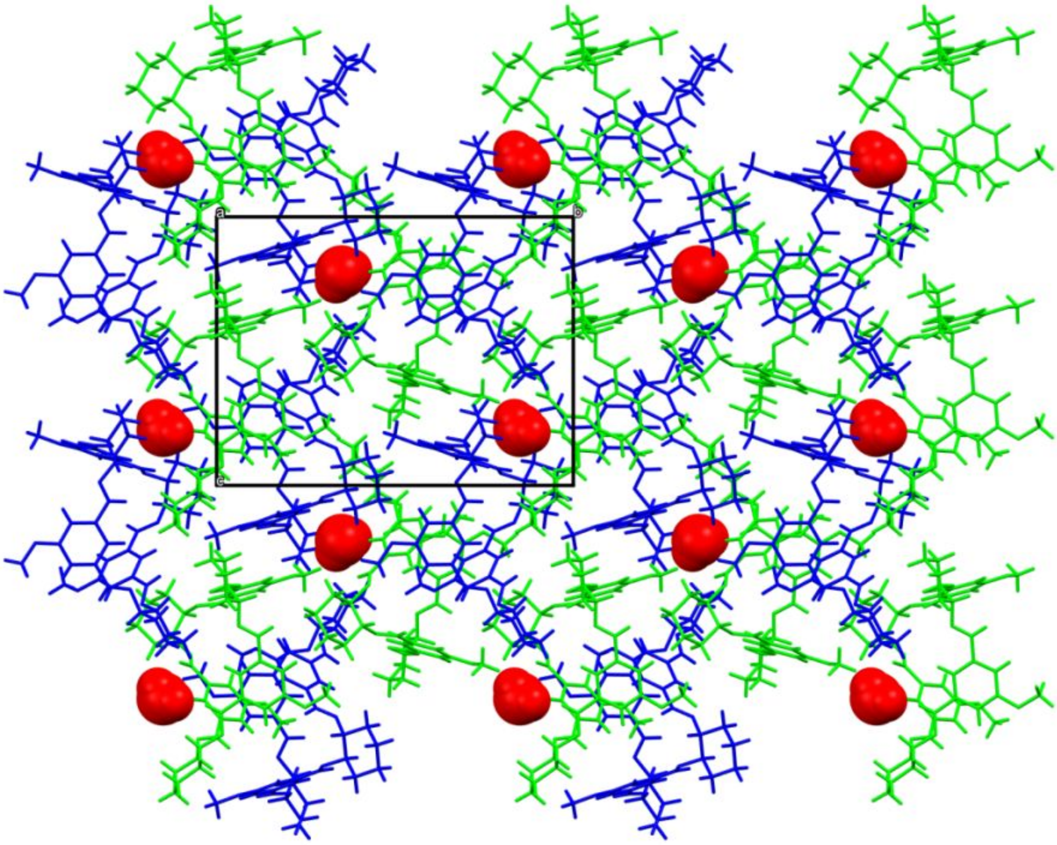

Figure S24. A packing diagram of **3c** as viewed along the *a* lattice direction. The two symmetry independent host molecules are shown in green and blue in stick style whereas water molecules are shown as space-filling model.

Crystals of **3d** suitable for single-crystal X-ray diffraction were obtained by the slow evaporation of acetonitrile and dichloromethane mixture under ambient conditions. The asymmetric unit cell contains two molecules of **3d.**

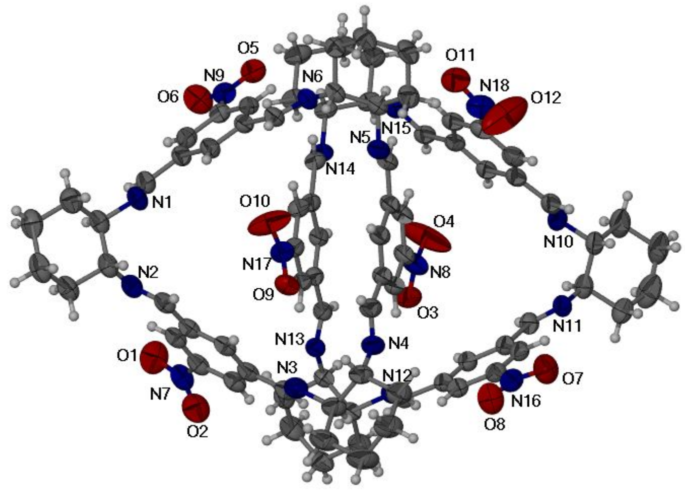

Figure S25. Asymmetric unit of **3d**. For clarity only the heteroatoms were labeled. Ellipsoids are drawn at the 40% probability level, hydrogen atoms are represented by spheres of arbitrary radii. Two symmetry independent macrocyclic molecules form interdigitated dimeric motif.

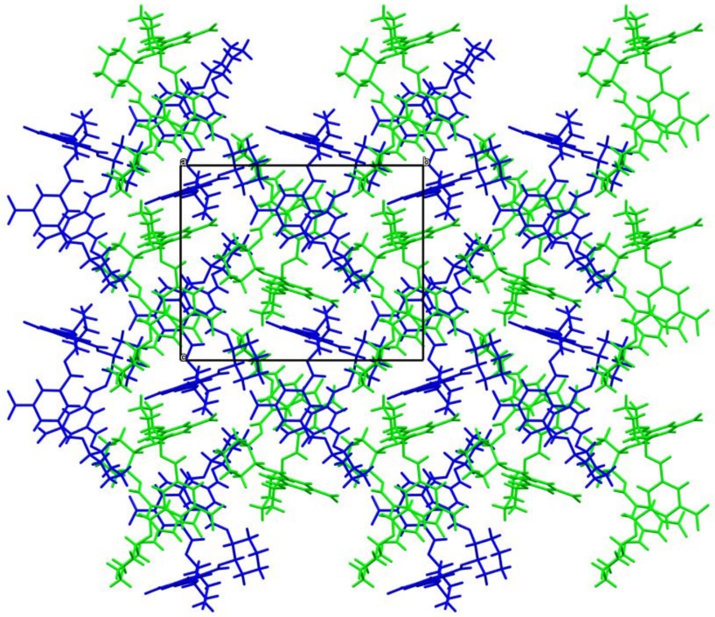

Figure S26. A packing diagram of **3d** as viewed along the *a* lattice direction. The two symmetry independent host molecules are shown in green and blue in stick style.

Crystals of **3e** suitable for single-crystal X-ray diffraction were obtained by the slow evaporation of acetonitrile under ambient conditions. The asymmetric unit cell contains one molecule of **3e** and three molecules of acetonitrile. The ratio of host to guests is 1:3.

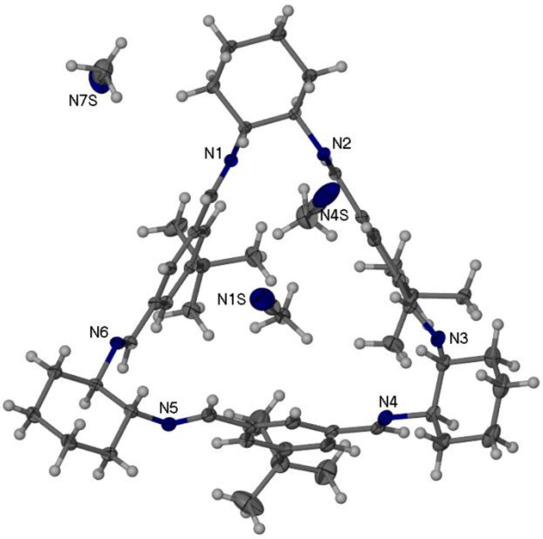

Figure S27. Asymmetric unit cell of **3e** contains a molecule of isotrianglimine and three molecules of acetonitrile. For clarity only the heteroatoms were labeled. Ellipsoids are drawn at the 40% probability level, hydrogen atoms are represented by spheres of arbitrary radii.

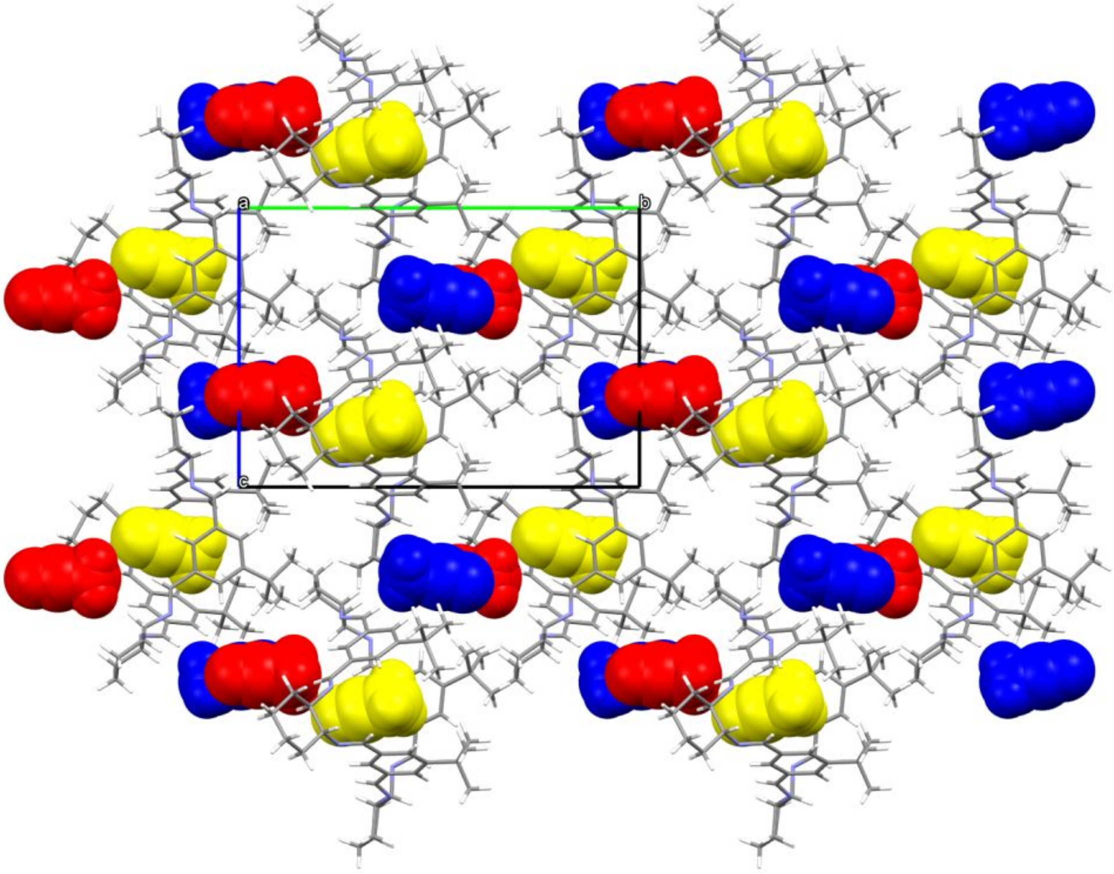

Figure S28. A packing diagram of **3e** as viewed along the *a* lattice direction. The host molecules are shown in capped sticks style whereas guest molecules are shown as space-filling model. The colors represent symmetry independent acetonitrile molecules.

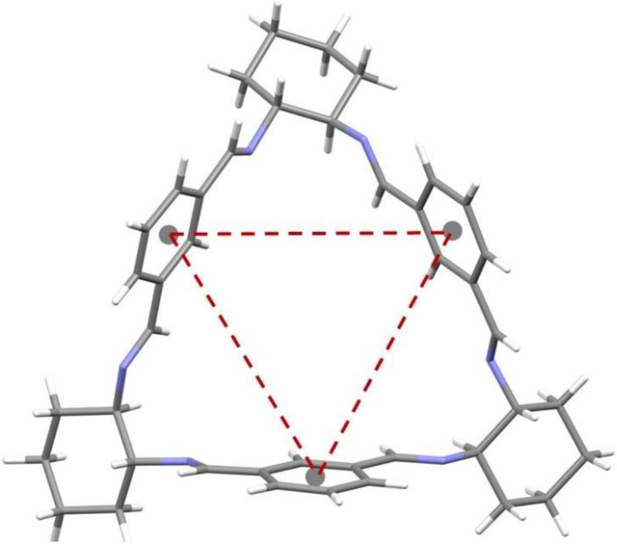

Figure S29. Definition of the method of calculating the size of the macrocyclic cavity.

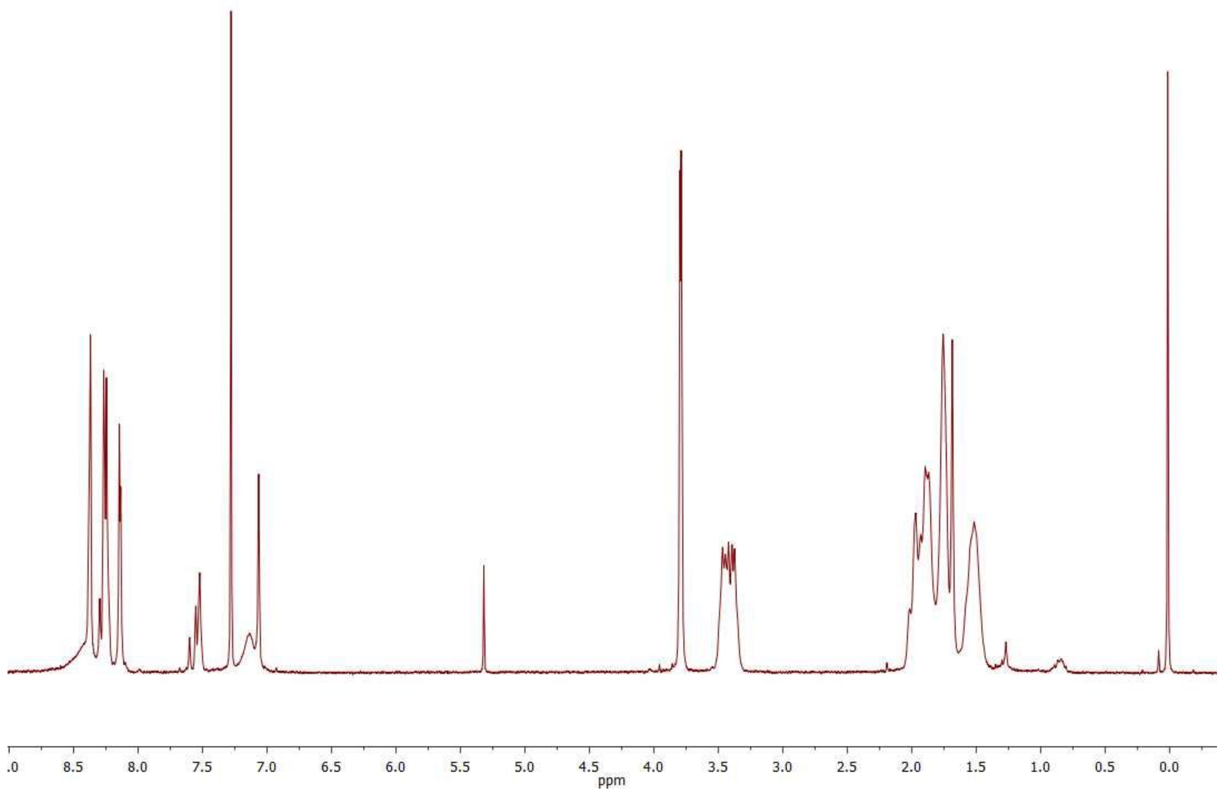

Figure S30. The ^{1}H NMR spectrum recorded for the crude reaction mixture obtained in reaction between equimolar amounts of dialdehydes **2c** (0.5 eq) and **2d** (0.5 eq) and doubled the amount of (*R*,*R*)-**1** ($CDCl_3$, 300 MHz, 20 °C).

6. Cartesian coordinates (IEFPCM/B3LYP-GD3BJ/6-311G(d,p) method)

Compound				
**3a** (m)	C	2.00580000	7.52640000	-2.01290000
	C	3.20920000	6.70860000	-2.49330000
	C	3.62650000	5.67250000	-1.44570000
	C	2.45980000	4.75140000	-1.06050000
	C	1.25700000	5.58210000	-0.56580000
	C	0.84000000	6.61370000	-1.62190000
	H	1.68610000	8.22830000	-2.78880000
	H	2.30280000	8.13020000	-1.14650000
	H	4.05250000	7.36690000	-2.72220000
	H	2.94780000	6.19690000	-3.42770000
	H	3.98400000	6.17270000	-0.53900000
	H	4.45330000	5.05780000	-1.81300000
	H	2.13200000	4.18950000	-1.95030000
	N	2.87760000	3.85780000	0.00240000
	N	0.14780000	4.68900000	-0.29180000
	H	1.58130000	6.10650000	0.34800000
	H	0.00080000	7.19740000	-1.23290000
	H	0.46850000	6.07570000	-2.50090000
	C	2.92870000	2.61450000	-0.24380000
	C	3.33260000	1.61850000	0.76060000
	C	3.55500000	1.98770000	2.09760000
	C	3.93050000	1.03240000	3.02870000
	C	4.09840000	-0.29720000	2.63940000
	C	3.89120000	-0.67770000	1.31170000
	C	3.49870000	0.29030000	0.37960000
	C	4.09660000	-2.07820000	0.91310000
	N	3.99300000	-2.47540000	-0.28730000
	C	4.20950000	-3.88240000	-0.56330000
	C	2.88780000	-4.50400000	-1.06150000
	C	5.31280000	-4.03830000	-1.61790000
	C	5.51660000	-5.50430000	-2.01120000
	C	4.20560000	-6.13270000	-2.49510000
	C	3.09850000	-5.97510000	-1.44880000
	C	-0.24650000	4.57690000	0.90870000
	C	-1.35740000	3.69960000	1.30710000
	C	-1.78790000	3.68740000	2.63560000
	C	-2.00370000	2.87940000	0.37470000
	C	-3.07240000	2.07360000	0.75660000
	C	-3.50070000	2.08060000	2.09440000
	C	-2.85710000	2.87990000	3.02580000
	H	2.67070000	2.21020000	-1.23380000
	H	3.42260000	3.02620000	2.37280000
	H	4.39410000	-1.04450000	3.36830000
	H	4.35450000	-2.77040000	1.72820000

	H	4.49810000	-4.42810000	0.35010000
	H	2.56850000	-3.93660000	-1.95090000
	N	1.90390000	-4.41750000	0.00000000
	H	6.23860000	-3.60660000	-1.22680000
	H	5.03560000	-3.44540000	-2.49640000
	H	5.88770000	-6.06600000	-1.14510000
	H	6.28540000	-5.57930000	-2.78580000
	H	4.35120000	-7.19210000	-2.72610000
	H	3.89600000	-5.64720000	-3.42870000
	H	3.35010000	-6.53690000	-0.54260000
	H	2.15220000	-6.38060000	-1.81820000
	H	0.22510000	5.14420000	1.72470000
	H	-4.33500000	1.44840000	2.37010000
	C	-3.73600000	1.22710000	-0.24700000
	N	-4.78780000	0.56290000	0.00110000
	C	-5.35360000	-0.24840000	-1.05930000
	C	-5.46790000	-1.70420000	-0.56040000
	C	-6.73590000	0.29850000	-1.44390000
	C	-7.42480000	-0.58520000	-2.48770000
	C	-7.52810000	-2.03510000	-2.00260000
	C	-6.15320000	-2.58520000	-1.61300000
	H	-1.28540000	4.31470000	3.36460000
	H	-3.25820000	1.20440000	-1.23760000
	H	-4.70440000	-0.25240000	-1.95010000
	N	-4.13840000	-2.21430000	-0.28680000
	H	-6.08280000	-1.68420000	0.35440000
	H	-7.34660000	0.36060000	-0.53650000
	H	-6.61870000	1.32070000	-1.81480000
	H	-8.41750000	-0.18640000	-2.71660000
	H	-6.85200000	-0.55820000	-3.42280000
	H	-8.19760000	-2.07820000	-1.13470000
	H	-7.97680000	-2.66580000	-2.77570000
	H	-6.23710000	-3.60280000	-1.22080000
	H	-5.50290000	-2.63980000	-2.49290000
	C	-3.84340000	-2.50000000	0.91350000
	C	-2.52640000	-3.01890000	1.31140000
	C	-2.29890000	-3.38630000	2.63950000
	C	-1.49250000	-3.16340000	0.37850000
	C	-0.25890000	-3.68310000	0.75930000
	C	-0.04900000	-4.05820000	2.09660000
	C	-1.06300000	-3.90430000	3.02870000
	H	-4.57100000	-2.37820000	1.72950000
	H	-3.09360000	-3.26830000	3.36880000
	C	0.80510000	-3.83350000	-0.24540000
	H	0.91700000	-4.46240000	2.37110000
	H	0.58650000	-3.40480000	-1.23460000
	H	3.33490000	-0.02000000	-0.64580000

	H	-1.68020000	-2.86740000	-0.64710000
	H	-1.65580000	2.89400000	-0.65170000
	H	4.09480000	1.31720000	4.06140000
	H	-0.89750000	-4.18720000	4.06170000
	H	-3.18370000	2.87880000	4.05910000
**3a** (t-to-t)	C	-0.35620000	-4.19250000	-4.71690000
	H	-1.05370000	-3.54300000	-5.27020000
	C	1.00910000	-3.47200000	-4.67810000
	H	1.69030000	-4.10520000	-4.08540000
	C	1.57670000	-3.31050000	-6.09350000
	H	2.55060000	-2.81700000	-6.02690000
	H	0.91960000	-2.63440000	-6.65130000
	C	1.69230000	-4.65420000	-6.81720000
	H	2.07010000	-4.50170000	-7.83250000
	H	2.42630000	-5.28530000	-6.30080000
	C	0.34170000	-5.37730000	-6.85140000
	H	0.44300000	-6.35720000	-7.32710000
	H	-0.35960000	-4.80060000	-7.46690000
	C	-0.23400000	-5.54030000	-5.44140000
	H	0.40980000	-6.19510000	-4.84380000
	H	-1.21920000	-6.01420000	-5.47480000
	N	-0.80970000	-4.40870000	-3.35470000
	N	0.84490000	-2.16570000	-4.07060000
	C	1.39880000	-1.97450000	-2.94300000
	H	1.97480000	-2.76400000	-2.44810000
	C	1.32780000	-0.70760000	-2.20910000
	C	0.66100000	0.40460000	-2.73470000
	C	0.62920000	1.60600000	-2.03300000
	C	1.25780000	1.68890000	-0.78100000
	H	1.19980000	2.61610000	-0.23290000
	C	1.91130000	0.58990000	-0.25120000
	C	1.95090000	-0.60710000	-0.96110000
	H	2.45940000	-1.46950000	-0.54630000
	C	-0.06900000	2.75970000	-2.61210000
	H	-0.65530000	2.54800000	-3.51920000
	C	-0.72680000	4.99060000	-2.81630000
	H	-1.40020000	4.57580000	-3.58410000
	C	-1.59870000	5.76880000	-1.81140000
	H	-0.93100000	6.14950000	-1.02090000
	C	-2.29120000	6.95360000	-2.50010000
	H	-3.00190000	6.55890000	-3.23450000
	H	-2.87950000	7.49690000	-1.75510000
	C	-1.28480000	7.88330000	-3.18400000
	H	-0.64330000	8.34800000	-2.42490000
	H	-1.81190000	8.69750000	-3.68990000
	C	-0.40980000	7.11250000	-4.17710000
	H	-1.03530000	6.74220000	-4.99840000

	H	0.33710000	7.77470000	-4.62480000
	C	0.28260000	5.93030000	-3.49460000
	H	0.98430000	6.29610000	-2.73630000
	H	0.86960000	5.35490000	-4.21690000
	N	-0.00980000	3.93280000	-2.12590000
	N	-2.59750000	4.87160000	-1.26150000
	C	-2.65240000	4.75470000	0.00220000
	H	-1.96590000	5.29780000	0.66260000
	C	-3.56540000	3.82010000	0.67590000
	C	-4.42900000	2.99520000	-0.05270000
	C	-5.19180000	2.02390000	0.58870000
	C	-5.09420000	1.87650000	1.98220000
	H	-5.68690000	1.10720000	2.46010000
	C	-4.25130000	2.70010000	2.71050000
	C	-3.48920000	3.67100000	2.06180000
	H	-2.79680000	4.28700000	2.62210000
	C	-6.08570000	1.16600000	-0.20280000
	H	-6.06200000	1.33420000	-1.29030000
	C	-7.74100000	-0.44470000	-0.55340000
	H	-7.48580000	-0.28890000	-1.61440000
	C	-7.60690000	-1.95010000	-0.26030000
	H	-7.78670000	-2.09220000	0.81770000
	C	-8.64210000	-2.75870000	-1.05870000
	H	-8.39940000	-2.66960000	-2.12320000
	H	-8.53580000	-3.81610000	-0.80000000
	C	-10.07130000	-2.27380000	-0.80260000
	H	-10.77410000	-2.85200000	-1.40970000
	H	-10.33810000	-2.45510000	0.24580000
	C	-10.20320000	-0.77840000	-1.10490000
	H	-10.04520000	-0.61110000	-2.17730000
	H	-11.21490000	-0.42890000	-0.87840000
	C	-9.18130000	0.03260000	-0.30470000
	H	-9.38930000	-0.05090000	0.76770000
	H	-9.24390000	1.09550000	-0.55440000
	N	-6.85040000	0.29790000	0.31880000
	N	-6.27900000	-2.39930000	-0.63640000
	C	-5.56890000	-2.98410000	0.23630000
	H	-5.92230000	-3.12720000	1.26830000
	C	-4.23880000	-3.53840000	-0.06410000
	C	-3.66580000	-3.39290000	-1.33400000
	C	-2.42690000	-3.95740000	-1.62450000
	C	-1.73790000	-4.66170000	-0.62380000
	H	-0.77640000	-5.08970000	-0.87030000
	C	-2.28890000	-4.79780000	0.64020000
	C	-3.54040000	-4.24420000	0.91820000
	H	-3.97750000	-4.35800000	1.90480000
	C	-1.87050000	-3.82170000	-2.98030000

	H	-2.44390000	-3.17790000	-3.66260000
	C	0.35810000	-4.19260000	4.71760000
	H	1.05550000	-3.54290000	5.27070000
	C	-1.00730000	-3.47220000	4.67870000
	H	-1.68840000	-4.10560000	4.08620000
	C	-1.57490000	-3.31040000	6.09410000
	H	-0.91770000	-2.63410000	6.65170000
	H	-2.54870000	-2.81690000	6.02740000
	C	-1.69030000	-4.65390000	6.81810000
	H	-2.06800000	-4.50110000	7.83340000
	H	-2.42440000	-5.28510000	6.30200000
	C	-0.33980000	-5.37700000	6.85240000
	H	-0.44100000	-6.35680000	7.32830000
	H	0.36150000	-4.80010000	7.46780000
	C	0.23590000	-5.54020000	5.44240000
	H	-0.40790000	-6.19520000	4.84500000
	H	1.22110000	-6.01410000	5.47590000
	N	0.81150000	-4.40900000	3.35540000
	N	-0.84340000	-2.16610000	4.07090000
	C	-1.39740000	-1.97510000	2.94340000
	H	-1.97330000	-2.76490000	2.44860000
	C	-1.32700000	-0.70820000	2.20940000
	C	-0.66080000	0.40430000	2.73500000
	C	-0.62960000	1.60570000	2.03340000
	C	-1.25840000	1.68840000	0.78140000
	H	-1.20090000	2.61560000	0.23340000
	C	-1.91140000	0.58910000	0.25160000
	C	-1.95020000	-0.60810000	0.96140000
	H	-2.45820000	-1.47070000	0.54660000
	C	0.06810000	2.75970000	2.61240000
	H	0.65430000	2.54820000	3.51980000
	C	0.72550000	4.99070000	2.81650000
	H	1.39910000	4.57610000	3.58430000
	C	1.59710000	5.76930000	1.81170000
	H	0.92920000	6.14980000	1.02120000
	C	2.28910000	6.95430000	2.50040000
	H	3.00010000	6.55990000	3.23480000
	H	2.87720000	7.49790000	1.75540000
	C	1.28240000	7.88370000	3.18440000
	H	1.80930000	8.69800000	3.69040000
	H	0.64080000	8.34820000	2.42530000
	C	0.40780000	7.11250000	4.17740000
	H	-0.33930000	7.77430000	4.62530000
	H	1.03350000	6.74230000	4.99870000
	C	-0.28420000	5.93000000	3.49500000
	H	-0.98620000	6.29550000	2.73670000
	H	-0.87080000	5.35430000	4.21730000

	N	0.00880000	3.93270000	2.12600000
	N	2.59610000	4.87240000	1.26160000
	C	2.65100000	4.75570000	-0.00200000
	H	1.96440000	5.29880000	-0.66240000
	C	3.56420000	3.82150000	-0.67590000
	C	4.42800000	2.99660000	0.05250000
	C	5.19070000	2.02540000	-0.58910000
	C	5.09280000	1.87800000	-1.98260000
	H	5.68540000	1.10870000	-2.46060000
	C	4.24980000	2.70150000	-2.71070000
	C	3.48770000	3.67240000	-2.06180000
	H	2.79510000	4.28840000	-2.62180000
	C	6.08490000	1.16750000	0.20220000
	H	6.06120000	1.33540000	1.28970000
	C	7.74070000	-0.44280000	0.55240000
	H	7.48550000	-0.28730000	1.61340000
	C	7.60700000	-1.94820000	0.25900000
	H	7.78650000	-2.09000000	-0.81910000
	C	8.64270000	-2.75660000	1.05690000
	H	8.53670000	-3.81400000	0.79780000
	H	8.40010000	-2.66800000	2.12150000
	C	10.07160000	-2.27100000	0.80080000
	H	10.77480000	-2.84920000	1.40750000
	H	10.33840000	-2.45190000	-0.24780000
	C	10.20300000	-0.77570000	1.10350000
	H	11.21450000	-0.42570000	0.87720000
	H	10.04500000	-0.60880000	2.17600000
	C	9.18080000	0.03510000	0.30370000
	H	9.38870000	-0.04800000	-0.76880000
	H	9.24290000	1.09800000	0.55370000
	N	6.84970000	0.29970000	-0.31960000
	N	6.27930000	-2.39770000	0.63540000
	C	5.56940000	-2.98340000	-0.23690000
	H	5.92280000	-3.12690000	-1.26890000
	C	4.23960000	-3.53810000	0.06400000
	C	3.66690000	-3.39260000	1.33400000
	C	2.42820000	-3.95740000	1.62480000
	C	1.73900000	-4.66180000	0.62430000
	H	0.77770000	-5.09000000	0.87100000
	C	2.28970000	-4.79790000	-0.63980000
	C	3.54110000	-4.24410000	-0.91820000
	H	3.97800000	-4.35800000	-1.90480000
	C	1.87200000	-3.82170000	2.98070000
	H	2.44530000	-3.17760000	3.66280000
	H	0.17890000	0.30860000	-3.70090000
	H	-4.21880000	-2.84360000	-2.08710000
	H	-0.17870000	0.30850000	3.70120000

	H	4.46540000	3.11260000	1.12960000
	H	4.21990000	-2.84320000	2.08690000
	H	-4.46620000	3.11130000	-1.12980000
	H	2.38290000	0.66000000	0.72060000
	H	-2.38310000	0.65900000	-0.72010000
	H	4.16690000	2.57910000	-3.78410000
	H	1.74470000	-5.32150000	-1.41640000
	H	-4.16870000	2.57770000	3.78390000
	H	-1.74390000	-5.32140000	1.41690000
**3a** (h-to-h)	N	0.99190000	4.46900000	1.77940000
	C	-0.83710000	4.12810000	3.28780000
	C	-3.39720000	3.17720000	3.91030000
	H	-4.39620000	2.81340000	4.11400000
	C	0.46040000	4.71090000	2.90430000
	H	0.91640000	5.40130000	3.63020000
	C	-1.50230000	4.57780000	4.43050000
	H	-1.02840000	5.31540000	5.06970000
	C	-3.38610000	1.77880000	1.82690000
	H	-2.72570000	1.33870000	1.06930000
	C	-2.77720000	4.09990000	4.73950000
	C	-1.45510000	3.16990000	2.47560000
	C	-2.73230000	2.70120000	2.76950000
	N	3.65810000	3.23660000	1.83950000
	C	3.04730000	6.88730000	-0.29360000
	H	2.71860000	7.60730000	-1.04940000
	H	3.59640000	7.45630000	0.46720000
	C	3.18560000	4.13280000	0.79470000
	H	2.61740000	3.58480000	0.03270000
	C	2.20750000	5.16130000	1.39230000
	H	2.69280000	5.64940000	2.25270000
	C	1.82720000	6.21820000	0.33940000
	H	1.17490000	6.95850000	0.81170000
	H	1.23220000	5.72040000	-0.43120000
	C	4.39380000	4.80560000	0.13140000
	H	5.01250000	4.03220000	-0.32820000
	H	4.99830000	5.27860000	0.91340000
	C	3.97410000	5.84050000	-0.91430000
	H	4.86190000	6.31550000	-1.34200000
	H	3.45650000	5.33900000	-1.73910000
	N	-4.36910000	-1.37810000	1.77960000
	C	-3.15640000	-2.78650000	3.29080000
	C	-1.04890000	-4.52300000	3.91610000
	H	-0.23310000	-5.20430000	4.12130000
	C	-4.31090000	-1.95610000	2.90620000
	H	-5.13630000	-1.90530000	3.63270000
	C	-3.20980000	-3.58280000	4.43690000
	H	-4.08410000	-3.53940000	5.07800000

	C	0.15100000	-3.81980000	1.82710000
	H	0.20100000	-3.02640000	1.07060000
	C	-2.15660000	-4.44540000	4.74720000
	C	-2.01920000	-2.84490000	2.47640000
	C	-0.97260000	-3.71360000	2.77200000
	N	-4.63290000	1.54540000	1.84080000
	C	-7.49030000	-0.80460000	-0.29480000
	H	-7.94960000	-1.44780000	-1.05180000
	H	-8.25760000	-0.61470000	0.46630000
	C	-5.17410000	0.68950000	0.79600000
	H	-4.41630000	0.47090000	0.03320000
	C	-5.57680000	-0.67210000	1.39210000
	H	-6.24310000	-0.49690000	2.25190000
	C	-6.30070000	-1.52830000	0.33710000
	H	-6.61530000	-2.46440000	0.80720000
	H	-5.57140000	-1.79220000	-0.43350000
	C	-6.36040000	1.40130000	0.13450000
	H	-5.99950000	2.32460000	-0.32280000
	H	-7.07230000	1.68670000	0.91710000
	C	-7.04670000	0.52260000	-0.91290000
	H	-7.90150000	1.05490000	-1.34040000
	H	-6.35310000	0.32630000	-1.73750000
	N	3.37810000	-3.09570000	1.77530000
	C	3.99990000	-1.34250000	3.28400000
	C	4.45570000	1.34980000	3.90860000
	H	4.64040000	2.39670000	4.11290000
	C	3.85570000	-2.75730000	2.89950000
	H	4.22890000	-3.49790000	3.62330000
	C	4.72360000	-0.99200000	4.42580000
	H	5.12680000	-1.77170000	5.06370000
	C	3.23620000	2.04040000	1.82690000
	H	2.52290000	1.68850000	1.07120000
	C	4.94660000	0.35090000	4.73600000
	C	3.47720000	-0.32760000	2.47370000
	C	3.70940000	1.01280000	2.76860000
	N	0.97600000	-4.78350000	1.83950000
	C	4.43640000	-6.08440000	-0.30410000
	H	5.22080000	-6.15930000	-1.06360000
	H	4.65850000	-6.84440000	0.45560000
	C	1.98660000	-4.82570000	0.79340000
	H	1.79580000	-4.06090000	0.03010000
	C	3.36850000	-4.49410000	1.38660000
	H	3.55140000	-5.15930000	2.24560000
	C	4.46940000	-4.69290000	0.32870000
	H	5.43870000	-4.49730000	0.79620000
	H	4.33140000	-3.92900000	-0.44140000
	C	1.96240000	-6.20930000	0.13250000

	H	0.98140000	-6.35900000	-0.32260000
	H	2.07330000	-6.96850000	0.91470000
	C	3.06350000	-6.36420000	-0.91810000
	H	3.02860000	-7.37080000	-1.34510000
	H	2.88400000	-5.66570000	-1.74240000
	N	-0.91130000	4.48400000	-1.78020000
	C	0.91220000	4.11100000	-3.28800000
	C	3.45580000	3.11590000	-3.90960000
	H	4.44870000	2.73530000	-4.11260000
	C	-0.37490000	4.71660000	-2.90490000
	H	-0.81730000	5.41650000	-3.63000000
	C	1.58620000	4.55020000	-4.42960000
	H	1.12580000	5.29670000	-5.06840000
	C	3.41880000	1.71660000	-1.82710000
	H	2.74910000	1.28550000	-1.07230000
	C	2.85280000	4.05020000	-4.73830000
	C	1.51280000	3.14140000	-2.47610000
	C	2.78190000	2.65060000	-2.76970000
	N	-3.60000000	3.30260000	-1.83740000
	C	-2.91770000	6.94310000	0.29270000
	H	-2.57450000	7.65720000	1.04770000
	H	-3.45610000	7.52230000	-0.46810000
	C	-3.11040000	4.19100000	-0.79380000
	H	-2.55210000	3.63320000	-0.03160000
	C	-2.11310000	5.20000000	-1.39290000
	H	-2.58890000	5.69710000	-2.25340000
	C	-1.71120000	6.24970000	-0.34080000
	H	-1.04510000	6.97690000	-0.81410000
	H	-1.12540000	5.74100000	0.42950000
	C	-4.30510000	4.88790000	-0.13090000
	H	-4.93890000	4.12660000	0.32830000
	H	-4.90010000	5.37240000	-0.91320000
	C	-3.86480000	5.91490000	0.91420000
	H	-4.74290000	6.40740000	1.34230000
	H	-3.35680000	5.40340000	1.73890000
	N	4.34220000	-1.45500000	-1.77890000
	C	3.10520000	-2.84210000	-3.28990000
	C	0.96730000	-4.54040000	-3.91570000
	H	0.13960000	-5.20720000	-4.12080000
	C	4.27490000	-2.03360000	-2.90470000
	H	5.10230000	-1.99970000	-3.62970000
	C	3.14560000	-3.64070000	-4.43480000
	H	4.02130000	-3.61420000	-5.07480000
	C	-0.22200000	-3.81430000	-1.82850000
	H	-0.25840000	-3.02060000	-1.07190000
	C	2.07700000	-4.48400000	-4.74560000
	C	1.96600000	-2.87810000	-2.47710000

	C	0.90430000	-3.72850000	-2.77250000
	N	4.66190000	1.46350000	-1.83860000
	C	7.47150000	-0.94450000	0.29860000
	H	7.91600000	-1.59730000	1.05630000
	H	8.24410000	-0.77050000	-0.46090000
	C	5.18660000	0.59680000	-0.79420000
	H	4.42440000	0.39300000	-0.03180000
	C	5.56270000	-0.77230000	-1.39070000
	H	6.23280000	-0.60990000	-2.25000000
	C	6.26860000	-1.64320000	-0.33540000
	H	6.56570000	-2.58490000	-0.80590000
	H	5.53320000	-1.89400000	0.43380000
	C	6.38690000	1.28420000	-0.13140000
	H	6.04450000	2.21450000	0.32620000
	H	7.10480000	1.55560000	-0.91340000
	C	7.05460000	0.39150000	0.91640000
	H	7.91990000	0.90620000	1.34420000
	H	6.35670000	0.20980000	1.74090000
	N	-3.43370000	-3.03280000	-1.77760000
	C	-4.02420000	-1.26810000	-3.28560000
	C	-4.43100000	1.43250000	-3.90830000
	H	-4.59630000	2.48270000	-4.11220000
	C	-3.90600000	-2.68540000	-2.90130000
	H	-4.29260000	-3.41890000	-3.62520000
	C	-4.74140000	-0.90380000	-4.42710000
	H	-5.15910000	-1.67550000	-5.06530000
	C	-3.19740000	2.09950000	-1.82720000
	H	-2.48780000	1.73460000	-1.07400000
	C	-4.94000000	0.44320000	-4.73640000
	C	-3.48250000	-0.26330000	-2.47500000
	C	-3.69060000	1.08130000	-2.76880000
	N	-1.06300000	-4.76400000	-1.84030000
	C	-4.54830000	-6.00160000	0.30080000
	H	-5.33500000	-6.06200000	1.05910000
	H	-4.78270000	-6.75810000	-0.45870000
	C	-2.07450000	-4.78730000	-0.79460000
	H	-1.87040000	-4.02490000	-0.03210000
	C	-3.44960000	-4.43140000	-1.38930000
	H	-3.64360000	-5.09290000	-2.24880000
	C	-4.55560000	-4.61030000	-0.33320000
	H	-5.52040000	-4.39910000	-0.80320000
	H	-4.40700000	-3.84810000	0.43650000
	C	-2.07610000	-6.17070000	-0.13260000
	H	-1.09850000	-6.33820000	0.32360000
	H	-2.19980000	-6.92810000	-0.91470000
	C	-3.18140000	-6.30490000	0.91680000
	H	-3.16530000	-7.31160000	1.34470000

	H	-2.99050000	-5.60910000	1.74090000
	H	1.95300000	-2.25980000	-1.58740000
	H	-2.93520000	-0.56110000	-1.58850000
	H	0.98230000	2.81700000	-1.58870000
	H	2.92540000	-0.61480000	1.58660000
	H	-0.93070000	2.83650000	1.58800000
	H	-1.99580000	-2.22840000	1.58560000
	H	5.51530000	0.61200000	5.62090000
	H	-3.28760000	4.46050000	5.62490000
	H	-2.21110000	-5.06410000	5.63540000
	H	-5.50390000	0.71520000	-5.62100000
	H	3.37000000	4.40260000	-5.62310000
	H	2.12130000	-5.10460000	-5.63310000
**3b** (m)	C	7.67460000	-1.95990000	-2.93410000
	C	6.90380000	-3.20940000	-3.37760000
	C	5.80870000	-3.58200000	-2.37180000
	C	4.85640000	-2.40450000	-2.12920000
	C	5.65670000	-1.17070000	-1.63910000
	C	6.72780000	-0.78610000	-2.66090000
	H	8.41020000	-1.67910000	-3.69330000
	H	8.23880000	-2.18960000	-2.02190000
	H	7.58850000	-4.05200000	-3.51030000
	H	6.44550000	-3.02090000	-4.35610000
	H	6.25210000	-3.87380000	-1.41370000
	H	5.23070000	-4.43970000	-2.72780000
	H	4.36540000	-2.13480000	-3.07800000
	N	3.88320000	-2.72020000	-1.10430000
	N	4.72530000	-0.07970000	-1.41330000
	H	6.14260000	-1.47190000	-0.69740000
	H	7.28050000	0.08180000	-2.29020000
	H	6.23130000	-0.47270000	-3.58580000
	C	2.66090000	-2.80460000	-1.42790000
	C	1.59450000	-2.94260000	-0.42110000
	C	1.86380000	-2.61440000	0.91710000
	C	0.83760000	-2.65610000	1.84280000
	C	-0.44800000	-3.05390000	1.48350000
	C	-0.71090000	-3.40360000	0.15610000
	C	0.31070000	-3.32310000	-0.79710000
	C	-2.07410000	-3.81120000	-0.21570000
	N	-2.42250000	-4.05700000	-1.41130000
	C	-3.83160000	-4.31980000	-1.64350000
	C	-4.50040000	-3.00960000	-2.13360000
	C	-4.02790000	-5.43700000	-2.66930000
	C	-5.51600000	-5.67180000	-2.95020000
	C	-6.21330000	-4.37960000	-3.39280000
	C	-5.99470000	-3.24730000	-2.38300000
	C	4.34150000	0.10880000	-0.21810000

	C	3.30810000	1.08880000	0.14920000
	C	2.88000000	1.15420000	1.47810000
	C	2.72300000	1.92380000	-0.80940000
	C	1.75330000	2.84900000	-0.43770000
	C	1.34100000	2.93120000	0.90180000
	C	1.89460000	2.07230000	1.83330000
	H	2.32540000	-2.72770000	-2.47240000
	H	2.86470000	-2.31410000	1.19060000
	H	-1.23950000	-3.07290000	2.22200000
	H	-2.79000000	-3.86520000	0.61560000
	H	-4.33900000	-4.59340000	-0.70470000
	H	-4.01760000	-2.71670000	-3.07990000
	N	-4.29400000	-2.00940000	-1.10670000
	H	-3.55290000	-6.34980000	-2.29910000
	H	-3.50410000	-5.16000000	-3.59090000
	H	-6.00070000	-6.04950000	-2.04150000
	H	-5.63550000	-6.44710000	-3.71240000
	H	-7.28450000	-4.55320000	-3.53060000
	H	-5.81750000	-4.07340000	-4.36900000
	H	-6.47310000	-3.48870000	-1.42770000
	H	-6.44850000	-2.31780000	-2.73860000
	H	4.74780000	-0.47880000	0.61640000
	H	0.58290000	3.65130000	1.17250000
	C	1.09590000	3.69400000	-1.44930000
	N	0.41620000	4.71540000	-1.13250000
	C	-0.35230000	5.38910000	-2.15880000
	C	-1.81770000	5.46530000	-1.65870000
	C	0.18620000	6.80150000	-2.41770000
	C	-0.69410000	7.55340000	-3.42250000
	C	-2.15770000	7.59690000	-2.96680000
	C	-2.69600000	6.19130000	-2.67890000
	H	3.29580000	0.48530000	2.22100000
	H	1.18800000	3.35220000	-2.49070000
	H	-0.34560000	4.82040000	-3.10260000
	N	-2.29260000	4.11390000	-1.42080000
	H	-1.79420000	6.04300000	-0.72090000
	H	0.22380000	7.33970000	-1.46440000
	H	1.21520000	6.72890000	-2.78140000
	H	-0.31020000	8.56740000	-3.56670000
	H	-0.63520000	7.05450000	-4.39750000
	H	-2.23400000	8.20680000	-2.05830000
	H	-2.77610000	8.08700000	-3.72430000
	H	-3.72100000	6.23700000	-2.30020000
	H	-2.72550000	5.59750000	-3.59910000
	C	-2.25770000	3.69490000	-0.22310000
	C	-2.58950000	2.31230000	0.15310000
	C	-2.43030000	1.91550000	1.48370000

	C	-3.02370000	1.38370000	-0.79960000
	C	-3.34260000	0.08410000	-0.42060000
	C	-3.20600000	-0.30740000	0.92060000
	C	-2.73490000	0.60550000	1.84620000
	H	-1.94830000	4.34560000	0.60590000
	H	-2.05710000	2.61330000	2.22260000
	C	-3.75240000	-0.90960000	-1.42760000
	H	-3.45250000	-1.32200000	1.19750000
	H	-3.51000000	-0.65980000	-2.47090000
	Br	1.18650000	-2.11620000	3.64660000
	Br	1.26310000	2.12370000	3.64040000
	Br	-2.46170000	0.04210000	3.65580000
	H	0.07550000	-3.56430000	-1.82670000
	H	-3.10570000	1.70360000	-1.83130000
	H	3.04510000	1.83110000	-1.83960000
**3b** (t-to-t)	C	-1.17180000	-4.32170000	-4.06840000
	H	-1.59080000	-3.56730000	-4.75310000
	C	0.31370000	-3.98270000	-3.84530000
	H	0.70030000	-4.68880000	-3.09660000
	C	1.11650000	-4.13890000	-5.14460000
	H	2.16560000	-3.91960000	-4.92990000
	H	0.77240000	-3.38220000	-5.85810000
	C	0.96120000	-5.53870000	-5.74230000
	H	1.52550000	-5.61040000	-6.67670000
	H	1.39200000	-6.27900000	-5.05670000
	C	-0.51520000	-5.86970000	-5.97920000
	H	-0.62440000	-6.88620000	-6.36860000
	H	-0.91780000	-5.19430000	-6.74420000
	C	-1.32320000	-5.72030000	-4.68760000
	H	-0.98980000	-6.45700000	-3.94870000
	H	-2.38510000	-5.91220000	-4.86620000
	N	-1.85550000	-4.31320000	-2.78810000
	N	0.42610000	-2.61110000	-3.38430000
	C	0.99200000	-2.40490000	-2.26760000
	H	1.37610000	-3.22660000	-1.65110000
	C	1.17340000	-1.05540000	-1.71260000
	C	0.68420000	0.07390000	-2.37620000
	C	0.81530000	1.33980000	-1.81470000
	C	1.45210000	1.48770000	-0.57110000
	H	1.50300000	2.46070000	-0.11170000
	C	1.95680000	0.36540000	0.06040000
	C	1.82360000	-0.90670000	-0.48660000
	H	2.20860000	-1.77140000	0.03570000
	C	0.22240000	2.48840000	-2.51710000
	H	-0.32620000	2.23170000	-3.43350000
	Br	2.82440000	0.54030000	1.76340000
	C	-0.30800000	4.72510000	-2.92830000

	H	-0.95220000	4.28410000	-3.70520000
	C	-1.19950000	5.61470000	-2.04020000
	H	-0.57250000	5.99920000	-1.21910000
	C	-1.76550000	6.79820000	-2.84100000
	H	-2.45750000	6.40210000	-3.59220000
	H	-2.35880000	7.42230000	-2.16670000
	C	-0.66570000	7.61730000	-3.52000000
	H	-0.03600000	8.09200000	-2.75730000
	H	-1.11210000	8.42630000	-4.10530000
	C	0.20570000	6.72700000	-4.41020000
	H	-0.40090000	6.33380000	-5.23480000
	H	1.01460000	7.30770000	-4.86270000
	C	0.78880000	5.56310000	-3.60590000
	H	1.45790000	5.95650000	-2.83240000
	H	1.39030000	4.90830000	-4.24280000
	N	0.31280000	3.68960000	-2.11450000
	N	-2.30750000	4.82890000	-1.53330000
	C	-2.48760000	4.78560000	-0.27770000
	H	-1.81070000	5.28270000	0.42550000
	C	-3.58510000	4.01050000	0.32420000
	C	-4.53950000	3.37330000	-0.47300000
	C	-5.53260000	2.58890000	0.10340000
	C	-5.57450000	2.42040000	1.49640000
	H	-6.34590000	1.79860000	1.92850000
	C	-4.61930000	3.05130000	2.27250000
	C	-3.63060000	3.85230000	1.71040000
	H	-2.87530000	4.30860000	2.33080000
	C	-6.52110000	1.92930000	-0.76240000
	H	-6.32370000	2.00330000	-1.84140000
	Br	-4.62070000	2.79540000	4.17490000
	C	-8.45180000	0.71700000	-1.25570000
	H	-8.05700000	0.76980000	-2.28330000
	C	-8.62440000	-0.77210000	-0.90070000
	H	-8.96720000	-0.82040000	0.14540000
	C	-9.67070000	-1.43100000	-1.81090000
	H	-9.28340000	-1.43150000	-2.83560000
	H	-9.78500000	-2.47820000	-1.51700000
	C	-11.01350000	-0.69710000	-1.75330000
	H	-11.72530000	-1.17060000	-2.43570000
	H	-11.43670000	-0.78820000	-0.74540000
	C	-10.84600000	0.78620000	-2.09760000
	H	-10.53520000	0.88130000	-3.14520000
	H	-11.80180000	1.31020000	-2.00590000
	C	-9.80150000	1.44710000	-1.19390000
	H	-10.14550000	1.44730000	-0.15380000
	H	-9.64760000	2.49280000	-1.47470000
	N	-7.53510000	1.31980000	-0.30730000

	N	-7.35180000	-1.44880000	-1.06810000
	C	-6.82560000	-1.99750000	-0.05420000
	H	-7.30210000	-1.96710000	0.93610000
	C	-5.56410000	-2.75380000	-0.13420000
	C	-4.79350000	-2.76030000	-1.30260000
	C	-3.64970000	-3.54690000	-1.39080000
	C	-3.26360000	-4.34780000	-0.30160000
	H	-2.38440000	-4.97020000	-0.39670000
	C	-4.02660000	-4.32140000	0.85150000
	C	-5.16510000	-3.52560000	0.95820000
	H	-5.74900000	-3.52620000	1.86990000
	C	-2.86490000	-3.56230000	-2.63860000
	H	-3.22240000	-2.89380000	-3.43370000
	Br	-3.53870000	-5.43300000	2.33770000
	C	1.17210000	-4.32090000	4.06870000
	H	1.59110000	-3.56640000	4.75340000
	C	-0.31340000	-3.98220000	3.84560000
	H	-0.69990000	-4.68850000	3.09700000
	C	-1.11610000	-4.13840000	5.14490000
	H	-0.77220000	-3.38160000	5.85840000
	H	-2.16520000	-3.91930000	4.93030000
	C	-0.96070000	-5.53810000	5.74280000
	H	-1.52490000	-5.60980000	6.67720000
	H	-1.39140000	-6.27850000	5.05720000
	C	0.51580000	-5.86890000	5.97970000
	H	0.62520000	-6.88530000	6.36910000
	H	0.91830000	-5.19340000	6.74460000
	C	1.32370000	-5.71950000	4.68800000
	H	0.99050000	-6.45630000	3.94920000
	H	2.38570000	-5.91120000	4.86660000
	N	1.85590000	-4.31250000	2.78840000
	N	-0.42610000	-2.61070000	3.38450000
	C	-0.99190000	-2.40470000	2.26770000
	H	-1.37570000	-3.22650000	1.65130000
	C	-1.17350000	-1.05520000	1.71260000
	C	-0.68450000	0.07420000	2.37610000
	C	-0.81570000	1.34010000	1.81450000
	C	-1.45250000	1.48770000	0.57080000
	H	-1.50350000	2.46060000	0.11130000
	C	-1.95700000	0.36530000	-0.06060000
	C	-1.82360000	-0.90680000	0.48650000
	H	-2.20840000	-1.77160000	-0.03570000
	C	-0.22300000	2.48870000	2.51680000
	H	0.32560000	2.23220000	3.43320000
	Br	-2.82450000	0.53980000	-1.76370000
	C	0.30760000	4.72550000	2.92760000
	H	0.95170000	4.28450000	3.70460000

	C	1.19910000	5.61490000	2.03950000
	H	0.57220000	5.99940000	1.21830000
	C	1.76510000	6.79850000	2.84020000
	H	2.45710000	6.40250000	3.59140000
	H	2.35840000	7.42260000	2.16580000
	C	0.66530000	7.61770000	3.51910000
	H	1.11170000	8.42680000	4.10430000
	H	0.03560000	8.09230000	2.75640000
	C	-0.20610000	6.72750000	4.40940000
	H	-1.01500000	7.30820000	4.86180000
	H	0.40050000	6.33430000	5.23400000
	C	-0.78920000	5.56350000	3.60510000
	H	-1.45830000	5.95690000	2.83160000
	H	-1.39070000	4.90870000	4.24210000
	N	-0.31320000	3.68990000	2.11390000
	N	2.30720000	4.82920000	1.53270000
	C	2.48720000	4.78560000	0.27710000
	H	1.81020000	5.28230000	-0.42620000
	C	3.58470000	4.01030000	-0.32470000
	C	4.53930000	3.37360000	0.47270000
	C	5.53250000	2.58910000	-0.10360000
	C	5.57420000	2.42010000	-1.49650000
	H	6.34570000	1.79830000	-1.92850000
	C	4.61880000	3.05060000	-2.27270000
	C	3.63010000	3.85160000	-1.71070000
	H	2.87450000	4.30740000	-2.33120000
	C	6.52110000	1.93000000	0.76240000
	H	6.32380000	2.00420000	1.84130000
	Br	4.61990000	2.79380000	-4.17500000
	C	8.45200000	0.71790000	1.25580000
	H	8.05720000	0.77090000	2.28350000
	C	8.62470000	-0.77110000	0.90110000
	H	8.96750000	-0.81960000	-0.14500000
	C	9.67100000	-1.42980000	1.81150000
	H	9.78540000	-2.47710000	1.51780000
	H	9.28360000	-1.43030000	2.83610000
	C	11.01380000	-0.69590000	1.75380000
	H	11.72550000	-1.16920000	2.43640000
	H	11.43700000	-0.78710000	0.74600000
	C	10.84610000	0.78740000	2.09780000
	H	11.80190000	1.31150000	2.00610000
	H	10.53530000	0.88270000	3.14540000
	C	9.80170000	1.44810000	1.19400000
	H	10.14560000	1.44820000	0.15390000
	H	9.64770000	2.49390000	1.47460000
	N	7.53520000	1.32050000	0.30740000
	N	7.35210000	-1.44800000	1.06860000

	C	6.82600000	-1.99680000	0.05470000
	H	7.30250000	-1.96640000	-0.93560000
	C	5.56450000	-2.75310000	0.13460000
	C	4.79380000	-2.75950000	1.30290000
	C	3.65010000	-3.54610000	1.39120000
	C	3.26400000	-4.34710000	0.30200000
	H	2.38490000	-4.96960000	0.39710000
	C	4.02710000	-4.32080000	-0.85110000
	C	5.16570000	-3.52500000	-0.95770000
	H	5.74960000	-3.52560000	-1.86940000
	C	2.86530000	-3.56160000	2.63890000
	H	3.22270000	-2.89290000	3.43400000
	Br	3.53940000	-5.43250000	-2.33720000
	H	0.17470000	-0.06590000	-3.32090000
	H	-5.11620000	-2.14950000	-2.13590000
	H	-0.17500000	-0.06550000	3.32080000
	H	4.47580000	3.49110000	1.54670000
	H	5.11650000	-2.14860000	2.13620000
	H	-4.47590000	3.49050000	-1.54710000
**3b** (h-to-h)	N	3.42600000	3.07670000	-1.72480000
	C	4.10790000	1.31230000	-3.18740000
	C	4.58710000	-1.39250000	-3.75730000
	H	4.78660000	-2.43870000	-3.94240000
	C	3.95630000	2.73320000	-2.82290000
	H	4.37160000	3.46570000	-3.53060000
	C	4.89610000	0.95600000	-4.28300000
	H	5.34440000	1.71920000	-4.90690000
	C	3.26020000	-2.05660000	-1.74350000
	H	2.49830000	-1.70660000	-1.03600000
	C	5.12080000	-0.39360000	-4.55230000
	C	3.53220000	0.30910000	-2.39950000
	C	3.77800000	-1.03340000	-2.66830000
	N	1.02870000	4.76720000	-1.81330000
	C	4.46350000	6.05750000	0.37530000
	H	5.23900000	6.12840000	1.14390000
	H	4.69410000	6.82150000	-0.37740000
	C	2.02630000	4.80520000	-0.75510000
	H	1.82660000	4.03590000	0.00150000
	C	3.41400000	4.47570000	-1.33460000
	H	3.60710000	5.14180000	-2.18990000
	C	4.50440000	4.66930000	-0.26460000
	H	5.47810000	4.47760000	-0.72450000
	H	4.36060000	3.90080000	0.49980000
	C	1.99390000	6.18570000	-0.08770000
	H	1.00770000	6.33390000	0.35670000
	H	2.11380000	6.94870000	-0.86450000
	C	3.08380000	6.33390000	0.97550000

	H	3.04450000	7.33730000	1.40830000
	H	2.89590000	5.63090000	1.79410000
	N	1.02180000	-4.44900000	-1.75230000
	C	-0.78570000	-4.10470000	-3.28210000
	C	-3.34390000	-3.15330000	-3.93230000
	H	-4.34120000	-2.79760000	-4.14980000
	C	0.50480000	-4.69640000	-2.88230000
	H	0.96060000	-5.39780000	-3.59650000
	C	-1.43970000	-4.57470000	-4.42250000
	H	-0.97460000	-5.32370000	-5.05120000
	C	-3.34680000	-1.73570000	-1.87210000
	H	-2.69860000	-1.28750000	-1.10880000
	C	-2.70980000	-4.08870000	-4.73080000
	C	-1.40360000	-3.13230000	-2.48730000
	C	-2.67740000	-2.66640000	-2.79750000
	N	3.70220000	-3.24440000	-1.71980000
	C	2.98740000	-6.90430000	0.36320000
	H	2.62590000	-7.62800000	1.09990000
	H	3.56100000	-7.47100000	-0.38080000
	C	3.18650000	-4.14310000	-0.69780000
	H	2.59490000	-3.59370000	0.04480000
	C	2.22040000	-5.15710000	-1.33720000
	H	2.72630000	-5.64030000	-2.18750000
	C	1.79610000	-6.22170000	-0.30870000
	H	1.15690000	-6.95290000	-0.81180000
	H	1.17670000	-5.72740000	0.44440000
	C	4.36380000	-4.83000000	0.00460000
	H	4.97220000	-4.06450000	0.49060000
	H	4.99230000	-5.30370000	-0.75750000
	C	3.89620000	-5.86790000	1.02660000
	H	4.76260000	-6.35080000	1.48700000
	H	3.34920000	-5.36790000	1.83360000
	N	-4.34320000	1.40760000	-1.85720000
	C	-3.09560000	2.81630000	-3.33350000
	C	-0.96230000	4.54780000	-3.90330000
	H	-0.14450000	5.22930000	-4.09160000
	C	-4.26530000	1.99100000	-2.97910000
	H	-5.07620000	1.95040000	-3.72100000
	C	-3.13540000	3.62770000	-4.46870000
	H	-3.99580000	3.60930000	-5.12590000
	C	0.20010000	3.80800000	-1.81450000
	H	0.23440000	3.01020000	-1.06260000
	C	-2.06520000	4.48050000	-4.73640000
	C	-1.96970000	2.85180000	-2.50300000
	C	-0.91410000	3.71660000	-2.77360000
	N	-4.59390000	-1.51180000	-1.90870000
	C	-7.50920000	0.83440000	0.14980000

	H	-7.98900000	1.47810000	0.89320000
	H	-8.25610000	0.64150000	-0.63010000
	C	-5.16390000	-0.65450000	-0.88050000
	H	-4.42400000	-0.42750000	-0.10330000
	C	-5.55880000	0.70250000	-1.49210000
	H	-6.20690000	0.52380000	-2.36430000
	C	-6.30580000	1.55950000	-0.45350000
	H	-6.61010000	2.49560000	-0.93010000
	H	-5.59400000	1.82280000	0.33380000
	C	-6.36090000	-1.37040000	-0.24310000
	H	-6.00530000	-2.28840000	0.22920000
	H	-7.05170000	-1.66550000	-1.04050000
	C	-7.07920000	-0.49050000	0.78220000
	H	-7.94370000	-1.02440000	1.18640000
	H	-6.41130000	-0.29070000	1.62660000
	N	4.33190000	1.44500000	1.85800000
	C	3.07100000	2.84000000	3.33570000
	C	0.92270000	4.55290000	3.90480000
	H	0.09900000	5.22730000	4.09290000
	C	4.24800000	2.02520000	2.98120000
	H	5.05840000	1.98970000	3.72390000
	C	3.10290000	3.65060000	4.47170000
	H	3.96270000	3.63880000	5.12980000
	C	-0.23180000	3.80450000	1.81480000
	H	-0.25980000	3.00580000	1.06340000
	C	2.02520000	4.49420000	4.73910000
	C	1.94570000	2.86660000	2.50420000
	C	0.88260000	3.72220000	2.77450000
	N	4.60650000	-1.47200000	1.90910000
	C	7.50300000	0.89840000	-0.14820000
	H	7.97780000	1.54610000	-0.89120000
	H	8.25110000	0.71120000	0.63200000
	C	5.16930000	-0.60990000	0.88100000
	H	4.42730000	-0.38890000	0.10400000
	C	5.55330000	0.75000000	1.49280000
	H	6.20260000	0.57630000	2.36510000
	C	6.29340000	1.61340000	0.45480000
	H	6.58970000	2.55190000	0.93180000
	H	5.57980000	1.87100000	-0.33280000
	C	6.37210000	-1.31590000	0.24340000
	H	6.02400000	-2.23630000	-0.22970000
	H	7.06480000	-1.60600000	1.04100000
	C	7.08390000	-0.42970000	-0.78110000
	H	7.95290000	-0.95670000	-1.18500000
	H	6.41480000	-0.23510000	-1.62580000
	N	-3.45160000	3.04820000	1.72270000
	C	-4.11680000	1.27860000	3.18670000

	C	-4.57440000	-1.43000000	3.75660000
	H	-4.76550000	-2.47770000	3.94170000
	C	-3.97700000	2.70070000	2.82180000
	H	-4.39650000	3.42990000	3.53040000
	C	-4.90200000	0.91600000	4.28240000
	H	-5.35640000	1.67570000	4.90620000
	C	-3.24270000	-2.08340000	1.74240000
	H	-2.48250000	-1.72780000	1.03590000
	C	-5.11590000	-0.43540000	4.55170000
	C	-3.53310000	0.28010000	2.39870000
	C	-3.76830000	-1.06440000	2.66750000
	N	-1.06780000	4.75730000	1.81290000
	C	-4.51080000	6.02010000	-0.37890000
	H	-5.28590000	6.08470000	-1.14840000
	H	-4.74850000	6.78240000	0.37340000
	C	-2.06500000	4.78770000	0.75430000
	H	-1.85900000	4.02020000	-0.00240000
	C	-3.45050000	4.44730000	1.33250000
	H	-3.64990000	5.11190000	2.18760000
	C	-4.54130000	4.63190000	0.26130000
	H	-5.51390000	4.43250000	0.72020000
	H	-4.39030000	3.86410000	-0.50250000
	C	-2.04280000	6.16850000	0.08690000
	H	-1.05730000	6.32440000	-0.35630000
	H	-2.16970000	6.93060000	0.86350000
	C	-3.13260000	6.30770000	-0.97750000
	H	-3.10120000	7.31150000	-1.41040000
	H	-2.93800000	5.60640000	-1.79590000
	N	-0.98620000	-4.45450000	1.75200000
	C	0.81970000	-4.09730000	3.28100000
	C	3.36990000	-3.12510000	3.93200000
	H	4.36420000	-2.76120000	4.14970000
	C	-0.46580000	-4.69950000	2.88100000
	H	-0.91460000	-5.40670000	3.59400000
	C	1.47730000	-4.56210000	4.42150000
	H	1.01820000	-5.31490000	5.05000000
	C	3.36160000	-1.70760000	1.87160000
	H	2.71000000	-1.26590000	1.10750000
	C	2.74330000	-4.06570000	4.73020000
	C	1.42990000	-3.11990000	2.48640000
	C	2.69970000	-2.64360000	2.79700000
	N	-3.67690000	-3.27410000	1.71740000
	C	-2.92830000	-6.92900000	-0.36300000
	H	-2.55960000	-7.64950000	-1.09930000
	H	-3.49630000	-7.50090000	0.38120000
	C	-3.15330000	-4.16910000	0.69610000
	H	-2.56650000	-3.61520000	-0.04690000

	C	-2.17810000	-5.17360000	1.33650000
	H	-2.67990000	-5.66120000	2.18680000
	C	-1.74360000	-6.23470000	0.30870000
	H	-1.09760000	-6.95960000	0.81230000
	H	-1.12870000	-5.73500000	-0.44450000
	C	-4.32400000	-4.86730000	-0.00590000
	H	-4.93950000	-4.10800000	-0.49270000
	H	-4.94820000	-5.34630000	0.75650000
	C	-3.84680000	-5.90180000	-1.02710000
	H	-4.70880000	-6.39320000	-1.48670000
	H	-3.30490000	-5.39750000	-1.83480000
	H	-2.93730000	0.58350000	1.54690000
	H	0.90960000	-2.78270000	1.59850000
	H	1.94670000	2.23970000	1.62110000
	H	-1.96480000	2.22440000	-1.62030000
	H	2.93390000	0.60770000	-1.54770000
	H	-0.88570000	-2.79070000	-1.59960000
	Br	2.08880000	5.61450000	6.29290000
	Br	3.64290000	-4.72520000	6.28890000
	Br	-6.20880000	-0.91960000	6.04990000
	Br	-2.14010000	5.60190000	-6.28900000
	Br	-3.60430000	-4.75510000	-6.28950000
	Br	6.21800000	-0.86910000	-6.05030000
**3c** (m)	O	-2.17720000	1.11270000	3.63110000
	O	0.12370000	-2.44040000	3.63000000
	N	2.19770000	4.19680000	-0.57310000
	N	4.33930000	-1.90350000	-0.95980000
	N	2.53470000	-4.00130000	-0.56730000
	N	-0.52460000	4.70660000	-0.95670000
	C	-1.49150000	3.15340000	0.62370000
	N	-4.73610000	-0.19700000	-0.56430000
	C	-1.45010000	2.72830000	1.95620000
	H	-0.81330000	3.25720000	2.65180000
	C	3.26740000	0.73090000	-0.31150000
	H	3.54000000	0.55930000	-1.34530000
	C	3.48350000	-0.28470000	0.61940000
	N	-3.81780000	-2.80790000	-0.95560000
	C	-2.19460000	1.61830000	2.36100000
	O	2.06640000	1.33620000	3.62710000
	C	-0.66280000	4.30400000	0.23950000
	H	-0.12700000	4.78670000	1.06860000
	C	-1.63960000	-2.61850000	1.95590000
	H	-2.41580000	-2.33310000	2.65230000
	C	0.32790000	-3.30680000	0.10510000
	C	1.78640000	5.11550000	-1.61650000
	H	1.60570000	4.58610000	-2.56590000
	C	-1.98760000	-2.86650000	0.62340000

	C	4.76500000	-3.27530000	-1.17440000
	H	5.12380000	-3.72480000	-0.23440000
	C	-0.99980000	-3.18870000	-0.30660000
	H	-1.28480000	-3.34260000	-1.33990000
	C	-2.26630000	2.45970000	-0.30540000
	H	-2.25730000	2.78300000	-1.33890000
	C	-3.03340000	1.36990000	0.10720000
	C	-3.00350000	0.95580000	1.44200000
	H	-3.59050000	0.10300000	1.75250000
	C	-0.30560000	-2.70680000	2.35980000
	C	0.67220000	-3.07390000	1.43970000
	H	1.70460000	-3.15500000	1.74950000
	C	2.33940000	2.12540000	1.43580000
	H	1.89520000	3.06110000	1.74510000
	C	2.70840000	1.94110000	0.10030000
	C	3.53410000	-4.11000000	-1.61180000
	H	3.16450000	-3.69220000	-2.56210000
	C	-5.32850000	-1.00930000	-1.60880000
	H	-4.78170000	-0.89740000	-2.55900000
	C	4.06300000	-1.57930000	0.23640000
	H	4.21440000	-2.28290000	1.06670000
	C	2.51080000	1.09590000	2.35680000
	C	0.44900000	5.76200000	-1.17330000
	H	0.66150000	6.29710000	-0.23360000
	C	-3.81630000	0.61460000	-0.88530000
	H	-3.51980000	0.76780000	-1.93340000
	C	3.09920000	-0.10470000	1.95280000
	H	3.24130000	-0.91890000	2.64980000
	C	-3.39890000	-2.72580000	0.24030000
	H	-4.08390000	-2.50200000	1.06980000
	C	2.44230000	2.99470000	-0.89340000
	H	2.42490000	2.65970000	-1.94100000
	C	-5.21900000	-2.49250000	-1.17130000
	H	-5.78870000	-2.57920000	-0.23200000
	C	5.87060000	-3.35940000	-2.22750000
	H	6.72720000	-2.76640000	-1.89480000
	H	5.50740000	-2.89750000	-3.15220000
	C	3.94770000	-5.57010000	-1.83390000
	H	3.07220000	-6.14330000	-2.15230000
	H	4.27020000	-5.99020000	-0.87490000
	C	1.37310000	-3.60720000	-0.88770000
	H	1.09260000	-3.42460000	-1.93550000
	C	-5.84230000	-3.40870000	-2.22490000
	H	-5.75520000	-4.44700000	-1.89260000
	H	-5.26060000	-3.32350000	-3.14930000
	C	-1.34370000	1.76210000	4.58670000
	H	-0.29750000	1.76180000	4.26720000

	H	-1.44940000	1.19140000	5.50800000
	H	-1.67520000	2.79060000	4.76420000
	C	2.84240000	6.20460000	-1.84340000
	H	3.77530000	5.73390000	-2.16690000
	H	3.04950000	6.69320000	-0.88510000
	C	6.28000000	-4.81420000	-2.48180000
	H	7.04430000	-4.85850000	-3.26320000
	H	6.73740000	-5.22510000	-1.57320000
	C	2.21490000	0.29240000	4.58530000
	H	1.77770000	0.67270000	5.50710000
	H	3.27190000	0.06450000	4.75910000
	H	1.68910000	-0.61380000	4.27080000
	C	5.07230000	-5.67570000	-2.87030000
	H	5.37250000	-6.72110000	-2.98840000
	H	4.69480000	-5.34620000	-3.84600000
	C	-6.80030000	-0.63950000	-1.83140000
	H	-7.32560000	-0.70980000	-0.87270000
	H	-6.86060000	0.40530000	-2.14970000
	C	-0.03590000	6.76190000	-2.22370000
	H	-0.97710000	7.20580000	-1.88720000
	H	-0.25700000	6.21700000	-3.14800000
	C	-0.85640000	-2.04940000	4.58720000
	H	-1.38450000	-1.14570000	4.26940000
	H	-0.30920000	-1.85390000	5.50820000
	H	-1.57700000	-2.85460000	4.76480000
	C	2.36590000	7.23220000	-2.87650000
	H	3.11980000	8.01570000	-2.99700000
	H	2.26510000	6.74180000	-3.85240000
	C	1.01720000	7.84520000	-2.48060000
	H	0.66900000	8.53020000	-3.25920000
	H	1.14770000	8.44500000	-1.57150000
	C	-7.45230000	-1.56180000	-2.86790000
	H	-8.50810000	-1.30110000	-2.98600000
	H	-6.97840000	-1.39860000	-3.84360000
	C	-7.30750000	-3.03830000	-2.47930000
	H	-7.72640000	-3.67890000	-3.26070000
	H	-7.89170000	-3.23010000	-1.57090000
**3c** (t-to-t)	O	-2.99310000	-4.77040000	2.35830000
	O	2.50050000	0.81480000	0.65090000
	N	-7.52110000	0.95320000	0.38410000
	N	-2.67220000	4.82050000	-1.35810000
	N	-0.17590000	3.73880000	-2.38330000
	N	-7.49590000	-1.80210000	-0.40460000
	C	-5.55280000	-2.98860000	0.37410000
	N	-2.08060000	-4.47320000	-2.62930000
	C	-4.89960000	-3.52210000	1.48970000
	H	-5.34850000	-3.40210000	2.46640000

	C	-4.64240000	3.13110000	-0.10380000
	H	-4.75900000	3.33590000	-1.16040000
	C	-3.58160000	3.72270000	0.58110000
	N	-0.12060000	-2.59950000	-3.62200000
	C	-3.68680000	-4.20060000	1.33150000
	O	-4.10260000	2.21620000	3.91350000
	C	-6.82990000	-2.28100000	0.56240000
	H	-7.17350000	-2.18620000	1.60270000
	C	1.58990000	-0.77370000	-0.96600000
	H	2.09950000	-1.62530000	-0.53510000
	C	0.23370000	1.38920000	-2.10750000
	C	-8.56120000	0.38890000	-0.45430000
	H	-8.29470000	0.45410000	-1.52200000
	C	0.74490000	-0.97000000	-2.06890000
	C	-1.63950000	5.64630000	-1.95210000
	H	-0.89330000	5.95880000	-1.20270000
	C	0.06980000	0.11070000	-2.64020000
	H	-0.57580000	-0.06810000	-3.49100000
	C	-4.98380000	-3.12170000	-0.89260000
	H	-5.50450000	-2.69850000	-1.74260000
	C	-3.76070000	-3.77380000	-1.04530000
	C	-3.11230000	-4.31620000	0.06780000
	H	-2.16720000	-4.82820000	-0.05580000
	C	1.74190000	0.50680000	-0.43160000
	C	1.05620000	1.57590000	-0.99870000
	H	1.14640000	2.55320000	-0.55730000
	C	-5.30920000	1.99290000	1.92340000
	H	-5.97530000	1.31390000	2.43830000
	C	-5.50900000	2.27060000	0.56770000
	C	-0.89510000	4.82170000	-3.02560000
	H	-1.65340000	4.43960000	-3.72800000
	C	-1.61320000	-4.50030000	-4.00240000
	H	-2.22680000	-3.84440000	-4.64160000
	C	-2.60400000	4.57420000	-0.11410000
	H	-1.78420000	4.95890000	0.50840000
	C	-4.24280000	2.57130000	2.60520000
	C	-8.73050000	-1.10320000	-0.10260000
	H	-8.96100000	-1.15380000	0.97400000
	C	-3.16690000	-3.86590000	-2.38890000
	H	-3.73470000	-3.36240000	-3.18550000
	C	-3.38190000	3.44760000	1.93730000
	H	-2.51680000	3.87050000	2.43000000
	C	0.59380000	-2.33070000	-2.60460000
	H	1.14760000	-3.11390000	-2.07460000
	C	-6.61320000	1.64340000	-0.17250000
	H	-6.60330000	1.81080000	-1.26050000
	C	-0.16320000	-3.98320000	-4.06160000

	H	0.44240000	-4.62740000	-3.40620000
	C	-2.28960000	6.89030000	-2.57480000
	H	-2.77640000	7.46410000	-1.78110000
	H	-3.08300000	6.55600000	-3.25210000
	C	0.10950000	5.70010000	-3.78700000
	H	0.59480000	5.09250000	-4.55670000
	H	0.89490000	6.01030000	-3.08830000
	C	-0.46330000	2.54070000	-2.69620000
	H	-1.23920000	2.30830000	-3.43870000
	C	0.36560000	-4.07390000	-5.50030000
	H	1.39200000	-3.69860000	-5.52450000
	H	-0.22550000	-3.39850000	-6.12820000
	C	-3.53890000	-4.68180000	3.66760000
	H	-3.60400000	-3.64130000	4.00250000
	H	-2.85190000	-5.22490000	4.31180000
	H	-4.52840000	-5.14750000	3.71810000
	C	-9.87830000	1.14440000	-0.21970000
	H	-9.73550000	2.19200000	-0.49990000
	H	-10.09610000	1.13230000	0.85390000
	C	-1.27930000	7.75400000	-3.33390000
	H	-1.78740000	8.61030000	-3.78710000
	H	-0.54260000	8.16250000	-2.63090000
	C	-2.94900000	2.67810000	4.60520000
	H	-3.00930000	2.24580000	5.60250000
	H	-2.93980000	3.77090000	4.68440000
	H	-2.03230000	2.34180000	4.11380000
	C	-0.55390000	6.93290000	-4.40420000
	H	0.19640000	7.54390000	-4.91480000
	H	-1.27520000	6.61630000	-5.16750000
	C	-1.69230000	-5.93890000	-4.53550000
	H	-1.12430000	-6.58850000	-3.86070000
	H	-2.73270000	-6.27370000	-4.49420000
	C	-9.88550000	-1.72340000	-0.89880000
	H	-9.98850000	-2.77430000	-0.61340000
	H	-9.61920000	-1.71010000	-1.96150000
	C	3.26400000	-0.22210000	1.24790000
	H	2.61580000	-1.00730000	1.64940000
	H	3.81630000	0.24570000	2.06100000
	H	3.96980000	-0.65340000	0.53440000
	C	-11.03690000	0.52250000	-1.00450000
	H	-11.96370000	1.06350000	-0.79190000
	H	-10.84840000	0.63290000	-2.07950000
	C	-11.19630000	-0.96460000	-0.67180000
	H	-11.99370000	-1.40820000	-1.27520000
	H	-11.50080000	-1.06900000	0.37680000
	C	-1.14390000	-6.04400000	-5.96110000
	H	-1.18070000	-7.08380000	-6.29910000

	H	-1.78480000	-5.46930000	-6.64070000
	C	0.28720000	-5.50320000	-6.04410000
	H	0.64850000	-5.53310000	-7.07630000
	H	0.94850000	-6.15570000	-5.45980000
	O	4.10140000	2.21970000	-3.91430000
	N	2.08200000	-4.47370000	2.63000000
	N	0.17540000	3.73870000	2.38450000
	N	0.12230000	-2.59970000	3.62320000
	O	-2.49970000	0.81380000	-0.64980000
	C	-1.05560000	1.57540000	0.99980000
	H	-1.14620000	2.55260000	0.55830000
	N	2.67090000	4.82130000	1.35870000
	C	-0.74340000	-0.97040000	2.06990000
	O	2.99350000	-4.77360000	-2.35770000
	C	-0.23310000	1.38900000	2.10860000
	N	7.52010000	0.95510000	-0.38570000
	C	-0.06870000	0.11050000	2.64120000
	H	0.57690000	-0.06800000	3.49210000
	C	3.58050000	3.72470000	-0.58110000
	C	4.24160000	2.57420000	-2.60580000
	C	5.30820000	1.99560000	-1.92440000
	H	5.97430000	1.31690000	-2.43960000
	C	3.38070000	3.45010000	-1.93740000
	H	2.51550000	3.87310000	-2.42980000
	N	7.49490000	-1.80060000	0.40250000
	C	1.61510000	-4.50040000	4.00330000
	H	2.22880000	-3.84430000	4.64210000
	C	0.89420000	4.82190000	3.02660000
	H	1.65270000	4.44020000	3.72900000
	C	0.16510000	-3.98330000	4.06290000
	H	-0.44070000	-4.62760000	3.40780000
	C	3.16770000	-3.86560000	2.38880000
	H	3.73540000	-3.36120000	3.18500000
	C	3.68700000	-4.20240000	-1.33140000
	C	2.60290000	4.57570000	0.11450000
	H	1.78310000	4.96070000	-0.50780000
	C	-1.58840000	-0.77440000	0.96700000
	H	-2.09760000	-1.62620000	0.53610000
	C	-1.74100000	0.50600000	0.43260000
	C	-0.59200000	-2.33110000	2.60570000
	H	-1.14560000	-3.11440000	2.07570000
	C	5.50810000	2.27270000	-0.56860000
	C	4.64150000	3.13290000	0.10350000
	H	4.75820000	3.33720000	1.16010000
	C	0.46350000	2.54080000	2.69720000
	H	1.23970000	2.30860000	3.43950000
	C	-3.26300000	-0.22350000	-1.24670000

	H	-2.61450000	-1.00840000	-1.64840000
	H	-3.81560000	0.24420000	-2.05960000
	H	-3.96840000	-0.65510000	-0.53310000
	C	1.63810000	5.64670000	1.95300000
	H	0.89170000	5.95910000	1.20380000
	C	4.89920000	-3.52310000	-1.49040000
	H	5.34780000	-3.40350000	-2.46720000
	C	3.76090000	-3.77390000	1.04500000
	C	5.55220000	-2.98830000	-0.37520000
	C	-0.11060000	5.70000000	3.78810000
	H	-0.89610000	6.01000000	3.08940000
	H	-0.59570000	5.09230000	4.55780000
	C	4.98360000	-3.12090000	0.89160000
	H	5.50420000	-2.69690000	1.74130000
	C	6.61240000	1.64520000	0.17130000
	H	6.60280000	1.81230000	1.25930000
	C	-0.28470000	-5.50300000	6.04580000
	H	-0.64560000	-5.53270000	7.07820000
	H	-0.94600000	-6.15580000	5.46190000
	C	-0.36330000	-4.07390000	5.50180000
	H	0.22790000	-3.39820000	6.12940000
	H	-1.38980000	-3.69860000	5.52620000
	C	8.72930000	-1.10160000	0.10010000
	H	8.95940000	-1.15200000	-0.97670000
	C	3.11270000	-4.31750000	-0.06760000
	H	2.16800000	-4.83010000	0.05660000
	C	6.82890000	-2.28020000	-0.56420000
	H	7.17240000	-2.18580000	-1.60460000
	C	3.53990000	-4.68710000	-3.66690000
	H	3.60420000	-3.64720000	-4.00380000
	H	2.85390000	-5.23230000	-4.31040000
	H	4.53000000	-5.15190000	-3.71590000
	C	1.69450000	-5.93880000	4.53680000
	H	1.12640000	-6.58860000	3.86240000
	H	2.73490000	-6.27350000	4.49530000
	C	8.56030000	0.39040000	0.45220000
	H	8.29410000	0.45530000	1.52000000
	C	2.28800000	6.89100000	2.57560000
	H	3.08160000	6.55680000	3.25280000
	H	2.77450000	7.46490000	1.78190000
	C	11.03620000	0.52370000	1.00160000
	H	11.96300000	1.06470000	0.78880000
	H	10.84810000	0.63380000	2.07670000
	C	2.94760000	2.68140000	-4.60560000
	H	2.03100000	2.34480000	-4.11410000
	H	3.00770000	2.24940000	-5.60300000
	H	2.93810000	3.77430000	-4.68460000

	C	11.19540000	-0.96330000	0.66840000
	H	11.99300000	-1.40710000	1.27130000
	H	11.49950000	-1.06750000	-0.38040000
	C	0.55250000	6.93310000	4.40520000
	H	-0.19790000	7.54380000	4.91590000
	H	1.27390000	6.61660000	5.16850000
	C	1.27750000	7.75430000	3.33490000
	H	1.78550000	8.61070000	3.78810000
	H	0.54070000	8.16270000	2.63200000
	C	1.14650000	-6.04360000	5.96260000
	H	1.18360000	-7.08330000	6.30080000
	H	1.78750000	-5.46860000	6.64180000
	C	9.88460000	-1.72210000	0.89570000
	H	9.61870000	-1.70910000	1.95840000
	H	9.98740000	-2.77290000	0.60990000
	C	9.87740000	1.14590000	0.21740000
	H	10.09480000	1.13400000	-0.85630000
	H	9.73480000	2.19340000	0.49790000
**3c** (h-to-h)	N	-3.40400000	-3.05800000	-1.76350000
	C	-4.00290000	-1.28790000	-3.26240000
	C	-4.43600000	1.40150000	-3.88520000
	H	-4.62800000	2.44240000	-4.10840000
	C	-3.87790000	-2.70750000	-2.88500000
	H	-4.26460000	-3.44000000	-3.61030000
	C	-4.73520000	-0.94180000	-4.40340000
	H	-5.14380000	-1.72930000	-5.02280000
	C	-3.20280000	2.08300000	-1.82020000
	H	-2.48400000	1.72670000	-1.07240000
	C	-4.94620000	0.40590000	-4.71630000
	C	-3.46040000	-0.28690000	-2.45590000
	C	-3.68880000	1.05620000	-2.75820000
	N	-1.02710000	-4.78230000	-1.82770000
	C	-4.50580000	-6.02710000	0.31840000
	H	-5.29230000	-6.08840000	1.07710000
	H	-4.73700000	-6.78740000	-0.43840000
	C	-2.03930000	-4.80730000	-0.78220000
	H	-1.83980000	-4.04220000	-0.02190000
	C	-3.41550000	-4.45700000	-1.37750000
	H	-3.60750000	-5.11900000	-2.23710000
	C	-4.52020000	-4.63810000	-0.32050000
	H	-5.48640000	-4.43230000	-0.79030000
	H	-4.37330000	-3.87280000	0.44630000
	C	-2.03330000	-6.18750000	-0.11430000
	H	-1.05450000	-6.34630000	0.34230000
	H	-2.15300000	-6.94960000	-0.89260000
	C	-3.13740000	-6.32200000	0.93610000
	H	-3.11650000	-7.32650000	1.36880000

	H	-2.94910000	-5.62080000	1.75610000
	N	-0.93990000	4.47770000	-1.76680000
	C	0.88920000	4.10550000	-3.26810000
	C	3.43410000	3.13370000	-3.89010000
	H	4.43150000	2.77900000	-4.11240000
	C	-0.40170000	4.70870000	-2.89040000
	H	-0.84600000	5.40400000	-3.61930000
	C	1.55490000	4.56440000	-4.41030000
	H	1.07750000	5.31130000	-5.03080000
	C	3.40890000	1.73010000	-1.82240000
	H	2.74270000	1.29100000	-1.06930000
	C	2.82760000	4.07280000	-4.72230000
	C	1.48510000	3.13700000	-2.45960000
	C	2.76160000	2.66160000	-2.76220000
	N	-3.62160000	3.28000000	-1.82480000
	C	-2.96430000	6.92560000	0.30200000
	H	-2.62550000	7.64410000	1.05500000
	H	-3.50990000	7.49850000	-0.45850000
	C	-3.13620000	4.17210000	-0.78200000
	H	-2.57260000	3.61980000	-0.02010000
	C	-2.14660000	5.18700000	-1.38300000
	H	-2.62480000	5.67870000	-2.24530000
	C	-1.75270000	6.24230000	-0.33270000
	H	-1.09330000	6.97470000	-0.80760000
	H	-1.16210000	5.73920000	0.43750000
	C	-4.33450000	4.85910000	-0.11700000
	H	-4.96060000	4.09230000	0.34330000
	H	-4.93450000	5.33950000	-0.89800000
	C	-3.89970000	5.88910000	0.92750000
	H	-4.78060000	6.37320000	1.35930000
	H	-3.38370000	5.38140000	1.74970000
	N	4.35250000	-1.42910000	-1.76080000
	C	3.11890000	-2.82680000	-3.26480000
	C	1.00500000	-4.54340000	-3.89180000
	H	0.19910000	-5.22890000	-4.11680000
	C	4.28560000	-2.00990000	-2.88490000
	H	5.11200000	-1.97310000	-3.61150000
	C	3.18420000	-3.63010000	-4.40870000
	H	4.07000000	-3.58820000	-5.02880000
	C	-0.19850000	-3.82210000	-1.82240000
	H	-0.24750000	-3.02250000	-1.07320000
	C	2.12190000	-4.48530000	-4.72350000
	C	1.98200000	-2.86040000	-2.45710000
	C	0.93220000	-3.72770000	-2.76180000
	N	4.65430000	1.49010000	-1.83160000
	C	7.47500000	-0.89280000	0.32020000
	H	7.92130000	-1.54000000	1.08170000

	H	8.24800000	-0.71830000	-0.43880000
	C	5.18430000	0.63040000	-0.78390000
	H	4.42360000	0.42410000	-0.02130000
	C	5.56900000	-0.73870000	-1.37440000
	H	6.23960000	-0.57690000	-2.23360000
	C	6.27670000	-1.60060000	-0.31260000
	H	6.57840000	-2.54410000	-0.77680000
	H	5.54030000	-1.84890000	0.45660000
	C	6.37910000	1.32810000	-0.12300000
	H	6.02990000	2.25830000	0.32970000
	H	7.09700000	1.59980000	-0.90490000
	C	7.04990000	0.44420000	0.93060000
	H	7.91130000	0.96590000	1.35800000
	H	6.35090000	0.26290000	1.75390000
	N	-4.36530000	-1.40000000	1.75830000
	C	-3.13830000	-2.80490000	3.26100000
	C	-1.03200000	-4.53030000	3.88840000
	H	-0.22890000	-5.21900000	4.11380000
	C	-4.30130000	-1.98210000	2.88170000
	H	-5.12670000	-1.94120000	3.60920000
	C	-3.20760000	-3.60840000	4.40450000
	H	-4.09350000	-3.56310000	5.02410000
	C	0.17750000	-3.81240000	1.82160000
	H	0.23190000	-3.01210000	1.07340000
	C	-2.14910000	-4.46820000	4.71940000
	C	-2.00070000	-2.84210000	2.45430000
	C	-0.95440000	-3.71370000	2.75930000
	N	-4.64550000	1.52120000	1.82140000
	C	-7.48790000	-0.84360000	-0.31900000
	H	-7.94260000	-1.49050000	-1.07580000
	H	-8.25800000	-0.65500000	0.43950000
	C	-5.18060000	0.66140000	0.77610000
	H	-4.42010000	0.44470000	0.01600000
	C	-5.57720000	-0.70130000	1.37260000
	H	-6.24360000	-0.53000000	2.23310000
	C	-6.29700000	-1.56140000	0.31710000
	H	-6.60910000	-2.49820000	0.78770000
	H	-5.56530000	-1.82350000	-0.45190000
	C	-6.36770000	1.36700000	0.10960000
	H	-6.00750000	2.29020000	-0.34870000
	H	-7.08310000	1.65140000	0.88930000
	C	-7.04700000	0.48380000	-0.93880000
	H	-7.90180000	1.01250000	-1.37090000
	H	-6.34900000	0.28860000	-1.75990000
	N	3.39400000	-3.07160000	1.76780000
	C	3.99990000	-1.30500000	3.26690000
	C	4.44780000	1.38280000	3.88590000

	H	4.64600000	2.42290000	4.10720000
	C	3.86580000	-2.72400000	2.89100000
	H	4.24080000	-3.45880000	3.62020000
	C	4.73250000	-0.96150000	4.40860000
	H	5.13570000	-1.75020000	5.03000000
	C	3.22070000	2.06820000	1.81850000
	H	2.49970000	1.71520000	1.07110000
	C	4.95140000	0.38560000	4.71920000
	C	3.46490000	-0.30230000	2.45790000
	C	3.70000000	1.04000000	2.75850000
	N	1.00100000	-4.77670000	1.82890000
	C	4.47520000	-6.05520000	-0.30490000
	H	5.26350000	-6.12520000	-1.06100000
	H	4.69700000	-6.81630000	0.45390000
	C	2.01540000	-4.81020000	0.78570000
	H	1.82330000	-4.04500000	0.02350000
	C	3.39340000	-4.47120000	1.38400000
	H	3.57630000	-5.13320000	2.24550000
	C	4.50010000	-4.66480000	0.33100000
	H	5.46670000	-4.46670000	0.80330000
	H	4.36310000	-3.90010000	-0.43830000
	C	2.00030000	-6.19200000	0.12140000
	H	1.02110000	-6.34500000	-0.33660000
	H	2.11260000	-6.95240000	0.90220000
	C	3.10600000	-6.33810000	-0.92580000
	H	3.07700000	-7.34280000	-1.35780000
	H	2.92690000	-5.63620000	-1.74710000
	N	0.96530000	4.47270000	1.76840000
	C	-0.86620000	4.10920000	3.26850000
	C	-3.41670000	3.15020000	3.88710000
	H	-4.41590000	2.80030000	4.10870000
	C	0.42900000	4.70460000	2.89270000
	H	0.87740000	5.39640000	3.62260000
	C	-1.52940000	4.56880000	4.41190000
	H	-1.04810000	5.31110000	5.03480000
	C	-3.39820000	1.75050000	1.81640000
	H	-2.73210000	1.30340000	1.06830000
	C	-2.80490000	4.08340000	4.72210000
	C	-1.46700000	3.14580000	2.45760000
	C	-2.74660000	2.67710000	2.75820000
	N	3.64280000	3.26430000	1.82440000
	C	2.99470000	6.91000000	-0.30600000
	H	2.65660000	7.62890000	-1.05900000
	H	3.54560000	7.48170000	0.45150000
	C	3.15870000	4.15660000	0.78130000
	H	2.59040000	3.60450000	0.02270000
	C	2.17460000	5.17650000	1.38330000

	H	2.65670000	5.66620000	2.24460000
	C	1.78250000	6.23290000	0.33380000
	H	1.12800000	6.96850000	0.81070000
	H	1.18650000	5.73210000	-0.43370000
	C	4.35800000	4.83790000	0.11070000
	H	4.97980000	4.06890000	-0.35210000
	H	4.96380000	5.31630000	0.88840000
	C	3.92330000	5.86850000	-0.93320000
	H	4.80450000	6.34830000	-1.36920000
	H	3.40170000	5.36180000	-1.75230000
	H	2.91050000	-0.59090000	1.57420000
	H	-0.94070000	2.81240000	1.57230000
	H	-1.97300000	-2.22030000	1.56860000
	H	1.95770000	-2.23930000	-1.57090000
	H	-2.90520000	-0.57680000	-1.57300000
	H	0.95790000	2.80470000	-1.57450000
	O	2.09700000	-5.30560000	-5.81170000
	O	-5.64700000	0.83820000	-5.80260000
	O	3.55400000	4.46250000	-5.80780000
	C	2.99250000	5.43100000	-6.67970000
	H	3.73090000	5.58890000	-7.46360000
	H	2.05870000	5.07420000	-7.12850000
	H	2.80700000	6.37850000	-6.16140000
	C	-6.20100000	-0.13480000	-6.67440000
	H	-6.70650000	0.42350000	-7.46010000
	H	-5.42260000	-0.76390000	-7.12040000
	H	-6.92850000	-0.77010000	-6.15660000
	C	3.21760000	-5.29970000	-6.68240000
	H	2.98630000	-6.01510000	-7.46930000
	H	3.37580000	-4.31080000	-7.12710000
	H	4.13040000	-5.61450000	-6.16420000
	O	5.65420000	0.81560000	5.80490000
	O	-3.52880000	4.47340000	5.80920000
	O	-2.12740000	-5.28850000	5.80760000
	C	-2.96180000	5.43650000	6.68350000
	H	-3.69930000	5.59680000	7.46760000
	H	-2.03010000	5.07330000	7.13150000
	H	-2.77080000	6.38420000	6.16730000
	C	-3.24680000	-5.27720000	6.67970000
	H	-3.01750000	-5.99310000	7.46680000
	H	-3.40010000	-4.28730000	7.12400000
	H	-4.16180000	-5.58850000	6.16320000
	C	6.20310000	-0.15910000	6.67800000
	H	6.71080000	0.39750000	7.46350000
	H	5.42150000	-0.78420000	7.12420000
	H	6.92780000	-0.79840000	6.16130000
**3d** (m)	N	-4.76140000	0.63280000	-0.79900000

	O	-4.16970000	2.11840000	3.91030000
	N	2.93280000	3.79410000	-0.81340000
	N	-3.24260000	2.86030000	3.62160000
	O	0.26770000	-4.61320000	3.92620000
	O	-2.65930000	3.59450000	4.40660000
	C	-3.69940000	1.27380000	-1.05520000
	H	-3.22360000	1.24570000	-2.04600000
	N	-4.18230000	-2.15150000	-1.03920000
	C	3.90080000	0.97740000	2.20160000
	N	4.11330000	1.38150000	3.61090000
	N	3.94960000	-2.54970000	-1.04910000
	C	-1.93940000	2.89980000	-0.40540000
	H	-1.58740000	2.92780000	-1.42990000
	C	-3.02090000	2.10160000	-0.04280000
	N	0.22860000	4.67750000	-1.05200000
	C	-5.35460000	-0.17580000	-1.84590000
	H	-4.71140000	-0.20890000	-2.73990000
	N	1.82060000	-4.43320000	-0.79340000
	C	3.35010000	1.55640000	-0.05500000
	C	-1.55800000	-3.13530000	-0.39670000
	H	-1.76460000	-2.85600000	-1.42320000
	C	-6.72670000	0.39780000	-2.22790000
	H	-7.32940000	0.48580000	-1.31760000
	H	-6.58950000	1.41150000	-2.61500000
	C	3.55540000	1.94350000	1.27670000
	H	3.44250000	2.97740000	1.56730000
	C	4.05990000	-0.36280000	1.86030000
	H	4.33230000	-1.07770000	2.62470000
	O	3.93330000	2.55610000	3.89510000
	C	-3.45660000	2.09020000	1.28970000
	H	-4.29660000	1.47830000	1.58330000
	C	-1.28130000	3.69920000	0.53950000
	C	-0.32160000	-3.66290000	-0.03500000
	C	-2.57470000	-2.95850000	0.55190000
	C	2.96900000	2.55420000	-1.06980000
	H	2.71280000	2.15350000	-2.06110000
	C	-0.08460000	-4.01770000	1.30040000
	H	0.86940000	-4.43000000	1.59370000
	C	-5.50260000	-1.61940000	-1.32070000
	H	-6.11430000	-1.56910000	-0.40600000
	C	3.50360000	0.22000000	-0.41330000
	H	3.35370000	-0.10210000	-1.43710000
	C	-3.89120000	-2.42410000	0.16410000
	H	-4.60710000	-2.27950000	0.98490000
	C	-2.78940000	2.87350000	2.21120000
	C	-1.70820000	3.67930000	1.86550000
	H	-1.22400000	4.27500000	2.62730000

	C	-2.33520000	-3.30210000	1.88060000
	H	-3.08920000	-3.17440000	2.64540000
	C	4.05120000	-2.15640000	0.15170000
	H	4.28790000	-2.84620000	0.97340000
	C	3.86550000	-0.74660000	0.53520000
	O	4.45650000	0.51230000	4.39960000
	C	-0.15680000	4.56710000	0.15060000
	H	0.32420000	5.11870000	0.97000000
	C	-1.09230000	-3.82560000	2.22540000
	C	1.34820000	5.55680000	-1.33290000
	H	1.69740000	6.06000000	-0.41730000
	N	-0.84320000	-4.19240000	3.63910000
	C	-7.57230000	-1.92700000	-2.74930000
	H	-8.23890000	-1.94510000	-1.87870000
	H	-8.03670000	-2.55820000	-3.51220000
	C	2.81290000	-4.55020000	-1.84400000
	H	2.51880000	-3.97780000	-2.73840000
	C	3.70590000	5.61340000	-2.24490000
	H	4.08610000	6.08940000	-1.33480000
	H	4.51440000	4.98920000	-2.63600000
	C	2.52530000	4.71010000	-1.86080000
	H	2.17470000	4.16820000	-2.75380000
	C	-6.20780000	-2.50220000	-2.35790000
	H	-5.56270000	-2.58270000	-3.23960000
	H	-6.31230000	-3.51150000	-1.95010000
	C	4.14170000	-3.96120000	-1.32550000
	H	4.40450000	-4.51360000	-0.40950000
	C	0.93320000	6.61140000	-2.36660000
	H	0.54060000	6.09500000	-3.24940000
	H	0.11110000	7.20400000	-1.95590000
	C	2.99380000	-6.02720000	-2.22330000
	H	2.04440000	-6.41090000	-2.60740000
	H	3.21810000	-6.59280000	-1.31280000
	C	0.73050000	-3.84170000	-1.05070000
	H	0.51040000	-3.42610000	-2.04450000
	C	-7.44030000	-0.48650000	-3.25470000
	H	-6.87440000	-0.48340000	-4.19430000
	H	-8.42550000	-0.06920000	-3.48130000
	C	4.11290000	-6.21100000	-3.25270000
	H	4.23780000	-7.27390000	-3.47780000
	H	3.82760000	-5.72230000	-4.19220000
	C	3.29320000	6.67630000	-3.26760000
	H	3.01070000	6.18720000	-4.20780000
	H	4.14600000	7.32220000	-3.49460000
	C	2.11180000	7.50790000	-2.75670000
	H	2.43040000	8.09140000	-1.88450000
	H	1.79530000	8.22800000	-3.51640000

	C	5.25540000	-4.13970000	-2.36500000
	H	5.00390000	-3.54230000	-3.24810000
	H	6.18480000	-3.72920000	-1.96060000
	O	-1.76710000	-4.05110000	4.42810000
	C	5.43100000	-5.61120000	-2.75190000
	H	6.20770000	-5.70500000	-3.51610000
	H	5.77810000	-6.17870000	-1.88010000
**3d** (t-to-t)	O	2.51150000	1.57190000	1.72230000
	N	0.18150000	3.70140000	-2.20330000
	N	-1.80970000	-4.32080000	-2.88770000
	N	-2.38080000	4.88640000	-1.51720000
	C	0.56920000	0.09040000	-2.54740000
	H	0.08080000	-0.03600000	-3.50620000
	N	-7.38680000	1.14320000	0.02710000
	O	-5.05800000	1.94750000	4.36220000
	N	0.38680000	-2.58160000	-3.63120000
	C	0.69760000	1.35380000	-1.97310000
	N	2.37340000	0.45050000	1.25120000
	C	-5.39780000	2.45710000	0.28210000
	C	1.02530000	-1.05860000	-1.88640000
	C	1.77390000	0.33470000	-0.08770000
	C	0.26560000	-3.95270000	-4.10060000
	H	0.70320000	-4.66070000	-3.38580000
	C	1.63570000	-0.93340000	-0.64180000
	H	1.98960000	-1.79750000	-0.09820000
	C	-1.28580000	5.66150000	-2.06770000
	H	-0.62340000	6.03670000	-1.27000000
	C	0.84710000	-2.39980000	-2.46510000
	H	1.13200000	-3.23370000	-1.81370000
	C	1.30860000	1.47620000	-0.71650000
	H	1.36590000	2.43340000	-0.22630000
	C	-4.47320000	3.29150000	-0.34200000
	H	-4.48540000	3.43050000	-1.41550000
	C	-5.31870000	-2.88460000	0.09440000
	O	-3.22860000	3.11090000	4.40290000
	C	-4.33150000	2.89510000	2.37880000
	C	-5.32570000	2.25090000	1.66670000
	H	-6.02840000	1.59280000	2.15600000
	N	-7.20480000	-1.62870000	-0.69220000
	C	-0.43330000	4.76450000	-2.98810000
	H	-1.09880000	4.34710000	-3.75870000
	C	-6.43530000	1.78090000	-0.51500000
	H	-6.33170000	1.86670000	-1.60570000
	C	-3.50410000	-3.63000000	-1.34560000
	N	-4.19990000	2.63250000	3.82650000
	O	-2.08870000	-5.83880000	1.82710000
	C	-2.83960000	-3.61990000	-2.66270000

	H	-3.29700000	-2.97610000	-3.42460000
	C	-3.48160000	3.94690000	0.39830000
	C	-2.44270000	4.75890000	-0.25700000
	H	-1.69410000	5.19720000	0.41290000
	C	0.11570000	2.52040000	-2.66120000
	H	-0.40220000	2.29170000	-3.60050000
	C	-2.97820000	-4.38710000	-0.29030000
	H	-2.08700000	-4.97930000	-0.44060000
	C	-6.58900000	-2.16110000	0.27900000
	H	-6.98180000	-2.13560000	1.30510000
	C	-4.66740000	-2.89170000	-1.14620000
	H	-5.09240000	-2.30460000	-1.95030000
	C	-3.41730000	3.75070000	1.77550000
	H	-2.63670000	4.20390000	2.36420000
	C	-1.22790000	-4.31640000	-4.21750000
	H	-1.71620000	-3.57570000	-4.87020000
	C	-4.77580000	-3.60600000	1.15550000
	H	-5.23720000	-3.61180000	2.13350000
	C	-8.47470000	-0.97850000	-0.42940000
	H	-8.72540000	-1.01350000	0.64280000
	N	-3.05160000	-5.11880000	2.05980000
	C	-1.86110000	6.85180000	-2.84940000
	H	-2.57220000	6.46490000	-3.58710000
	H	-2.43390000	7.47850000	-2.15990000
	C	-8.37170000	0.50730000	-0.82570000
	H	-8.07470000	0.55290000	-1.88560000
	C	0.98200000	-4.09060000	-5.44890000
	H	2.03770000	-3.85100000	-5.30320000
	H	0.57230000	-3.34800000	-6.14270000
	C	-3.61890000	-4.34870000	0.93620000
	C	0.82200000	-5.50140000	-6.01860000
	H	1.32480000	-5.57130000	-6.98740000
	H	1.31810000	-6.21470000	-5.35070000
	C	0.66670000	5.59390000	-3.67230000
	H	1.34910000	5.97330000	-2.90270000
	H	1.25340000	4.93890000	-4.32050000
	C	-9.72860000	1.20480000	-0.64670000
	H	-9.97300000	1.21370000	0.42090000
	H	-9.63020000	2.24900000	-0.95650000
	C	-1.38590000	-5.72420000	-4.81560000
	H	-0.98650000	-6.44350000	-4.09340000
	H	-2.45280000	-5.94090000	-4.92360000
	C	-9.57890000	-1.67930000	-1.23340000
	H	-9.63740000	-2.72410000	-0.91570000
	H	-9.28680000	-1.68590000	-2.28910000
	C	-0.65890000	-5.86800000	-6.15530000
	H	-0.76970000	-6.89190000	-6.52420000

	H	-1.12750000	-5.21240000	-6.89980000
	C	0.08220000	6.77180000	-4.45560000
	H	0.89020000	7.34790000	-4.91520000
	H	-0.53930000	6.39130000	-5.27490000
	C	-10.92970000	-0.97950000	-1.06100000
	H	-11.68820000	-1.48140000	-1.66830000
	H	-11.25490000	-1.06620000	-0.01720000
	C	-0.76740000	7.66440000	-3.54630000
	H	-1.21840000	8.47900000	-4.12020000
	H	-0.12090000	8.13170000	-2.79310000
	O	2.69160000	-0.58330000	1.82840000
	O	-3.56070000	-4.97570000	3.16460000
	C	-10.83480000	0.50220000	-1.43920000
	H	-11.79120000	1.00350000	-1.26520000
	H	-10.62540000	0.58850000	-2.51240000
	N	1.80990000	-4.32060000	2.88780000
	O	2.08890000	-5.83910000	-1.82690000
	N	7.38680000	1.14360000	-0.02730000
	N	3.05180000	-5.11920000	-2.05970000
	O	-2.51170000	1.57190000	-1.72200000
	O	3.56070000	-4.97590000	-3.16450000
	C	2.83970000	-3.61970000	2.66260000
	H	3.29700000	-2.97570000	3.42450000
	N	-0.38680000	-2.58160000	3.63130000
	C	4.33150000	2.89540000	-2.37880000
	N	4.19980000	2.63280000	-3.82650000
	N	2.38060000	4.88660000	1.51720000
	C	4.66740000	-2.89150000	1.14610000
	H	5.09240000	-2.30430000	1.95010000
	C	3.50420000	-3.62990000	1.34560000
	N	7.20480000	-1.62840000	0.69190000
	C	1.22810000	-4.31620000	4.21760000
	H	1.71640000	-3.57550000	4.87020000
	N	-0.18160000	3.70140000	2.20340000
	C	5.39790000	2.45740000	-0.28220000
	C	-0.56930000	0.09050000	2.54760000
	H	-0.08080000	-0.03590000	3.50640000
	C	1.38630000	-5.72400000	4.81580000
	H	0.98690000	-6.44330000	4.09360000
	H	2.45320000	-5.94060000	4.92380000
	C	5.32570000	2.25120000	-1.66680000
	H	6.02840000	1.59320000	-2.15610000
	C	3.41720000	3.75100000	-1.77550000
	H	2.63660000	4.20410000	-2.36420000
	O	5.05790000	1.94780000	-4.36220000
	C	2.97830000	-4.38710000	0.29030000
	H	2.08720000	-4.97940000	0.44080000

	C	5.31870000	-2.88450000	-0.09450000
	C	-0.69770000	1.35390000	1.97320000
	C	-1.02540000	-1.05850000	1.88660000
	C	6.43540000	1.78120000	0.51490000
	H	6.33180000	1.86700000	1.60560000
	C	-1.30870000	1.47620000	0.71670000
	H	-1.36610000	2.43340000	0.22640000
	C	-0.26540000	-3.95260000	4.10080000
	H	-0.70290000	-4.66070000	3.38600000
	C	4.47330000	3.29190000	0.34200000
	H	4.48550000	3.43080000	1.41550000
	C	-0.84710000	-2.39980000	2.46520000
	H	-1.13200000	-3.23370000	1.81390000
	C	3.61910000	-4.34890000	-0.93610000
	C	4.77590000	-3.60600000	-1.15560000
	H	5.23720000	-3.61200000	-2.13360000
	C	-1.63580000	-0.93340000	0.64200000
	H	-1.98970000	-1.79750000	0.09840000
	C	2.44250000	4.75900000	0.25700000
	H	1.69400000	5.19720000	-0.41290000
	C	3.48160000	3.94720000	-0.39830000
	O	3.22840000	3.11100000	-4.40280000
	C	6.58900000	-2.16100000	-0.27920000
	H	6.98180000	-2.13550000	-1.30520000
	C	-1.77400000	0.33470000	0.08790000
	C	8.47470000	-0.97830000	0.42920000
	H	8.72530000	-1.01320000	-0.64310000
	N	-2.37350000	0.45050000	-1.25110000
	C	-0.82160000	-5.50120000	6.01880000
	H	-1.31770000	-6.21460000	5.35090000
	H	-1.32440000	-5.57120000	6.98760000
	C	0.43310000	4.76460000	2.98820000
	H	1.09860000	4.34730000	3.75880000
	C	9.72870000	1.20490000	0.64640000
	H	9.97300000	1.21380000	-0.42130000
	H	9.63030000	2.24920000	0.95620000
	C	8.37180000	0.50760000	0.82540000
	H	8.07480000	0.55310000	1.88540000
	C	-0.98170000	-4.09050000	5.44910000
	H	-0.57210000	-3.34790000	6.14280000
	H	-2.03750000	-3.85100000	5.30340000
	C	1.28560000	5.66160000	2.06770000
	H	0.62320000	6.03670000	1.26990000
	C	9.57880000	-1.67910000	1.23310000
	H	9.28680000	-1.68570000	2.28880000
	H	9.63720000	-2.72390000	0.91530000
	C	-0.66690000	5.59390000	3.67230000

	H	-1.25360000	4.93890000	4.32050000
	H	-1.34930000	5.97330000	2.90260000
	C	-0.11580000	2.52040000	2.66130000
	H	0.40210000	2.29180000	3.60060000
	C	0.65930000	-5.86770000	6.15550000
	H	1.12790000	-5.21210000	6.89990000
	H	0.77010000	-6.89160000	6.52450000
	C	-0.08250000	6.77200000	4.45550000
	H	-0.89050000	7.34800000	4.91510000
	H	0.53900000	6.39150000	5.27480000
	C	10.83490000	0.50230000	1.43880000
	H	10.62560000	0.58860000	2.51200000
	H	11.79130000	1.00360000	1.26480000
	C	10.92960000	-0.97940000	1.06060000
	H	11.25490000	-1.06620000	0.01680000
	H	11.68820000	-1.48130000	1.66790000
	C	1.86080000	6.85200000	2.84940000
	H	2.57200000	6.46510000	3.58710000
	H	2.43360000	7.47860000	2.15980000
	O	-2.69150000	-0.58330000	-1.82840000
	C	0.76700000	7.66450000	3.54620000
	H	1.21800000	8.47920000	4.12010000
	H	0.12060000	8.13180000	2.79290000
**3d** (h-to-h)	N	-3.33220000	3.16970000	1.77180000
	C	-4.01800000	1.43070000	3.25840000
	C	-4.53180000	-1.27160000	3.83560000
	H	-4.75690000	-2.30620000	4.04890000
	C	-3.83730000	2.84720000	2.88790000
	H	-4.20410000	3.59080000	3.60940000
	C	-4.77790000	1.08990000	4.37490000
	H	-5.20010000	1.84160000	5.02780000
	C	-3.27720000	-1.96020000	1.78370000
	H	-2.53590000	-1.62120000	1.04950000
	C	-5.01460000	-0.25650000	4.64010000
	C	-3.49180000	0.41170000	2.45320000
	C	-3.75210000	-0.92910000	2.72310000
	N	-0.87850000	4.77080000	1.81430000
	C	-4.29920000	6.18730000	-0.31160000
	H	-5.08550000	6.28860000	-1.06530000
	H	-4.48700000	6.95820000	0.44540000
	C	-1.89170000	4.84460000	0.77330000
	H	-1.73430000	4.06620000	0.01570000
	C	-3.27980000	4.56770000	1.37890000
	H	-3.43300000	5.24060000	2.23610000
	C	-4.38090000	4.80050000	0.32820000
	H	-5.35280000	4.64510000	0.80480000
	H	-4.27870000	4.02780000	-0.43870000

	C	-1.81800000	6.22320000	0.10500000
	H	-0.83500000	6.33540000	-0.35680000
	H	-1.89570000	6.98970000	0.88340000
	C	-2.92160000	6.41290000	-0.93740000
	H	-2.85310000	7.41460000	-1.37000000
	H	-2.77570000	5.70420000	-1.75990000
	N	-1.10180000	-4.43510000	1.78270000
	C	0.72530000	-4.13890000	3.29550000
	C	3.31630000	-3.23570000	3.90560000
	H	4.32270000	-2.91530000	4.13150000
	C	-0.58690000	-4.69280000	2.91140000
	H	-1.05820000	-5.37310000	3.63460000
	C	1.37570000	-4.60720000	4.43500000
	H	0.91890000	-5.33340000	5.09350000
	C	3.33290000	-1.84510000	1.82710000
	H	2.68610000	-1.38720000	1.06850000
	C	2.65770000	-4.14160000	4.71570000
	C	1.36660000	-3.19450000	2.48270000
	C	2.65440000	-2.75040000	2.77030000
	N	-3.73430000	-3.14150000	1.78240000
	C	-3.18160000	-6.83110000	-0.29150000
	H	-2.85950000	-7.57180000	-1.02920000
	H	-3.75740000	-7.37290000	0.46850000
	C	-3.26780000	-4.05810000	0.75360000
	H	-2.66990000	-3.53090000	-0.00090000
	C	-2.32830000	-5.10310000	1.38250000
	H	-2.83990000	-5.56710000	2.23940000
	C	-1.95520000	-6.18300000	0.35060000
	H	-1.32880000	-6.93150000	0.84400000
	H	-1.33650000	-5.71100000	-0.41770000
	C	-4.48110000	-4.70730000	0.07540000
	H	-5.06990000	-3.92430000	-0.40680000
	H	-5.11310000	-5.15170000	0.85160000
	C	-4.06970000	-5.77100000	-0.94500000
	H	-4.96170000	-6.22860000	-1.38110000
	H	-3.52380000	-5.29800000	-1.76890000
	N	4.39470000	1.26720000	1.80620000
	C	3.21170000	2.71480000	3.29660000
	C	1.11840000	4.49660000	3.88240000
	H	0.33150000	5.20380000	4.09930000
	C	4.35580000	1.86190000	2.92430000
	H	5.18110000	1.81300000	3.64850000
	C	3.28430000	3.52680000	4.42610000
	H	4.14130000	3.50980000	5.08570000
	C	-0.08450000	3.78400000	1.80800000
	H	-0.15590000	2.98250000	1.06230000
	C	2.23210000	4.39800000	4.69550000

	C	2.07280000	2.77720000	2.48260000
	C	1.03710000	3.66630000	2.75690000
	N	4.58450000	-1.65110000	1.85050000
	C	7.52260000	0.63610000	-0.24530000
	H	8.00060000	1.26890000	-0.99880000
	H	8.27830000	0.43640000	0.52380000
	C	5.16610000	-0.81000000	0.81640000
	H	4.42570000	-0.57200000	0.04220000
	C	5.59230000	0.53990000	1.42180000
	H	6.24930000	0.34960000	2.28410000
	C	6.33980000	1.38330000	0.37270000
	H	6.66850000	2.31290000	0.84530000
	H	5.62300000	1.66140000	-0.40530000
	C	6.34650000	-1.54920000	0.17540000
	H	5.97430000	-2.46770000	-0.28300000
	H	7.04030000	-1.84530000	0.96940000
	C	7.06530000	-0.68640000	-0.86380000
	H	7.91670000	-1.23530000	-1.27490000
	H	6.38930000	-0.48200000	-1.70140000
	N	-4.30370000	1.55020000	-1.81090000
	C	-3.03690000	2.93030000	-3.29540000
	C	-0.83840000	4.58410000	-3.87220000
	H	-0.00940000	5.24270000	-4.08570000
	C	-4.22970000	2.14400000	-2.92770000
	H	-5.05580000	2.14540000	-3.65260000
	C	-3.06180000	3.75520000	-4.41760000
	H	-3.91960000	3.79700000	-5.07490000
	C	0.32090000	3.78300000	-1.80520000
	H	0.34260000	2.97500000	-1.06350000
	C	-1.95810000	4.56180000	-4.68260000
	C	-1.89490000	2.91640000	-2.48340000
	C	-0.80670000	3.74180000	-2.75310000
	N	-4.66360000	-1.35590000	-1.84290000
	C	-7.46250000	1.10260000	0.24160000
	H	-7.90860000	1.76750000	0.98690000
	H	-8.22670000	0.93140000	-0.52610000
	C	-5.18690000	-0.47450000	-0.81100000
	H	-4.42650000	-0.27130000	-0.04610000
	C	-5.54060000	0.89220000	-1.42540000
	H	-6.20480000	0.73220000	-2.28820000
	C	-6.24320000	1.78200000	-0.38330000
	H	-6.52270000	2.72390000	-0.86330000
	H	-5.51250000	2.02790000	0.39250000
	C	-6.40020000	-1.14390000	-0.15410000
	H	-6.07360000	-2.07370000	0.31600000
	H	-7.11250000	-1.41540000	-0.94040000
	C	-7.07050000	-0.23300000	0.87630000

	H	-7.94720000	-0.73310000	1.29640000
	H	-6.38270000	-0.05160000	1.70940000
	N	3.52290000	2.96180000	-1.77190000
	C	4.09180000	1.19140000	-3.27040000
	C	4.42990000	-1.53430000	-3.86580000
	H	4.58940000	-2.57970000	-4.08560000
	C	4.00290000	2.61410000	-2.89150000
	H	4.41350000	3.33710000	-3.61040000
	C	4.81740000	0.81000000	-4.39660000
	H	5.27910000	1.53740000	-5.05040000
	C	3.15390000	-2.15460000	-1.80600000
	H	2.43480000	-1.77710000	-1.06840000
	C	4.96740000	-0.54700000	-4.67050000
	C	3.51030000	0.20310000	-2.46500000
	C	3.68330000	-1.14980000	-2.74450000
	N	1.17570000	4.71780000	-1.80620000
	C	4.68140000	5.90140000	0.32530000
	H	5.47370000	5.94880000	1.07800000
	H	4.91650000	6.66260000	-0.42830000
	C	2.19100000	4.72030000	-0.76480000
	H	1.98250000	3.94970000	-0.01150000
	C	3.55890000	4.35810000	-1.37210000
	H	3.75320000	5.02410000	-2.22640000
	C	4.67340000	4.51560000	-0.32120000
	H	5.63280000	4.29970000	-0.79960000
	H	4.52250000	3.74800000	0.44290000
	C	2.20680000	6.09660000	-0.08840000
	H	1.23300000	6.26750000	0.37500000
	H	2.33180000	6.86080000	-0.86280000
	C	3.32110000	6.21020000	0.95370000
	H	3.31570000	7.21140000	1.39270000
	H	3.13160000	5.50650000	1.77180000
	N	0.84080000	-4.49090000	-1.79240000
	C	-0.98410000	-4.10090000	-3.28780000
	C	-3.51840000	-3.04300000	-3.88570000
	H	-4.50490000	-2.66240000	-4.10610000
	C	0.29300000	-4.73400000	-2.90870000
	H	0.70790000	-5.45680000	-3.62530000
	C	-1.67620000	-4.54470000	-4.41240000
	H	-1.27460000	-5.30950000	-5.06320000
	C	-3.42550000	-1.62330000	-1.82910000
	H	-2.74540000	-1.19610000	-1.08170000
	C	-2.92870000	-4.00200000	-4.68760000
	C	-1.55370000	-3.10370000	-2.48450000
	C	-2.81400000	-2.58300000	-2.76540000
	N	3.54670000	-3.35890000	-1.80810000
	C	2.78040000	-7.00450000	0.28130000

	H	2.41640000	-7.71680000	1.02750000
	H	3.31470000	-7.58860000	-0.47760000
	C	3.03430000	-4.24920000	-0.77840000
	H	2.47870000	-3.68810000	-0.01590000
	C	2.02570000	-5.23390000	-1.39770000
	H	2.50030000	-5.73210000	-2.25660000
	C	1.59290000	-6.28520000	-0.35880000
	H	0.92080000	-6.99690000	-0.84650000
	H	1.00630000	-5.77370000	0.40950000
	C	4.21110000	-4.97400000	-0.11410000
	H	4.86030000	-4.23050000	0.35280000
	H	4.79960000	-5.46590000	-0.89590000
	C	3.74080000	-5.99920000	0.91940000
	H	4.60440000	-6.51090000	1.35230000
	H	3.23390000	-5.48430000	1.74340000
	H	2.96040000	0.52290000	-1.58800000
	H	-1.00320000	-2.78190000	-1.60870000
	H	-1.90340000	2.27770000	-1.60850000
	H	2.04360000	2.14590000	1.60270000
	H	-2.91350000	0.70080000	1.58400000
	H	0.84760000	-2.85350000	1.59500000
	N	-1.99900000	5.44850000	-5.86950000
	O	-3.01800000	5.43520000	-6.54580000
	O	-1.01520000	6.13660000	-6.09410000
	N	5.75280000	-0.94540000	-5.86240000
	O	5.86280000	-2.14000000	-6.09230000
	O	6.24370000	-0.05060000	-6.53650000
	N	-3.66660000	-4.49140000	-5.87610000
	O	-4.76020000	-3.99760000	-6.10430000
	O	-3.13270000	-5.36170000	-6.54970000
	N	3.34960000	-4.65680000	5.92090000
	O	2.75660000	-5.48440000	6.59860000
	O	4.46800000	-4.22610000	6.15680000
	N	2.32450000	5.27030000	5.89010000
	O	1.38670000	6.01970000	6.11570000
	O	3.33560000	5.18270000	6.57270000
	N	-5.83610000	-0.61050000	5.82170000
	O	-6.02300000	-1.79690000	6.04430000
	O	-6.27730000	0.31010000	6.49550000
**3e** (m)	N	1.92990000	4.35970000	-1.10760000
	N	4.46590000	-1.56310000	-1.42100000
	N	2.81540000	-3.84340000	-1.10870000
	N	-0.87110000	4.63480000	-1.43100000
	C	2.63230000	2.50980000	0.98130000
	H	2.19750000	3.46500000	1.24480000
	C	-2.15390000	2.91850000	1.52300000
	H	-1.69450000	3.57980000	2.24650000

	C	3.27640000	0.99400000	-0.78420000
	H	3.36010000	0.73130000	-1.83200000
	N	-3.58060000	-3.07380000	-1.42300000
	C	-2.49570000	2.32850000	-0.79350000
	H	-2.30380000	2.52750000	-1.84120000
	C	2.24720000	3.16500000	-1.39200000
	H	2.15240000	2.76890000	-2.41380000
	C	-3.49990000	1.02400000	0.97240000
	H	-4.11450000	0.17360000	1.23710000
	C	3.60410000	0.39890000	1.53290000
	H	3.94310000	-0.33100000	2.25690000
	C	3.72600000	0.08490000	0.17540000
	N	-4.74720000	-0.51340000	-1.11330000
	C	3.05300000	1.61150000	1.96030000
	C	-1.10420000	4.32300000	-0.22300000
	H	-0.68110000	4.90230000	0.61080000
	C	-2.93330000	1.83860000	1.95100000
	C	-1.45060000	-3.30810000	1.53090000
	H	-2.25240000	-3.24150000	2.25510000
	C	1.62480000	-3.51160000	-1.39360000
	H	1.33160000	-3.22740000	-2.41500000
	C	2.73610000	2.21210000	-0.38300000
	C	-1.93550000	3.17480000	0.16540000
	C	0.86300000	-3.52400000	0.97870000
	H	1.90700000	-3.63020000	1.24220000
	C	-1.78280000	-3.25010000	0.17340000
	C	4.30680000	-1.20830000	-0.21290000
	H	4.58950000	-1.86800000	0.62060000
	C	1.38440000	5.19620000	-2.15770000
	H	1.20350000	4.61690000	-3.07770000
	C	4.97860000	-2.89670000	-1.66790000
	H	5.35930000	-3.35300000	-0.73930000
	C	2.34560000	6.35110000	-2.46940000
	H	3.29340000	5.93610000	-2.82420000
	H	2.56270000	6.88140000	-1.53570000
	C	3.81570000	-3.79170000	-2.15590000
	H	3.40980000	-3.34090000	-3.07610000
	C	-3.28700000	1.25650000	-0.39190000
	C	-3.15430000	1.50640000	3.43200000
	C	1.75170000	7.31360000	-3.50290000
	H	1.63530000	6.79070000	-4.46010000
	H	2.44370000	8.14200000	-3.68100000
	C	-0.77040000	-3.31130000	-0.78620000
	H	-1.03990000	-3.24740000	-1.83380000
	C	-3.19340000	-3.11100000	-0.21480000
	H	-3.90640000	-3.02880000	0.61870000
	C	-0.57330000	6.69660000	-2.72150000

	H	-1.52710000	7.08100000	-2.34880000
	H	-0.79830000	6.11810000	-3.62420000
	C	-5.19850000	-1.40900000	-2.15910000
	H	-4.60700000	-1.27980000	-3.08000000
	C	4.32930000	-5.20340000	-2.46990000
	H	3.49420000	-5.81190000	-2.82880000
	H	4.67530000	-5.66070000	-1.53640000
	C	0.02330000	5.74950000	-1.67610000
	H	0.22160000	6.30950000	-0.74740000
	C	0.55420000	-3.45790000	-0.38550000
	C	-0.12500000	-3.44030000	1.95800000
	C	0.38660000	7.84430000	-3.05040000
	H	-0.04800000	8.48590000	-3.82240000
	H	0.52110000	8.47170000	-2.16060000
	C	-4.99360000	-2.86110000	-1.66910000
	H	-5.57730000	-2.96550000	-0.73970000
	C	-3.86750000	0.35450000	-1.39920000
	H	-3.47530000	0.46650000	-2.42080000
	C	-7.21400000	-2.16190000	-3.49950000
	H	-8.27780000	-1.98030000	-3.67880000
	H	-6.70300000	-2.00550000	-4.45740000
	C	-4.66660000	1.45460000	3.73100000
	H	-4.83520000	1.21290000	4.78440000
	H	-5.17280000	0.69860000	3.12800000
	H	-5.13530000	2.41940000	3.52180000
	C	-5.51320000	-3.85870000	-2.70880000
	H	-4.90040000	-3.76750000	-3.61230000
	H	-5.36670000	-4.87450000	-2.33080000
	C	-2.52430000	0.12860000	3.72520000
	H	-2.66220000	-0.13860000	4.77710000
	H	-1.45330000	0.14050000	3.51070000
	H	-2.97620000	-0.65360000	3.11190000
	C	2.86890000	1.96540000	3.44140000
	C	6.60830000	-4.26220000	-3.04120000
	H	7.08000000	-4.69750000	-2.15160000
	H	7.38350000	-4.20840000	-3.81120000
	C	6.10060000	-2.85560000	-2.70970000
	H	6.91250000	-2.22680000	-2.33310000
	H	5.71680000	-2.36710000	-3.61200000
	C	-2.51340000	2.54380000	4.36630000
	H	-2.92990000	3.54160000	4.20540000
	H	-1.43090000	2.59560000	4.23380000
	H	-2.70630000	2.26730000	5.40590000
	C	-6.68010000	-1.15900000	-2.47170000
	H	-7.24750000	-1.23250000	-1.53750000
	H	-6.79720000	-0.13290000	-2.83230000
	C	1.35910000	2.10460000	3.72850000

	H	1.19110000	2.35500000	4.78020000
	H	0.83740000	1.17010000	3.50980000
	H	0.90840000	2.88700000	3.11490000
	C	-6.98770000	-3.60620000	-3.03860000
	H	-7.32480000	-4.30840000	-3.80670000
	H	-7.59770000	-3.79990000	-2.14770000
	C	3.44560000	0.89060000	4.37520000
	H	4.51860000	0.75440000	4.21660000
	H	2.95110000	-0.07310000	4.23840000
	H	3.29900000	1.19310000	5.41500000
	C	5.46300000	-5.17220000	-3.49980000
	H	5.07270000	-4.80550000	-4.45710000
	H	5.83040000	-6.18680000	-3.67980000
	C	0.27380000	-3.46480000	3.43880000
	C	-0.94540000	-3.44820000	4.37310000
	H	-1.59060000	-4.31490000	4.20690000
	H	-1.54260000	-2.54320000	4.24510000
	H	-0.60970000	-3.47830000	5.41270000
	C	3.57550000	3.30220000	3.74620000
	H	4.64680000	3.22860000	3.54250000
	H	3.44420000	3.56700000	4.79940000
	H	3.17530000	4.11860000	3.14250000
	C	1.13500000	-2.21910000	3.73560000
	H	1.43450000	-2.20260000	4.78770000
	H	0.57690000	-1.30450000	3.52280000
	H	2.03860000	-2.20570000	3.12290000
	C	1.09260000	-4.73800000	3.73430000
	H	1.38630000	-4.76120000	4.78770000
	H	2.00160000	-4.78440000	3.13170000
	H	0.50390000	-5.63410000	3.52280000
**3e** (t-to-t)	N	0.87800000	4.07220000	-2.15200000
	N	2.62110000	4.99690000	-0.04010000
	N	3.01890000	-3.50250000	-4.58140000
	N	0.65520000	-1.84520000	-4.78890000
	C	4.13530000	3.41060000	0.99070000
	N	7.59440000	0.93510000	0.53000000
	N	7.71650000	-1.80170000	-0.52420000
	C	-0.20660000	1.92430000	-1.92500000
	C	-1.18750000	1.21800000	-1.22310000
	H	-1.73920000	1.74220000	-0.45600000
	C	0.25030000	-0.05020000	-3.23170000
	C	0.52690000	1.28000000	-2.92250000
	C	3.51050000	3.40880000	2.25120000
	H	2.66760000	4.06880000	2.39060000
	C	3.65600000	4.26320000	-0.10730000
	H	4.26780000	4.22740000	-1.02030000
	C	2.30680000	5.81690000	-1.19910000

	H	3.02720000	5.63470000	-2.01320000
	C	-0.01140000	3.34380000	-1.60610000
	H	-0.71350000	3.75850000	-0.87480000
	C	-0.73930000	-0.73750000	-2.51580000
	H	-0.93130000	-1.76390000	-2.79980000
	C	0.95670000	-0.69300000	-4.35010000
	H	1.75420000	-0.09190000	-4.81160000
	C	3.31120000	2.55970000	4.66980000
	C	0.90760000	5.46660000	-1.74000000
	H	0.17740000	5.65350000	-0.94080000
	C	4.91080000	-3.08870000	-2.45290000
	C	5.06830000	1.76870000	3.02080000
	H	5.44680000	1.11660000	3.80000000
	C	5.79720000	-2.97130000	-1.39090000
	C	5.71750000	1.76930000	1.78830000
	C	6.90820000	0.92720000	1.59680000
	H	7.18970000	0.30250000	2.45800000
	C	5.68670000	-3.83680000	-0.28830000
	H	6.39830000	-3.70170000	0.51320000
	C	8.80810000	0.13990000	0.48760000
	H	8.91310000	-0.47800000	1.39440000
	C	0.62190000	7.85110000	-2.54510000
	H	0.39660000	8.47440000	-3.41550000
	H	-0.15420000	8.06200000	-1.79920000
	C	-1.46490000	-0.12400000	-1.49500000
	C	1.36980000	-2.34550000	-5.94800000
	H	2.18270000	-1.66150000	-6.24230000
	C	3.91300000	-4.06970000	-2.42310000
	C	3.96200000	2.59260000	3.28170000
	C	5.24210000	2.59980000	0.76780000
	C	6.85110000	-1.94610000	-1.44050000
	H	6.85510000	-1.31660000	-2.34260000
	C	4.70210000	-4.81530000	-0.23170000
	C	2.01450000	-3.70270000	-5.60760000
	H	1.21220000	-4.37190000	-5.25600000
	C	-2.55480000	-0.90250000	-0.75210000
	C	2.94380000	-4.19430000	-3.52140000
	H	2.13790000	-4.92770000	-3.36630000
	C	8.76300000	-0.81560000	-0.71900000
	H	8.57600000	-0.20520000	-1.61730000
	C	2.15230000	3.56010000	4.79060000
	H	1.34630000	3.33950000	4.09140000
	H	2.48840000	4.58630000	4.61910000
	H	1.73470000	3.51270000	5.79940000
	C	2.76500000	1.14150000	4.93450000
	H	2.28710000	1.09870000	5.91750000
	H	3.56020000	0.39280000	4.91710000

	H	2.02640000	0.86260000	4.18380000
	C	10.10060000	-1.55690000	-0.87940000
	H	10.22900000	-2.22110000	-0.01760000
	H	10.03870000	-2.19880000	-1.76280000
	C	0.55950000	6.37030000	-2.93030000
	H	1.25730000	6.15780000	-3.74800000
	H	-0.43830000	6.10750000	-3.29000000
	C	0.38470000	-2.49130000	-7.11790000
	H	-0.45180000	-3.11640000	-6.78690000
	H	-0.03200000	-1.50680000	-7.34920000
	C	1.99280000	8.21610000	-1.96610000
	H	2.00460000	9.25980000	-1.63790000
	H	2.75190000	8.12600000	-2.75280000
	C	-3.13910000	-0.10450000	0.42360000
	H	-2.36360000	0.17020000	1.14140000
	H	-3.87570000	-0.71930000	0.94440000
	H	-3.64370000	0.80190000	0.08740000
	C	11.28600000	-0.59540000	-0.98460000
	H	12.21830000	-1.16190000	-1.06750000
	H	11.19610000	-0.00120000	-1.90220000
	C	1.05000000	-3.10650000	-8.35180000
	H	1.82110000	-2.42400000	-8.72950000
	H	0.31430000	-3.22310000	-9.15320000
	C	2.36390000	7.29810000	-0.79790000
	H	1.67600000	7.45460000	0.04030000
	H	3.36730000	7.52630000	-0.42730000
	C	3.82320000	-4.91810000	-1.32210000
	H	3.03650000	-5.66440000	-1.31080000
	C	10.00850000	1.09490000	0.38180000
	H	9.84420000	1.75580000	-0.47630000
	H	10.02490000	1.73340000	1.26970000
	C	1.69340000	-4.45490000	-8.01370000
	H	2.20670000	-4.86480000	-8.88850000
	H	0.90840000	-5.17250000	-7.74500000
	C	2.67800000	-4.31660000	-6.84970000
	H	3.09970000	-5.28870000	-6.57820000
	H	3.52000000	-3.67880000	-7.14070000
	C	4.36790000	2.91640000	5.73640000
	H	4.77630000	3.91500000	5.56030000
	H	5.19980000	2.20870000	5.74010000
	H	3.91440000	2.90270000	6.73150000
	C	-3.69000000	-1.21960000	-1.74750000
	H	-4.09140000	-0.29670000	-2.16760000
	H	-4.49970000	-1.75060000	-1.24230000
	H	-3.33370000	-1.84100000	-2.57190000
	C	4.53640000	-5.75960000	0.96570000
	C	-1.97270000	-2.21170000	-0.18880000

	H	-1.53760000	-2.83740000	-0.97130000
	H	-2.75950000	-2.78430000	0.29920000
	H	-1.19600000	-1.99890000	0.54730000
	C	11.33300000	0.34460000	0.22340000
	H	12.15540000	1.05920000	0.12370000
	H	11.53270000	-0.24130000	1.12890000
	C	5.55380000	-5.46470000	2.07790000
	H	6.58180000	-5.59290000	1.73050000
	H	5.39490000	-6.15490000	2.91010000
	H	5.44550000	-4.44770000	2.46230000
	C	3.11880000	-5.59760000	1.55420000
	H	2.98910000	-6.26710000	2.40940000
	H	2.34440000	-5.83640000	0.82220000
	H	2.95420000	-4.57300000	1.89220000
	C	4.73520000	-7.21490000	0.49330000
	H	5.73460000	-7.35180000	0.07270000
	H	4.00770000	-7.49310000	-0.27250000
	H	4.61880000	-7.90530000	1.33360000
	N	-2.54240000	5.07360000	0.21430000
	N	-0.82830000	4.00780000	2.29110000
	N	-7.51020000	1.02070000	-0.57740000
	C	-1.05460000	-0.79780000	4.36960000
	H	-1.84820000	-0.19390000	4.83430000
	C	-3.45980000	3.65190000	-2.16000000
	H	-2.61030000	4.31130000	-2.25630000
	N	-0.76100000	-1.95490000	4.80070000
	N	-3.10060000	-3.61620000	4.54290000
	C	0.17840000	1.83340000	1.99370000
	C	-4.08510000	3.58040000	-0.90180000
	N	-7.62320000	-1.77940000	0.35520000
	C	-4.90120000	-3.14950000	2.35240000
	C	-3.91670000	2.90470000	-3.23920000
	C	-5.67550000	1.99970000	-1.79800000
	C	-3.59320000	4.36030000	0.24390000
	H	-4.20630000	4.28770000	1.15390000
	C	-5.63190000	-3.87670000	0.16950000
	H	-6.32000000	-3.73630000	-0.65410000
	C	-2.98770000	-4.28680000	3.47260000
	H	-2.17320000	-5.01290000	3.32940000
	C	0.03120000	3.26710000	1.71520000
	H	0.74110000	3.67890000	0.99020000
	C	-3.81320000	-4.99590000	1.24390000
	H	-3.03840000	-5.75150000	1.25780000
	C	-5.19550000	2.76220000	-0.72770000
	C	-0.34120000	-0.15470000	3.25600000
	C	-0.81430000	5.41550000	1.92750000
	H	-0.08380000	5.60640000	1.12950000

	C	-5.75750000	-3.01130000	1.26400000
	C	-0.56850000	1.19260000	2.98350000
	C	-2.12040000	-3.82090000	5.59100000
	H	-1.30870000	-4.48620000	5.25360000
	C	1.35540000	-0.24780000	1.50250000
	C	-4.55310000	-5.79370000	-1.08920000
	C	-3.92180000	-4.14360000	2.34630000
	C	-6.85130000	1.12850000	-1.65600000
	H	-7.14300000	0.58000000	-2.56460000
	C	-8.67960000	-0.80490000	0.56630000
	H	-8.51640000	-0.23560000	1.49560000
	C	-5.03130000	2.07800000	-3.03060000
	H	-5.41280000	1.47590000	-3.84760000
	C	1.12700000	1.10960000	1.26540000
	H	1.69350000	1.63140000	0.50740000
	C	-6.79890000	-1.97220000	1.30030000
	H	-6.83640000	-1.37900000	2.22600000
	C	-4.66280000	-4.87910000	0.13800000
	C	-3.25410000	2.93630000	-4.62130000
	C	-8.71300000	0.20660000	-0.59280000
	H	-8.79620000	-0.36550000	-1.53130000
	C	-2.20550000	5.82880000	1.41000000
	H	-2.92860000	5.62790000	2.21720000
	C	-2.08590000	3.93060000	-4.68120000
	H	-2.41490000	4.94980000	-4.46070000
	H	-1.65900000	3.93080000	-5.68730000
	H	-1.28810000	3.66720000	-3.98690000
	C	-2.71550000	1.52740000	-4.94690000
	H	-2.23150000	1.52600000	-5.92790000
	H	-3.51560000	0.78400000	-4.96730000
	H	-1.98380000	1.21090000	-4.20430000
	C	-4.29680000	3.35140000	-5.68010000
	H	-4.70080000	4.34300000	-5.46000000
	H	-5.13310000	2.65050000	-5.72750000
	H	-3.83280000	3.38290000	-6.67000000
	C	-0.51810000	-2.61270000	7.13200000
	H	-0.10740000	-1.62870000	7.37560000
	H	0.32480000	-3.23430000	6.81030000
	C	0.61120000	-0.86110000	2.51010000
	H	0.76420000	-1.90210000	2.76320000
	C	-0.42750000	6.26170000	3.14780000
	H	-1.12170000	6.03590000	3.96500000
	H	0.56660000	5.95600000	3.48390000
	C	-1.83990000	-4.58290000	7.99700000
	H	-1.05060000	-5.29920000	7.73790000
	H	-2.36690000	-4.99660000	8.86200000
	C	2.40520000	-1.05390000	0.73240000

	C	-1.20170000	-3.23610000	8.35190000
	H	-0.47820000	-3.35730000	9.16360000
	H	-1.97840000	-2.55580000	8.72210000
	C	-9.92600000	1.14360000	-0.46200000
	H	-9.93280000	1.82370000	-1.31860000
	H	-9.78330000	1.76410000	0.42950000
	C	-10.01340000	-1.56460000	0.66680000
	H	-10.12080000	-2.18680000	-0.22860000
	H	-9.96000000	-2.24860000	1.51850000
	C	-1.81970000	8.18340000	2.27740000
	H	-2.57290000	8.07810000	3.06780000
	H	-1.80770000	9.24050000	1.99520000
	C	-2.80590000	-4.44040000	6.81760000
	H	-3.65310000	-3.80440000	7.09700000
	H	-3.22190000	-5.41180000	6.53510000
	C	-5.88130000	-6.55690000	-1.27250000
	H	-6.71990000	-5.87310000	-1.41680000
	H	-6.09770000	-7.17460000	-0.39700000
	H	-5.82460000	-7.21040000	-2.14790000
	C	-4.27510000	-4.93460000	-2.34010000
	H	-4.19450000	-5.57240000	-3.22510000
	H	-3.34080000	-4.38050000	-2.22940000
	H	-5.07160000	-4.21020000	-2.51810000
	C	-11.24600000	0.37600000	-0.36320000
	H	-12.07620000	1.07840000	-0.24300000
	H	-11.42550000	-0.16670000	-1.29930000
	C	-0.45290000	7.75820000	2.82370000
	H	0.31970000	7.97890000	2.07700000
	H	-0.20030000	8.33880000	3.71590000
	C	-11.21000000	-0.62090000	0.79850000
	H	-11.14090000	-0.07100000	1.74510000
	H	-12.13760000	-1.19950000	0.83840000
	C	3.09460000	-0.22270000	-0.35990000
	H	3.63620000	0.62590000	0.05920000
	H	2.37580000	0.14910000	-1.09300000
	H	3.81610000	-0.84940000	-0.88720000
	C	-3.41850000	-6.82010000	-0.95100000
	H	-3.57100000	-7.47990000	-0.09300000
	H	-2.44550000	-6.33340000	-0.84450000
	H	-3.38080000	-7.44470000	-1.84680000
	C	-2.22510000	7.32740000	1.07470000
	H	-1.53970000	7.50130000	0.23780000
	H	-3.22470000	7.59800000	0.72260000
	C	1.72950000	-2.26200000	0.05690000
	H	2.47640000	-2.86280000	-0.46000000
	H	0.99250000	-1.92720000	-0.67480000
	H	1.22170000	-2.90350000	0.78010000

	C	3.47960000	-1.54090000	1.72790000
	H	3.96080000	-0.69100000	2.21600000
	H	4.24410000	-2.11710000	1.20280000
	H	3.04670000	-2.17490000	2.50550000
	C	-1.48650000	-2.46310000	5.94910000
	H	-2.30610000	-1.78400000	6.23650000
	H	-1.29560000	1.76630000	3.54610000
	H	-5.70040000	2.69490000	0.22860000
	H	5.75160000	2.59430000	-0.18830000
	H	1.28260000	1.83830000	-3.46270000
	H	4.96850000	-2.43090000	-3.31180000
	H	-4.97270000	-2.49390000	3.21190000
**3e** (h-to-h)	N	-1.84290000	4.70000000	0.35480000
	C	-3.51560000	3.58240000	-0.95210000
	C	-4.36470000	1.90980000	-3.01330000
	H	-4.64990000	1.24580000	-3.82220000
	C	-3.07590000	4.47130000	0.13490000
	H	-3.87260000	4.91290000	0.75450000
	C	-4.88020000	3.41370000	-1.22070000
	H	-5.59310000	3.95200000	-0.60470000
	C	-1.98830000	1.30910000	-3.51930000
	H	-1.00780000	1.22660000	-3.03010000
	C	-5.32930000	2.57020000	-2.24670000
	C	-2.57420000	2.87640000	-1.70680000
	C	-2.99570000	2.05050000	-2.74830000
	N	-2.06060000	3.64920000	2.98830000
	C	-6.81700000	2.31920000	-2.52690000
	C	0.16040000	7.25480000	2.34220000
	H	0.97030000	7.92720000	2.03420000
	H	-0.63600000	7.89060000	2.75600000
	C	-0.97060000	4.53030000	2.60630000
	H	-0.13110000	3.96280000	2.17310000
	C	-1.47500000	5.49690000	1.50790000
	H	-2.34430000	6.03690000	1.92380000
	C	-0.37030000	6.49060000	1.12470000
	H	-0.76170000	7.18340000	0.37050000
	H	0.43950000	5.92800000	0.64410000
	C	-7.13250000	2.65400000	-4.00010000
	H	-8.19140000	2.46620000	-4.21340000
	H	-6.53900000	2.04830000	-4.69160000
	H	-6.92240000	3.70800000	-4.21260000
	C	-7.73430000	3.16840000	-1.63240000
	H	-7.56340000	4.24040000	-1.78150000
	H	-7.59290000	2.94100000	-0.57090000
	H	-8.78160000	2.96150000	-1.87790000
	C	-7.12000000	0.82770000	-2.26110000
	H	-8.17670000	0.60900000	-2.45670000

	H	-6.90290000	0.56860000	-1.21920000
	H	-6.51310000	0.17540000	-2.89700000
	C	-0.45650000	5.30660000	3.82540000
	H	-0.08810000	4.58980000	4.56810000
	H	-1.29810000	5.84060000	4.28600000
	C	0.65020000	6.28940000	3.42790000
	H	0.99390000	6.84260000	4.31020000
	H	1.51490000	5.72580000	3.05020000
	N	-1.90010000	-2.00120000	-4.20520000
	C	-3.53170000	-2.60600000	-2.55030000
	C	-4.32370000	-3.57850000	-0.06160000
	H	-4.58690000	-3.95770000	0.91990000
	C	-3.12490000	-2.10940000	-3.87410000
	H	-3.94160000	-1.82750000	-4.55750000
	C	-4.88900000	-2.78060000	-2.24880000
	H	-5.61880000	-2.53030000	-3.01180000
	C	-1.93360000	-3.67940000	0.67320000
	H	-0.97500000	-3.16980000	0.50600000
	C	-5.30970000	-3.25710000	-0.99930000
	C	-2.56970000	-2.88880000	-1.57640000
	C	-2.96230000	-3.39240000	-0.33650000
	N	-2.20700000	0.78590000	-4.65900000
	C	-6.78870000	-3.39830000	-0.61600000
	C	0.00310000	-1.57320000	-7.47550000
	H	0.82430000	-2.15670000	-7.90930000
	H	-0.81490000	-1.58680000	-8.21060000
	C	-1.11410000	0.03870000	-5.25700000
	H	-0.24300000	-0.00390000	-4.58190000
	C	-1.57910000	-1.41840000	-5.49290000
	H	-2.46670000	-1.37220000	-6.14880000
	C	-0.46650000	-2.22820000	-6.17210000
	H	-0.83120000	-3.24500000	-6.35900000
	H	0.36750000	-2.32040000	-5.46550000
	C	-7.07490000	-4.84780000	-0.17000000
	H	-8.12780000	-4.95580000	0.11590000
	H	-6.46320000	-5.13830000	0.68950000
	H	-6.86690000	-5.55250000	-0.98260000
	C	-7.73220000	-3.05680000	-1.78040000
	H	-7.56690000	-3.71540000	-2.64040000
	H	-7.61230000	-2.02030000	-2.11200000
	H	-8.77220000	-3.18300000	-1.46040000
	C	-7.08500000	-2.43150000	0.55220000
	H	-8.13450000	-2.51060000	0.86030000
	H	-6.89160000	-1.39530000	0.25500000
	H	-6.45650000	-2.64830000	1.42200000
	C	-0.67620000	0.68670000	-6.57660000
	H	-0.34520000	1.71190000	-6.37450000

	H	-1.54640000	0.75850000	-7.24240000
	C	0.44290000	-0.12300000	-7.24050000
	H	0.73690000	0.34860000	-8.18590000
	H	1.32980000	-0.11190000	-6.59120000
	N	-1.79870000	-2.65140000	3.85380000
	C	-3.44470000	-0.92820000	3.56850000
	C	-4.25800000	1.70570000	3.16400000
	H	-4.52890000	2.74310000	3.00040000
	C	-3.02670000	-2.31920000	3.80110000
	H	-3.83720000	-3.05670000	3.91540000
	C	-4.80310000	-0.58600000	3.59870000
	H	-5.52500000	-1.37420000	3.78560000
	C	-1.88070000	2.40110000	2.81560000
	H	-0.93320000	2.00540000	2.42360000
	C	-5.23490000	0.73110000	3.38870000
	C	-2.49240000	0.05820000	3.29550000
	C	-2.89650000	1.37960000	3.10740000
	N	-2.11250000	-4.44560000	1.67400000
	C	-6.71680000	1.12780000	3.36060000
	C	0.15590000	-5.67040000	5.10060000
	H	0.98590000	-5.74130000	5.81400000
	H	-0.64760000	-6.31350000	5.48840000
	C	-1.00590000	-4.57310000	2.60680000
	H	-0.14830000	-3.95350000	2.29710000
	C	-1.46300000	-4.05410000	3.99200000
	H	-2.34160000	-4.65420000	4.28920000
	C	-0.33770000	-4.22180000	5.02180000
	H	-0.69830000	-3.88100000	5.99950000
	H	0.48260000	-3.55030000	4.73990000
	C	-6.97590000	2.24130000	4.39740000
	H	-8.03080000	2.53950000	4.37810000
	H	-6.37220000	3.13180000	4.19800000
	H	-6.73670000	1.89390000	5.40840000
	C	-7.64700000	-0.05390000	3.67810000
	H	-7.45230000	-0.46470000	4.67510000
	H	-7.54380000	-0.86150000	2.94620000
	H	-8.68940000	0.28200000	3.65500000
	C	-7.05790000	1.64980000	1.94710000
	H	-8.11020000	1.95350000	1.89290000
	H	-6.88630000	0.87140000	1.19590000
	H	-6.43980000	2.51190000	1.67690000
	C	-0.54310000	-6.03230000	2.70090000
	H	-0.21550000	-6.36430000	1.70930000
	H	-1.40010000	-6.65920000	2.98060000
	C	0.58950000	-6.18350000	3.72220000
	H	0.90230000	-7.23290000	3.78260000
	H	1.46300000	-5.61140000	3.37890000

	N	1.87450000	4.52360000	-1.27660000
	C	3.53710000	3.68050000	0.23500000
	C	4.36890000	2.44790000	2.59270000
	H	4.64790000	1.95760000	3.51910000
	C	3.10560000	4.33950000	-1.00800000
	H	3.90710000	4.64920000	-1.69730000
	C	4.89990000	3.56090000	0.53770000
	H	5.61850000	3.96220000	-0.16920000
	C	1.98660000	1.97350000	3.19750000
	H	1.01080000	1.79260000	2.72680000
	C	5.34010000	2.93520000	1.71290000
	C	2.58960000	3.14290000	1.11090000
	C	3.00190000	2.54050000	2.29910000
	N	2.12720000	2.97900000	-3.65010000
	C	6.82500000	2.73200000	2.04270000
	C	-0.10770000	6.63440000	-3.74990000
	H	-0.92580000	7.34710000	-3.59000000
	H	0.69320000	7.18490000	-4.26490000
	C	1.03340000	3.91680000	-3.46530000
	H	0.18430000	3.44190000	-2.94680000
	C	1.51860000	5.07980000	-2.56700000
	H	2.39170000	5.53430000	-3.06840000
	C	0.40540000	6.12240000	-2.39980000
	H	0.78360000	6.95050000	-1.78880000
	H	-0.41030000	5.66020000	-1.83030000
	C	7.14220000	3.35450000	3.41890000
	H	8.19930000	3.20460000	3.66840000
	H	6.54300000	2.90600000	4.21700000
	H	6.94130000	4.43140000	3.41380000
	C	7.75210000	3.37390000	0.99820000
	H	7.59100000	4.45540000	0.92620000
	H	7.61100000	2.93740000	0.00420000
	H	8.79690000	3.21190000	1.28490000
	C	7.11300000	1.21470000	2.08510000
	H	8.16680000	1.02990000	2.32600000
	H	6.89590000	0.75240000	1.11620000
	H	6.49730000	0.71030000	2.83670000
	C	0.54380000	4.44270000	-4.82060000
	H	0.19430000	3.59530000	-5.42150000
	H	1.39350000	4.88550000	-5.35700000
	C	-0.57370000	5.47540000	-4.63930000
	H	-0.90210000	5.84740000	-5.61730000
	H	-1.44380000	4.98850000	-4.17660000
	N	1.86930000	-1.12480000	4.51490000
	C	3.49860000	-2.05620000	3.01790000
	C	4.28690000	-3.49960000	0.76820000
	H	4.54800000	-4.06530000	-0.11950000

	C	3.09390000	-1.30920000	4.21890000
	H	3.91150000	-0.91090000	4.84070000
	C	4.85450000	-2.30230000	2.76410000
	H	5.58500000	-1.91770000	3.46830000
	C	1.89910000	-3.70940000	0.05080000
	H	0.94930000	-3.15970000	0.10500000
	C	5.27350000	-3.01690000	1.63320000
	C	2.53620000	-2.50910000	2.11080000
	C	2.92730000	-3.24580000	0.99310000
	N	2.19370000	1.69490000	4.42230000
	C	6.75210000	-3.25250000	1.29720000
	C	-0.04170000	-0.06280000	7.63200000
	H	-0.86790000	-0.54720000	8.16650000
	H	0.77310000	0.06290000	8.36000000
	C	1.09370000	1.08410000	5.14890000
	H	0.22480000	0.91560000	4.49100000
	C	1.54920000	-0.30240000	5.66450000
	H	2.43580000	-0.13520000	6.30210000
	C	0.43030000	-0.96040000	6.48310000
	H	0.79000000	-1.92270000	6.86590000
	H	-0.40070000	-1.18590000	5.80330000
	C	7.01580000	-4.76570000	1.14630000
	H	8.06800000	-4.94400000	0.89500000
	H	6.40290000	-5.20920000	0.35590000
	H	6.79230000	-5.29380000	2.07970000
	C	7.69510000	-2.70370000	2.37980000
	H	7.51380000	-3.17740000	3.35110000
	H	7.59090000	-1.62040000	2.49980000
	H	8.73440000	-2.90700000	2.09980000
	C	7.07150000	-2.53830000	-0.03500000
	H	8.12090000	-2.69360000	-0.31270000
	H	6.89530000	-1.46080000	0.05210000
	H	6.44410000	-2.91060000	-0.85110000
	C	0.65410000	1.97720000	6.31590000
	H	0.32870000	2.94530000	5.91830000
	H	1.52150000	2.17230000	6.96020000
	C	-0.47240000	1.31620000	7.11760000
	H	-0.76870000	1.96340000	7.95190000
	H	-1.35600000	1.20480000	6.47330000
	N	1.79750000	-3.36700000	-3.28810000
	C	3.46360000	-1.64250000	-3.33960000
	C	4.30230000	1.01290000	-3.44350000
	H	4.58330000	2.05990000	-3.47880000
	C	3.02980000	-3.04740000	-3.29080000
	H	3.83050000	-3.80250000	-3.24020000
	C	4.82590000	-1.32490000	-3.41750000
	H	5.54110000	-2.14070000	-3.43610000

	C	1.92650000	1.78070000	-3.27060000
	H	0.96180000	1.46610000	-2.84800000
	C	5.27010000	0.00410000	-3.46180000
	C	2.51970000	-0.61310000	-3.27940000
	C	2.93630000	0.71590000	-3.34560000
	N	2.06900000	-4.66380000	-0.77440000
	C	6.75640000	0.38500000	-3.49210000
	C	-0.20480000	-6.54950000	-3.87940000
	H	-1.02880000	-6.76000000	-4.57190000
	H	0.59020000	-7.27480000	-4.10650000
	C	0.96370000	-4.96170000	-1.66920000
	H	0.12160000	-4.26930000	-1.50780000
	C	1.43830000	-4.76160000	-3.12890000
	H	2.30730000	-5.42600000	-3.28270000
	C	0.31570000	-5.12660000	-4.10920000
	H	0.68760000	-5.00940000	-5.13390000
	H	-0.49390000	-4.39720000	-3.98450000
	C	7.04010000	1.26890000	-4.72510000
	H	8.09780000	1.55660000	-4.75080000
	H	6.44340000	2.18600000	-4.71450000
	H	6.80960000	0.72990000	-5.65070000
	C	7.67650000	-0.84450000	-3.55730000
	H	7.48860000	-1.44200000	-4.45640000
	H	7.55510000	-1.49100000	-2.68220000
	H	8.72230000	-0.52000000	-3.58650000
	C	7.08690000	1.17400000	-2.20570000
	H	8.14210000	1.47240000	-2.19790000
	H	6.89690000	0.56230000	-1.31720000
	H	6.47600000	2.07820000	-2.12110000
	C	0.46370000	-6.39490000	-1.44900000
	H	0.11750000	-6.49140000	-0.41380000
	H	1.30680000	-7.08760000	-1.57070000
	C	-0.66240000	-6.74210000	-2.42870000
	H	-0.99740000	-7.77320000	-2.26350000
	H	-1.52660000	-6.09240000	-2.23050000
	H	1.49200000	-2.28030000	2.30110000
	H	1.47000000	-0.87330000	-3.18280000
	H	1.53940000	3.20750000	0.84420000
	H	-1.44610000	-0.22700000	3.24130000
	H	-1.52220000	2.98710000	-1.46260000
	H	-1.52430000	-2.71320000	-1.81070000

7. References

[1] a) Cherutoi, J. K.; Sandifer, J. D.; Pokharel, U. R.; Fronczek, F. R.; Pakhomova, S.; Maverick, A. W. Externally and Internally Functionalized Copper(II) β-Diketonate Molecular Squares. *Inorg. Chem*. **2015**, *54*, 7791; b) Jennings K. F. 229. An Attempted Synthesis of Perhydro-2 : 8-Methylenepyridocoline. *J. Chem. Soc*. **1957**, 1172; c) Bennani, Y. L.; Marron, K. S.; Mais, D. E.; Flatten, K.; Nadzan, A. M.; Boehm, M. F. Synthesis and Characterization of a Highly Potent and Selective Isotopically Labeled Retinoic Acid Receptor Ligand, ALRT1550. *J. Org. Chem*. **1998**, *63*, 543; d) Catala, L.; Turek, P.; Le Moigne, J.; De Cian, A.; Kyritsakas, N. Synthesis of nitronyl- and imino-nitroxide-triradicals interconnected by phenyl ethynyl spacer. *Tetrahedron Lett*. **2000**, *41*, 1015.

[2] a) Gawroński, J.; Kołbon, H.; Kwit, M.; Katrusiak, A., Designing Large Triangular Chiral Macrocycles: Efficient [3 + 3] Diamine−Dialdehyde Condensations Based on Conformational Bias. *J. Org. Chem*., **2000**, *65*, 5768; b) Troć, A.; Gajewy, J.; Danikiewicz, W.; Kwit, M., Specific Noncovalent Association of Chiral Large-Ring Hexaimines: Ion Mobility Mass Spectrometry and PM7 Study. *Chem. Eur. J.* **2016**, *22*, 13258.

[3] a) Becke, A. D. Density-functional Thermochemistry. III. The Role of Exact Exchange. *J. Chem. Phys.* **1993**, *98*, 5648; b) Lee, C.; Yang, W.; Parr, R. G. Development of the Colle-Salvetti Correlation-Energy Formula into a Functional of the Electron Density. *Phys. Rev. B* **1988**, *37*, 785; c) Becke, A. D. Density-Functional Exchange-Energy Approximation with Correct Asymptotic Behavior. *Phys. Rev. A* **1988**, *38*, 3098; d) Perdew, J. P. Density-Functional Approximation for the Correlation Energy of the Inhomogeneous Electron Gas. *Phys. Rev. B* **1986**, *33*, 8822.

[4] a) Grimme, S.; Ehrlich, S.; Goerigk, L. Effect of the Damping Function in Dispersion Corrected Density Functional Theory. *J. Comp. Chem*. **2011**, *32*, 1456; b) Antony, J.; Sure, R.; Grimme, S. Using Dispersion-Corrected Density Functional Theory to Understand Supramolecular Binding Thermodynamics. *Chem. Commun*. **2015**, *51*, 1764.

[5] Scalmani, G.; Frisch, M. J. Continuous surface charge polarizable continuum models of solvation. I. General formalism. *J. Chem. Phys.* **2010,** *132*, 114110.

[6] Boys, S. F.; Bernardi, F. The Calculation of Small Molecular Interactions by the Differences of Separate Total Energies. Some Procedures with Reduced Errors. *Mol. Phys.* **1970**, *19*, 553.

[7] Kwit, M.; Gawronski, J.; Boyd, D. R.; Sharma, N. D.; Kaik, M. Circular Dichroism, Optical Rotation and Absolute Configuration of 2-Cyclohexenone-*cis*-diol Type Phenol Metabolites: Redefining the Role of Substituents and 2-Cyclohexenone Conformation in Electronic Circular Dichroism Spectra. *Org. Biomol. Chem.* **2010**, *8*, 5635 and literature cited therein.

[8] a) Zhao, Y.; Truhlar, D. G. The M06 suite of density functionals for main group thermochemistry, thermochemical kinetics, noncovalent interactions, excited states, and

transition elements: two new functionals and systematic testing of four M06-class functionals and 12 other functionals. *Theor. Chem. Acc.* **2008**, 120, 215; (b) Jacquemin, D.; Perpète, E. A.; Ciofini, I.; Adamo, C.; Valero, R.; Zhao, Y.; Truhlar, D. G. On the Performances of the M06 Family of Density Functionals for Electronic Excitation Energies. *J. Chem. Theor.* **2010**, *6*, 2071.

[9] Goerigk, L.; Grimme, S. Calculation of Electronic Circular Dichroism Spectra with Time-Dependent Double-Hybrid Density Functional Theory. *J. Phys. Chem. A* **2009**, *113*, 767.

[10] a) Adamo, C.; Barone, V. Toward reliable density functional methods without adjustable parameters: The PBE0 model. *J. Chem. Phys.* **1999**, *110*, 6158; b) Tawada, Y.; Tsuneda, T.; Yanagisawa, S.; Yanai, T.; Hirao, K. A long-range-corrected time-dependent density functional theory. *J. Chem. Phys.* **2004**, *120*, 8425.

[11] Yanai, T.; Tew, D.; Handy, N. A new hybrid exchange–correlation functional using the Coulomb-attenuating method (CAM-B3LYP). *Chem. Phys. Lett.* **2004**, *393*, 51.

[12] Harada, N.; Stephens, P. ECD cotton effect approximated by the Gaussian curve and other methods. *Chirality* **2010**, *22*, 229.

[13] Frisch, M. J.; Trucks, G. W.; Schlegel, H. B.; Scuseria, G. E.; Robb, M. A.; Cheeseman, J. R.; Scalmani, G.; Barone, V.; Mennucci, B.; Petersson, G. A.; Nakatsuji, H.; Caricato, M.; Li, X.; Hratchian, H. P.; Izmaylov, A. F.; Bloino, J.; Zheng, G.; Sonnenberg, J. L.; Hada, M.; Ehara, M.; Toyota, K.; Fukuda, R.; Hasegawa, J.; Ishida, M.; Nakajima, T.; Honda, Y.; Kitao, O.; Nakai, H.; Vreven, T.; Montgomery, J. A., Jr.; Peralta, J. E.; Ogliaro, F.; Bearpark, M. J.; Heyd, J.; Brothers, E. N.; Kudin, K. N.; Staroverov, V. N.; Kobayashi, R.; Normand, J.; Raghavachari, K.; Rendell, A. P.; Burant, J. C.; Iyengar, S. S.; Tomasi, J.; Cossi, M.; Rega, N.; Millam, N. J.; Klene, M.; Knox, J. E.; Cross, J. B.; Bakken, V.; Adamo, C.; Jaramillo, J.; Gomperts, R.; Stratmann, R. E.; Yazyev, O.; Austin, A. J.; Cammi, R.; Pomelli, C.; Ochterski, J. W.; Martin, R. L.; Morokuma, K.; Zakrzewski, V. G.; Voth, G. A.; Salvador, P.; Dannenberg, J. J.; Dapprich, S.; Daniels, A. D.; Farkas, Ö.; Foresman, J. B.; Ortiz, J. V.; Cioslowski, J.; Fox, D. J. Gaussian 09, revision D.01; Gaussian, Inc.: Wallingford, CT, 2009.

[14] Harada, N.; Nakanishi, K. Circular Dichroism Spectroscopy: Exciton Coupling in Organic Stereochemistry. University Science Books: Mill Valley, CA, 1983.

[15] Janiak, A.; Petryk, M.; Barbour, L. J.; Kwit, M. Readily Prepared Inclusion Forming Chiral Calixsalens. *Org. Biomol. Chem*. **2016**, *14*, 669.

[16] Sheldrick, G. M. SHELXT - Integrated space-group and crystal-structure determination. *Acta Crystallogr. A*, **2015**, *71*, 3.

[17] Barbour, L. J. X-Seed 4: updates to a program for small-molecule supramolecular crystallography. *J. Appl. Cryst*., **2020**, *53*, 1141.

[18] Spek, A. L. Structure validation in chemical crystallography. *Acta Cryst. D*, **2009**, *65*, 148.

[19] POV-RayTM for Windows, version 3.7, Persistence of Vision Raytracer Pty Ltd, Williamstown, Australia, 2004, http://www.povray.org

[20] Macrae, C. F.; Bruno, I. J.; Chisholm, J. A.; Edgington, P. R.; McCabe, P.; Pidcock, E.; Rodriguez-Monge, L.; Taylor, R.; van de Streek, J.; Wood, P. A. Mercury CSD 2.0 - new features for the visualization and investigation of crystal structures. *J. Appl. Cryst.*, **2008**, *41*, 466.

[21] Connolly, M. L. Solvent-Accessible Surfaces of Proteins and Nucleic Acids. *Science*, **1983**, *221*, 709.
